# Engineering of indole-based tethered biheterocyclic alkaloid meridianin into β-carboline-derived tetracyclic polyheterocycles via amino functionalization/6-*endo* cationic π-cyclization

**DOI:** 10.3762/bjoc.8.220

**Published:** 2012-11-08

**Authors:** Piyush Kumar Agarwal, Meena Devi Dathi, Mohammad Saifuddin, Bijoy Kundu

**Affiliations:** 1 Division of Medicinal and Process Chemistry, Central Drug Research Institute, CSIR, Lucknow, 226001, India, Phone: +91 522 2612411-18; Fax: +91 522 2623405

**Keywords:** cyclization, indole, meridianin, natural products, nitrogen heterocycles

## Abstract

A mild, efficient and versatile method has been developed for the construction of a functionalized natural product, meridianin, and its post conversion to pyrimido-β-carboline by cationic π- cyclization. The strategy involves the introduction of an amino group at the C-5 of the pyrimidine ring and utilizing the nucleophilictiy of the C-2 in the indole ring to facilitate cationic π-cyclization.

## Introduction

Indole is an important pharmacophore present in many natural and designed polyheterocyclic synthetic products of therapeutic importance [1–3]. The range of applications for these therapeutically relevant indole-based polyheterocycles includes protein kinase C inhibitors, 5-HT agonists, melatonin agonists, and glucocorticoid receptor modulators, displaying cytotoxic, antiviral, antimicrobial, antiparasitic, anti-inflammatory, antiserotonin, Ca^2+^/calmodulin antagonistic [4] and antitopoisomerase-I activities [5–16].

**Figure 1:**
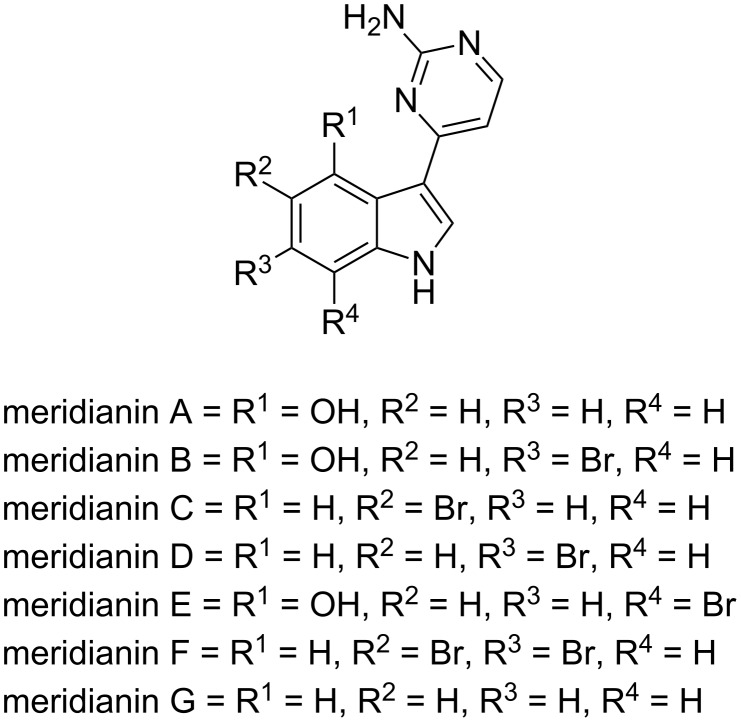
Structure of meridianins A–G.

The presence of a heterocyclic ring at the position 3 of the indole represents an important class of marine alkaloids, such as oxazole (martefragin [17], amazol [18]), imidazole (topsentins [19–20] and nortopsentins [21–23]), dihydroimidazole (discodermindole [24]), oxadiazine (alboinon [25]), piperazine (dragmacidon [26]), maleimide (didemidines [25]), and pyrimidine (meridianins) [27–29]. Among these, meridianins (Figure 1), the tethered biheterocycles isolated from the south Atlantic tunicate *Aplidium meridianum,* represent an interesting synthetic target owing to their biological implication involving cytotoxicity towards murine-tumor cell lines [30–31] and as potent protein-kinase inhibitor [32].

In continuation of our ongoing studies pertaining to the synthesis of the indole-based natural products, i.e., isocryptolepine, δ-carbolines, and α-carbolines; and the indole-based polyheterocycles, i.e., indolo-quinolines, pericyclic indolo-benzazepines, iodo-indoloazepinones, indoloindazole, and azepino-indole [33–43], we were prompted to transform the indole-based alkaloid meridianins into annulated indole-based polyheterocycles as novel chemprobes.

For the synthesis of meridianin-inspired indole-based annulated polyheterocycles, we proposed to transform tethered biheterocycles into β-carboline-based polyheterocycles, a new prototype hitherto not reported in the literature. β-Carbolines are some of the most widely distributed alkaloids, associated with activities ranging from antineoplastic (tubulin binding) [44–46], anticonvulsive, hypnotic and anxiolytic (benzodiazepine receptor ligands) [47], antimicrobial as well as topoisomerase-II inhibition [48] to inhibition of cGMP-dependent processes [49–50].

In this communication, we report engineering of naturally occurring tethered indole-based biheterocyclic alkaloid meridianins into β-carboline-derived tetracyclic polyheterocycles by amino functionalization of the pyrimidine ring followed by 6-*endo* cationic π-cyclization.

## Results and Discussion

**Scheme 1:**
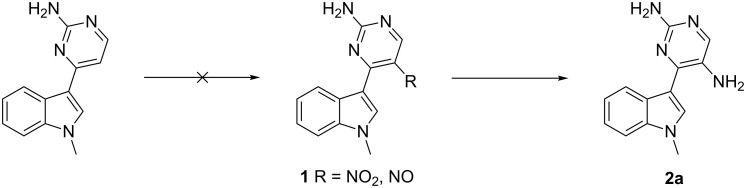
Synthesis of functionalized meridianin with an amino group at position 5.

Our studies commenced with the functionalization of the pyrimidine ring in the meridianin followed by the application of cationic π-cyclization [51–60] to generate an additional pyridine ring as part of β-carboline-derived tetracyclic polyheterocycles. Initial attempts to synthesize amino-functionalized substrate **2a** by introducing a nitro or nitroso group at the para position (position 5) of the 2-aminopyrimidine linked to the indole at C-3 position, using various reported protocols, either failed to produce the desired nitro compound or resulted in an inseparable mixture of compounds (Scheme 1).

**Scheme 2:**
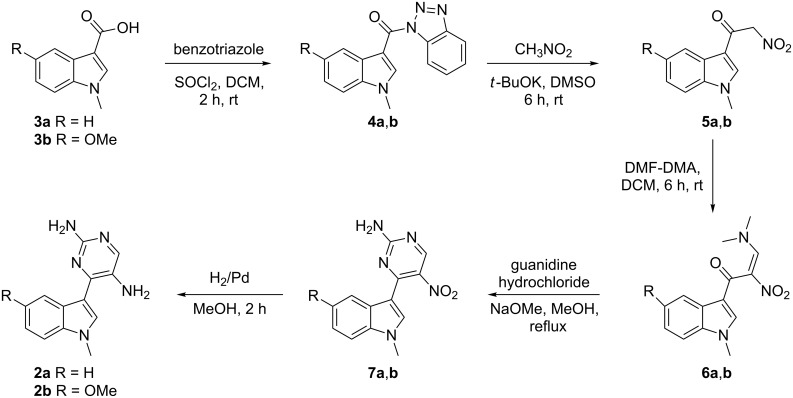
Synthesis of a functionalized meridianin with an amino group at position 5.

**Scheme 3:**
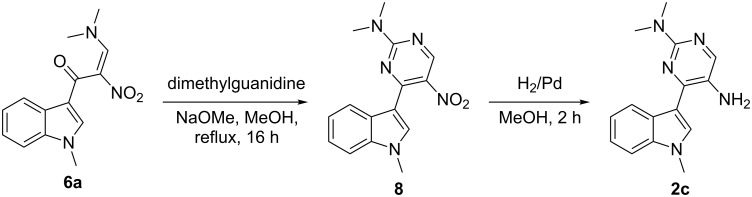
Synthesis of substrate for the modified Pictet–Spengler reaction.

This led us to attempt the synthesis of substrate **2a** using an alternate strategy in a manner that may lead to the generation of a nitro pyrimidine ring tethered to the indole at position 3 (Scheme 2). For the generation of pyrimidine rings we used the modified procedure described previously by us [56]. Initial attempts to synthesize α-nitroketone **5a** by using different protocols failed. To achieve this, the carboxylic group of 1-methyl-1*H*-indole-3-carboxylic acid (**3a**) was activated by preparing *N*-acylbenzotriazole [61]. Next, 1*H*-benzo[*d*][1,2,3]triazol-1-yl-(1-methyl-1*H*-indol-3-yl)methanone (**4a**) was converted into its α-nitroketone **5a** by treating with nitromethane in the presence of potassium *tert*-butoxide in DMSO [62]. 1-(1-Methyl-1*H*-indol-3-yl)-2-nitroethanone (**5a**) was reacted with *N*,*N*-dimethylformamide dimethyl acetal to form (*E*)-3-(dimethylamino)-1-(1-methyl-1*H*-indol-3-yl)-2-nitroprop-2-en-1-one (**6a**) [63–66], which was then converted to 4-(1-methyl-1*H*-indol-3-yl)-5-nitropyrimidin-2-amine (**7a**) in the presence of guanidine hydrochloride. Finally, the desired substrate 4-(1-methyl-1*H*-indol-3-yl)pyrimidin-2,5-diamine (**2a**) was obtained by reducing **7a** with H_2_/Pd in methanol. Synthesis of substrate **2b** with diversity in the phenyl ring of the indole was carried out in a similar manner as described for **2a**. In order to introduce diversity in the pyrimidine ring of the designed substrate **2**, compound **6a** was treated with 1,1-dimethylguanidine sulfate salt to get *N*,*N*-dimethyl-4-(1-methyl-1*H*-indol-3-yl)-5-nitropyrimidin-2-amine (**8**). The nitro group in **8** was finally reduced with H_2_/Pd in methanol to give *N*^2^,*N*^2^-dimethyl-4-(1-methyl-1*H*-indol-3-yl)pyrimidine-2,5-diamine (**2c**) as a new substrate (Scheme 3).

**Scheme 4:**
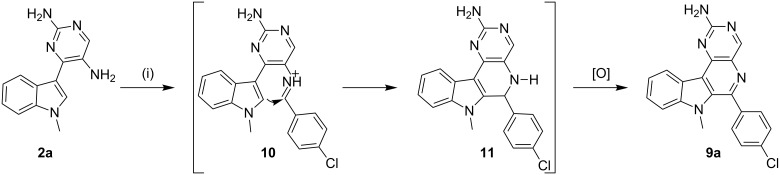
The Pictet–Spengler reaction involving substrate **2a**. Reagents and conditions: (i) RCHO, 2% triflic acid in DMF, 120 °C, 16 h.

After successfully accomplishing the synthesis of amino-functionalized meridianins **2**, we next examined their abilities to undergo 6-*endo* cyclization in the presence of aldehydes (Scheme 4). Initially we treated the substrate **2a** with 4-chlorobenzaldehyde using a variety of traditional Pictet–Spengler protocols involving 1% TFA in DCM at rt, with *p*-TsOH in toluene under reflux, and AcOH in ethanol under reflux. The conditions failed to favor cationic π-cyclization even after prolonged stirring and resulted in imines as the only isolated product (Table 1, entries 1–6). Since the role of Brønsted acids are considered to be an important factor [67] in promoting cationic π-cyclization by enhancing the electrophilicity of the imines, we envisaged that employing stronger acids may facilitate 6-*endo* cyclization. Accordingly, the amine **2a** was treated with 4-chlorobenzaldehyde by using strong Brønsted acids, such as triflic acid and methanesulfonic acid (MSA), to facilitate π-cyclization, and progress of the reaction was monitored by TLC.

**Table 1:** Optimization of the reaction conditions for conversion of substrate **2a** to **9a**.

Entry	Brønsted acid	Solvent	Temp (^o^C)	Time (h)	Product^a^

1	1% TFA	DCM	rt	24	0
2	1% triflic acid	DMF	rt	24	0
3	*p*-TsOH	Toluene	120	24	0
4	AcOH	Ethanol	100	24	0
5	2% MSA	ACN	80	24	0
6	2% MSA	DMF	120	16	0
7	2% triflic acid	ACN	80	24	27
8	2% triflic acid	DMF	120	16	87
9	5% triflic acid	DMF	120	16	80
10	1% triflic acid	DMF	120	16	75
11	0.5% triflic acid	DMF	120	16	35

^a^based on HPLC.

Although no conversion was observed for **2a** in the presence of MSA (Table 1, entry 5 and entry 6) in CH_3_CN and DMF at 80 °C and 120 °C, respectively, the presence of 2% triflic acid in DMF (Table 1, entry 8) favored complete conversion of **2a** into **9a** in >87% purity based on HPLC. The crude product obtained after workup was purified by silica gel column chromatography with EtOAc/hexane as an eluent in 84% isolated yield. An increase or decrease in the concentration of triflic acid was found to be detrimental (Table 1, entries 9–11). Similarly, switching solvents from DMF to ACN in the presence of 2% triflic acid reduced the yield to 27% (Table 1, entry 7). The scope and limitation of the strategy with substrate **2a** and **2c** was established by synthesizing 11 compounds based on pyrimido-β-carbolines **9a–k** (Table 2), using eight aromatic aldehydes.

**Table 2:** Pyrimido-β-carbolines based on **9**.

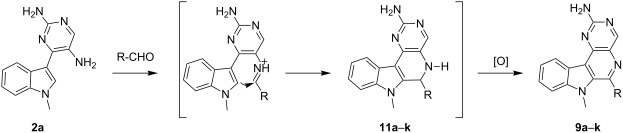						

Entry	Substrate	R-CHO	Pictet–Spengler products **9**, R		Yield (%)^a^	*t*_R_ (min)^b^

1	**2a**	4-Cl-C_6_H_4_-CHO	**9a**	4-Cl-C_6_H_4_	84	14.92
2	**2a**	4-OEt-C_6_H_4_-CHO	**9b**	4-OEt-C_6_H_4_	82	14.14
3	**2a**	4-OMe-C_6_H_4_-CHO	**9c**	4-OMe-C_6_H_4_	78	13.25
4	**2a**	4-Br-C_6_H_4_-CHO	**9d**	4-Br-C_6_H_4_	85	13.90
5	**2a**	3,4-diCl-C_6_H-CHO	**9e**	3,4-diCl-C_6_H_3_	80	14.50
6	**2c**	4-Br-C_6_H_4_-CHO	**9f**	4-Br-C_6_H_4_	72	15.89
7	**2c**	4-NO_2_-C_6_H_4_-CHO	**9g**	4-NO_2_-C_6_H_4_-CHO	76	15.82
8	**2c**	4-OMe-C_6_H_4_-CHO	**9h**	4-OMe-C_6_H_4_-CHO	79	13.45
9	**2c**	3,4-diOMe-C_6_H_3_-CHO	**9i**	3,4-diOMe-C_6_H_3_	81	12.95
10	**2c**	4-Cl-C_6_H_4_-CHO	**9j**	4-Cl-C_6_H_4_	86	14.82
11	**2c**	2-Cl-C_6_H_4_-CHO	**9k**	2-Cl-C_6_H_4_	78	14.37

^a^Isolated yield. ^b^Retention time on HPLC (C18 reversed-phase column; 150 mm × 4.8 mm; 5 µm) with a linear gradient of 0–100% CH_3_CN in water over 30 min. Flow rate of 1.0 mL/min and UV detection at 220/254 nm.

**Scheme 5:**
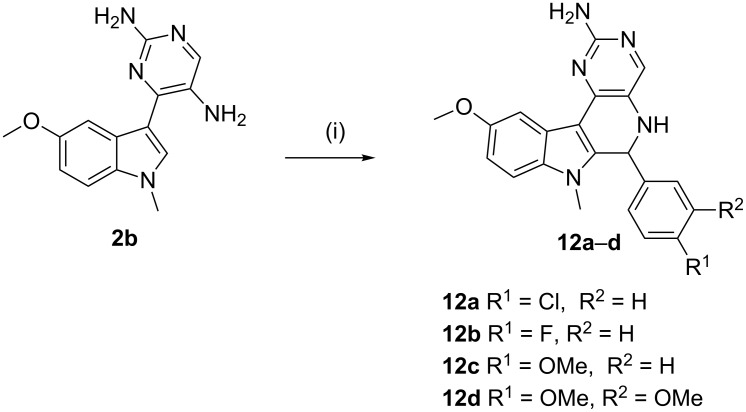
Synthesis of dihydropyrimido-β-carbolines: (i) R-CHO, 2% triflic acid in DMF, 120 °C, 16 h.

In general for all reactions, the protocol involving 2% triflic acid in DMF at 120 °C for 16 h was employed, furnishing products **9** in good isolated yields (72–86%). Pleasingly, aldehydes with either an electron-donating or -withdrawing group had no adverse effect and afforded **9** with minimal variation in yields. However, the substrate **2b** on application of the protocol involving 2% triflic acid in DMF at 120 °C produced a dihydroproduct instead of the oxidized compound. Continuing the reaction for a further 24 h failed to produce the oxidized product (Scheme 5).

**Scheme 6:**
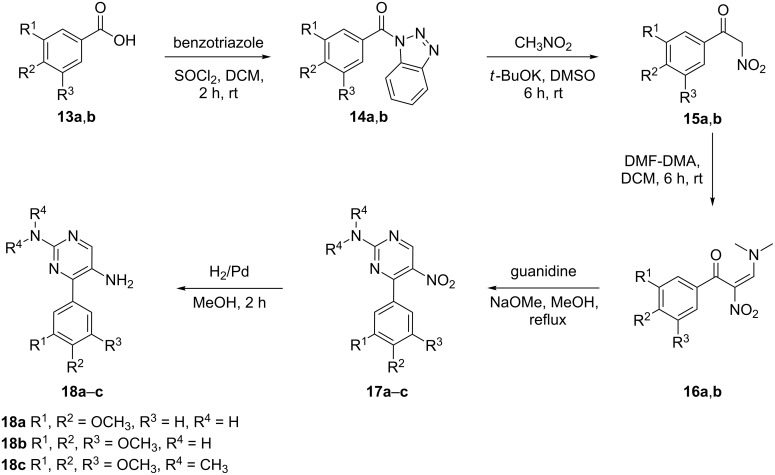
Synthesis of substrates **18a–c** for the modified Pictet–Spengler reaction.

After successfully establishing the strategy on **2a-c**, we then decided to replace the indole nucleus by another activated nucleus such as trimethoxy- and dimethoxybenzene and designed a substrate **18** (Scheme 6) for the Pictet–Spengler reaction using a similar approach as was used for the synthesis of substrates **2**.

**Scheme 7:**
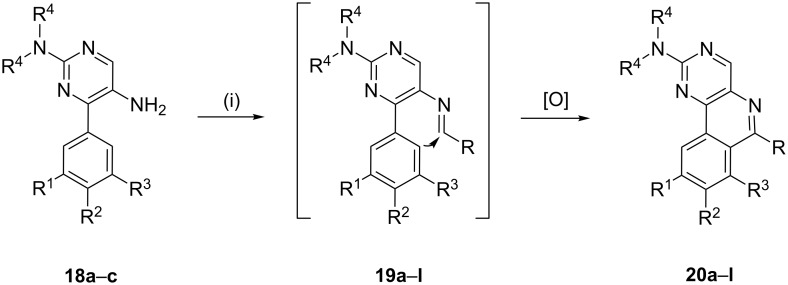
General strategy for the Pictet–Spengler reaction involving substrates **18**. Reagents and conditions: (i) R-CHO, 2% triflic acid in DMF, 120 °C, 16 h*.*

The synthesis of substrates **18** is depicted in Scheme 6. Initially, the carboxylic group of benzoic acids **13a**,**b** was activated by preparing *N*-acylbenzotriazoles **14**, which were then converted into their α-nitroketones **15** by treatment with nitromethane in the presence of potassium *tert*-butoxide in DMSO. Reaction of **15** with *N*,*N*-dimethylformamide dimethyl acetal provided **16**, which was cyclized in the presence of guanidine hydrochloride and *N*,*N*-dimethylguanidine sulfate to give the nitro products **17a**,**b** and **17c**. Finally, the desired substrates **18** were obtained by reducing **17** with H_2_/Pd in methanol. Next, substrates **18** were exposed to the cationic π-cyclization with a variety of aldehydes using the protocol involving 2% triflic acid in DMF at 120 °C (Scheme 7). The crude *endo*-cyclized product obtained after workup was purified by silica-gel column chromatography furnishing pyrimido[5,4-*c*]isoquinolines **20a–l** (Table 3) in 79–92% isolated yields.

**Table 3:** Pyrimido[5,4-*c*]isoquinolines based on **20**.

Entry	**Substrate**	R^3^-CHO	Pictet–Spengler products **20**, R^3^		Yield (%)^a^	*t*_R_ (min)^b^

1	**18a**	4-F-C_6_H_4_-CHO	**20a**	4-F-C_6_H_4_-CHO	85	20.12
2	**18a**	4-Br-C_6_H_4_-CHO	**20b**	4-Br-C_6_H_4_-CHO	87	21.95
3	**18a**	4-Cl-C_6_H_4_-CHO	**20c**	4-Cl-C_6_H_4_-CHO	83	25.25
4	**18b**	4-OMe-C_6_H_4_-CHO	**20d**	4-OMe-C_6_H_4_-CHO	89	21.42
5	**18b**	4-Cl-C_6_H_4_-CHO	**20e**	4-Cl-C_6_H_4_-CHO	92	20.65
6	**18b**	4-F-C_6_H_4_-CHO	**20f**	4-F-C_6_H_4_-CHO	91	21.98
7	**18b**	4-Br-C_6_H_4_-CHO	**20g**	4-Br-C_6_H_4_-CHO	87	22.85
8	**18c**	4-NO_2_-C_6_H_4_-CHO	**20h**	4-NO_2_-C_6_H_4_-CHO	81	20.46
9	**18c**	3,4-diOMe-C_6_H_3_-CHO	**20i**	3,4-diOMe-C_6_H_3_-CHO	84	21.56
10	**18c**	3,4-diCl-C_6_H_3_-CHO	**20j**	3,4-diCl-C_6_H_3_-CHO	83	21.45
11	**18c**	4-Br-C_6_H_4_-CHO	**20k**	4-Br-C_6_H_4_-CHO	79	20.12
12	**18c**	4-OMe-C_6_H_4_-CHO	**20l**	4-OMe-C_6_H_4_-CHO	87	19.89

^a^Isolated yield. ^b^Retention time on HPLC (C18 reversed-phase column; 150 mm × 4.8 mm; 5 µm) with a linear gradient of 0–100% CH_3_CN in water over 30 min. Flow rate of 1.0 mL/min and UV detection at 220/254 nm.

## Conclusion

In conclusion, we have developed a mild and efficient protocol for the synthesis of pyrimido[5,4-*c*]isoquinolones and pyrimido-β-carbolines using a modified Pictet–Spengler strategy. Our method offers a unique opportunity to introduce rigidity in these flexible molecules, as well as diversity, which enables the design of a library based on the natural product. Our methodology further demonstrated a broad substrate scope and reactivity, and thus it can be applied for the synthesis of a variety of novel polycyclic skeletons based on natural products.

## Supporting Information

File 1 Experimental section, ^1^H and ^13^C NMR spectra of the compounds **2a–c**, **4a–7a**, **4b–7b**, **8**, **9a–k**, **12a–d**, **14a–18a**, **14b–16b**, **17–18c**, **17b–18b**, **20a–l**.
