# Supplementary material for: Engineering of indole-based tethered biheterocyclic alkaloid meridianin into β-carboline-derived tetracyclic polyheterocycles via amino functionalization/6-endo cationic π-cyclization
Source: Beilstein J Org Chem. 2012 Nov 8;8:1901–8. doi: 10.3762/bjoc.8.220 (PMC3511029; doi:10.3762/bjoc.8.220)
Supplement: File 1 — Experimental section, 1H and 13C NMR spectra of the compounds 2a–c, 4a–7a, 4b–7b, 8, 9a–k, 12a–d, 14a–18a, 14b–16b, 17–18c, 17b–18b, 20a–l. [file Beilstein_J_Org_Chem-08-1901-s001.pdf]

Supporting Information

for

Engineering of indole-based tethered biheterocyclic alkaloid meridianin into β-carboline-derived tetracyclic polyheterocycles via amino functionalization/6-*endo* cationic π-cyclization

Piyush K. Agarwal, Meena D. Dathi, Mohammad Saifuddin and Bijoy Kundu*

Address: Division of Medicinal and Process Chemistry, Central Drug Research Institute, CSIR, Lucknow, 226001, India, Phone: +91 522 2612411-18; Fax: +91 522 2623405.

E-mail: Bijoy Kundu – bijoy_kundu@yahoo.com

Experimental section, ^{1}H and ^{13}C NMR spectra of the compounds

2a–c, **4a–7a**, **4b–7b**, **8**, **9a–k**, **12a–d**, **14a–18a**, **14b–16b**, **17–18c**, **17b–18b**, **20a–l**.

Experimental section

General consideration: All solvents were commercially available and used without purification. All products were characterized by ^{1}H NMR, ^{13}C NMR, ESMS, IR and HPLC. Analytical TLC was performed using 2.5 x 5 cm plated coated with a 0.25 mm thickness of silica gel 60F-254 Merck, and visualization was accomplished with UV light and iodine. Column chromatography was performed using silica gel 60 Thomas Baker (100–200 mesh). ^{1}H NMR spectra (200/300 MHz) are reported as follows: chemical shifts in ppm downfield from TMS as internal standard (δ scale), multiplicity [br = broad, s = singlet, δ doublet, t = triplet, q = quartet, m = multiplet, o = overlapped, integration and coupling constant (Hz)]. All ^{13}C NMR spectra (50/75/100 MHz) were recorded at 25 °C with complete proton decoupling and reported in ppm. The purity and characterization of these compounds were further established using HR/EI mass spectrometry. Elemental analyses were performed on a Carlo Erba 1108 microanalyzer or Elementar's Vario EL III microanalyzer. The micro-analyses were performed at Sophisticated Analytical Instrument Facility Division, CDRI. Analytical HPLC were performed on C-18 reversed-phase column (150 mm x 4.8 mm) (model number is LiChrospher 100, RP-18e (5 µm), Lot L552433). HRMS–EI mass spectra were done on JEOL-600H at 70 eV. Melting points reported are uncorrected.

Synthesis of 1*H*-benzo[*d*][1,2,3]triazol-1-yl-(1-methyl-1*H*-indol-3-yl)methanone (4a): To a solution of benzotriazole (22.8 mmol) in CH_2Cl_2 (20 mL) was added $SOCl_2$ (5.70 mmol) at 25 °C with stirring. After 0.5 h, 1-methyl-1*H*-indole-3-carboxylic acid (**3a**) (5.70 mmol) was added in one portion and stirring was continued for 2 h. The white precipitate was filtered off and washed with CH_2Cl_2 (2 × 25 mL). The combined organic solution was washed with aq 2 N NaOH (3 ×

20 mL), dried over Na_2SO_4 and the solvent was removed under reduced pressure. The reaction mixture was then subjected to column chromatography (100–200 silica gel) using ethyl acetate:hexane (4:1, v/v) as eluent.

Yield = 92%; white solid; mp 160–162 °C; R_f 0.60 (3:7 EtOAc:hexane); IR (KBr) ν_{max}: 3063, 1592 cm^{-1}; ^1H NMR (300 MHz, $CDCl_3$) δ 8.82 (1H, s, ArH), 8.58-8.50 (2H, m, ArH), 8.14 (1H, d, J = 8.25 Hz, ArH), 7.69-7.64 (1H, m, ArH), 7.53-7.48 (1H, m, ArH), 7.46-7.37 (3H, m, ArH), 3.96 (3H, s, CH_3); ^{13}C NMR (75 MHz, $CDCl_3$) δ 160.9, 145.6, 139.9, 136.9, 132.6, 129.9, 128.6, 125.7, 123.8, 123.2, 122.2, 119.8, 115.1, 110.1, 106.4, 33.9; ESMS *m/z*: 277.1 [M^+ + 1]; Anal. calcd for $C_{16}H_{12}N_4O$: C, 69.55; H, 4.38; N, 20.28; found: C, 69.50; H, 4.34; N, 20.22.

Compounds **4b**, **14a**, and **14b** were synthesized analogously to compound **4a** starting from **3b**, **13a** and **13b**, respectively.

1*H*-Benzo[*d*][1,2,3]triazol-1-yl-(5-methoxy-1-methyl-1*H*-indol-3-yl)methanone (4b): Yield = 70%; orange solid; mp 164–166 °C; R_f 0.54 (3:7 EtOAc:hexane); IR (KBr) ν_{max}: 3019, 2921, 1669, cm^{-1}; ^1H NMR (300 MHz, $CDCl_3$) δ 8.77 (1H, s, ArH), 8.51 (1H, d, J = 8.34 Hz, ArH), 8.14 (1H, d, J = 8.25 Hz, ArH), 8.05 (1H, d, J = 2.28 Hz, ArH), 7.66 (1H, t, J = 7.27 Hz, ArH), 7.50 (1H, t, J = 7.29 Hz, ArH), 7.32 (1H, d, J = 8.91 Hz, ArH), 7.03 (1H, dd, J_1 = 2.34 Hz, J_2 = 8.85 Hz, ArH), 3.98 (3H, s, OCH_3), 3.93 (3H, s, CH_3); ^{13}C NMR (75 MHz, $CDCl_3$) δ 161.1, 156.9, 145.6, 139.8, 132.6, 131.9, 129.8, 129.6, 125.7, 119.9, 115.1, 114.2, 110.9, 105.9, 103.6, 55.9, 34.1; ESMS *m/z*: 307.1 [M^+ + 1]; Anal. calcd for $C_{17}H_{14}N_4O_2$: C, 66.66; H, 4.61; N, 18.29; found: C, 66.60; H, 4.65; N, 18.22.

1*H*-Benzo[*d*][1,2,3]triazol-1-yl-(3,4-dimethoxyphenyl)methanone (14a): Yield = 90%; white solid; mp 130-132 °C; R_f 0.75 (3:7 EtOAc:hexane); IR (KBr) ν_{max}: 2938, 1689, 1268 cm^{-1}; ^1H NMR (300 MHz, $CDCl_3$) δ 8.36 (1H, d, J = 8.25 Hz, ArH), 8.16 (1H, d, J = 8.19 Hz, ArH),

8.04 (1H, dd, J_1 = 1.92 Hz, J_2 = 8.52 Hz, ArH), 7.83 (1H, s, ArH), 7.72-7.67 (1H, m, ArH), 7.53 (1H, t, J = 7.68 Hz, ArH), 7.03 (1H, d; J = 8.55 Hz, ArH), 4.00 (3H, s, OCH_3), 3.99 (3H, s, OCH_3); ^{13}C NMR (75 MHz, $CDCl_3$) δ 165.4, 153.9, 148.7, 145.5, 132.5, 130.1, 127.2, 126.0, 123.3, 119.9, 114.8, 114.1, 110.2, 56.1, 56.0; ESMS *m/z*: 284.1 [M^+ + 1]; Anal. calcd for $C_{15}H_{13}N_3O_3$: C, 63.60; H, 4.63; N, 14.83; found: C, 63.65; H, 4.60; N, 14.85.

1*H*-Benzo[*d*][1,2,3]triazol-1-yl-(3,4,5-trimethoxyphenyl)methanone (14b): Yield = 95%; white solid; mp 126-128 ^{o}C, R_f 0.70 (3:7 EtOAc:hexane); IR (KBr) ν_{max}: 3010, 1572 cm^{-1}; ^{1}H NMR (300 MHz, $CDCl_3$) δ 8.39 (1H, d, J = 8.25 Hz, ArH), 8.19 (1H, d, J = 8.16 Hz, ArH), 7.73 (1H, t, J = 7.65 Hz, ArH), 7.59-7.55 (3H, m, ArH), 4.01 (3H, s, OCH_3), 3.97 (6H, s, 2 x OCH_3); ^{13}C NMR (75 MHz, $CDCl_3$) δ 165.8, 152.9, 145.7, 143.2, 132.6, 130.4, 126.3, 125.9, 120.2, 114.9, 109.6, 61.1, 56.4; ESMS *m/z*: 314.1 [M^+ + 1]; Anal. calcd for $C_{16}H_{15}N_3O_4$: C, 61.34; H, 4.83; N, 13.41; found: C, 61.31; H, 4.87; N, 13.43.

Synthesis of 1-(1-methyl-1*H*-indol-3-yl)-2-nitroethan-1-one (5a): A mixture of the nitromethane (5.43 mmol) and potassium *tert*-butoxide (5.43 mmol) in DMSO (10 mL) was stirred for 10 min, while the temperature was maintained at 10 °C. 1*H*-benzo[*d*][1,2,3]triazol-1-yl-(1-methyl-1*H*-indol-3-yl)methanone (**4a**) (3.62 mmol) in DMSO (10 mL) was added dropwise to the resulting solution, and the mixture was stirred for 2 h at 10 °C and then at room temperature for 6 h. The mixture was poured into water (20 mL), acidified with acetic acid 10%; and then extracted with ethyl acetate (3 x 10 mL). The extract was washed with water, dried over Na_2SO_4 and the solvent was removed under reduced pressure. The residue was placed in a silica-gel column and eluted with hexanes/EtOAc 10:1 to give 1-(1-methyl-1*H*-indol-3-yl)-2-nitroethan-1-one (**5a**).

Yield = 82%; yellow solid; mp 192–194 °C; R_f 0.16 (3:7 EtOAc:hexane); IR (KBr) ν_{max}: 3060, 2951 cm^{-1}; ^{1}H NMR (300 MHz, DMSO-d_6) δ 8.42 (1H, s, ArH), 8.13 (1H, d, J = 6.69 Hz, ArH), 7.61 (1H, d, J = 7.41 Hz, ArH), 7.38-7.28 (2H, m, ArH), 6.15 (2H, s, CH_2), 3.90 (3H, s, CH_3); ^{13}C NMR (75 MHz, DMSO-d_6) δ 180.7, 139.2, 137.5, 125.6, 123.7, 123.0, 121.1, 112.3, 111.2, 82.0, 33.6; ESMS m/z: 219.0 [M$^+$ + 1]; Anal. calcd for $C_{11}H_{10}N_2O_3$: C, 60.55; H, 4.62; N, 12.84; found: C, 60.59; H, 4.65; N, 12.87.

Compounds **5b**, **15a**, and **15b** were synthesized analogously to compound **5a** starting from **4b**, **14a** and **14b**, respectively.

1-(5-Methoxy-1-methyl-1*H*-indol-3-yl)-2-nitroethan-1-one (5b): Yield = 75% yellow solid; mp 170–172 °C; R_f 0.20 (3:7 EtOAc:hexane); IR (KBr) ν_{max}: 3117, 1642, 1216 cm^{-1}; ^{1}H NMR (300 MHz, DMSO-d_6) δ 8.34 (1H, s, ArH), 7.62 (1H, d, J = 2.31 Hz, ArH), 7.52 (1H, d, J = 8.91 Hz, ArH), 6.97 (1H, dd, J_1 = 2.43 Hz, J_2 = 8.91 Hz, ArH), 6.12 (2H, s, CH_2), 3.86 (3H, s, OCH_3), 3.79 (3H, s, CH_3); ^{13}C NMR (75 MHz, DMSO-d_6) δ 180.6, 156.5, 139.1, 132.5, 126.6, 113.4, 112.2, 112.1, 103.0, 82.0, 55.5, 33.8; ESMS m/z: 249.0 [M$^+$ + 1]; Anal. calcd for $C_{12}H_{12}N_2O_4$: C, 58.06; H, 4.87; N, 11.29; found: C, 58.00; H, 4.84; N, 11.34.

1-(3,4-Dimethoxyphenyl)-2-nitroethan-1-one (15a): Yield = 75%; yellow solid; mp 136–138 °C R_f 0.42 (3:7 EtOAc:hexane); IR (KBr) ν_{max}: 3444, 1683, 1268 cm^{-1}; ^{1}H NMR (300 MHz, $CDCl_3$) δ 7.49 (1H, d, J = 1.29 Hz, ArH), 7.40 (1H, d, J = 8.97 Hz, ArH), 6.92 (1H, d, J = 9.31 Hz, ArH), 5.84 (2H, s, CH_2), 3.97 (3H, s, OCH_3), 3.94 (3H, s, OCH_3); ^{13}C NMR (75 MHz, $CDCl_3$) δ 184.2, 154.9, 149.7, 126.5, 123.1, 110.4, 110.1, 81.0, 56.2, 56.1; ESMS m/z: 226.0 [M$^+$ + 1]; Anal. calcd for $C_{10}H_{11}NO_5$: C, 53.33; H, 4.92; N, 6.22; found: C, 53.37; H, 4.90; N, 6.25.

2-Nitro-1-(3,4,5-trimethoxyphenyl)ethan-1-one (15b): Yield = 76%; yellow solid; mp 134–136 °C; R_f 0.10 (3:7 EtOAc:hexane); IR (KBr) ν_{max}: 3053, 1559 cm^{-1}; ^1H NMR (300 MHz, $CDCl_3$) δ 7.09 (2H, s, ArH), 5.86 (2H, s, CH_2), 3.94 (3H, s, OCH_3), 3.91 (6H, s, 2 x OCH_3); ^{13}C NMR (75 MHz, $CDCl_3$) δ 184.8, 153.5, 144.4, 128.5, 105.9, 81.4, 61.2, 56.5; ESMS *m/z*: 256.0 [M^+ + 1]; Anal. calcd for $C_{11}H_{13}NO_6$: C, 51.77; H, 5.13; N, 5.49; found: C, 51.74; H, 5.17; N, 5.51.

Synthesis of (*E*)-3-(dimethylamino)-1-(1-methyl-1*H*-indol-3-yl)-2-nitroprop-2-en-1-one (6a): A mixture of 1-(1-methyl-1*H*-indol-3-yl)-2-nitroethan-1-one (**5a**) (4.57 mmol) and DMF-DMA (4.57 mmol) in DCM (10 mL) was stirred at rt for 6 h. The solvent was evaporated and the crude product was triturated with ether to get pure (*E*)-3-(dimethylamino)-1-(1-methyl-1*H*-indol-3-yl)-2-(nitromethyl)prop-2-en-1-one (**6a**).

Yield = 95%; yellow solid; mp 170–172 °C; R_f 0.15 (3:7 EtOAc:hexane); IR (KBr) ν_{max}: 3010, 2951; ^1H NMR (300 MHz, DMSO-d_6) δ 8.41 (1H, s, ArH), 8.12-8.11 (2H, m, ArH), 7.55 (1H, d, *J* = 7.32 Hz, ArH), 7.34-7.24 (2H, m, ArH), 3.85 (3H, s, CH_3), 3.31 (3H, s, CH_3), 2.73 (3H, s, CH_3); ^{13}C NMR (75 MHz, DMSO-d_6) δ 181.8, 150.9, 139.1, 137.7, 125.6, 124.3, 123.2, 122.5, 120.9, 117.4, 110.9, 47.1, 33.2; ESMS *m/z*: 274.0 [M^+ + 1]; Anal. calcd for $C_{14}H_{15}N_3O_3$: C, 61.53; H, 5.53; N, 15.38; found: C, 61.50; H, 5.54; N, 15.36.

Compounds **6b**, **16a**, and **16b** were synthesized analogously to compound **6a** starting from **5b**, **15a** and **15b**, respectively.

(*E*)-3-(Dimethylamino)-1-(5-methoxy-1-methyl-1*H*-indol-3-yl)-2-nitroprop-2-en-1-one (6b): Yield = 85%; brown solid; mp 138–140 °C; R_f 0.08 (3:7 EtOAc:hexane); IR (KBr) ν_{max}: 3229, 2365, 1638 cm^{-1}; ^1H NMR (300 MHz, DMSO-d_6) δ 8.39 (1H, s, ArH), 8.03 (1H, s, ArH), 7.63 (1H, d, *J* = 1.86 Hz, ArH), 7.45 (1H, d, *J* = 8.88 Hz, ArH), 6.94 (1H, dd, J_1 = 2.37 Hz, J_2 =

8.85 Hz, ArH), 3.82 (3H, s, OCH_3), 3.81 (3H, s, CH_3), 3.20 (3H, s, CH_3), 2.73 (3H, s, CH_3); ^{13}C NMR (75 MHz, DMSO-d_6) δ 182.1, 156.5 151.2, 139.7, 133.1, 126.8, 124.6, 117.5, 113.2, 112.2, 103.3, 55.8, 33.8; ESMS *m/z*: 304.1 [M^+ + 1]; Anal. calcd for $C_{15}H_{17}N_3O_4$: C, 59.40; H, 5.65; N, 13.85; found: C, 59.44; H, 5.69; N, 13.82.

(*E*)-1-(3,4-Dimethoxyphenyl)-3-(dimethylamino)-2-nitroprop-2-en-1-one (16a): Yield = 78%; yellow solid; mp 154–156 °C; R_f 0.16 (3:7 EtOAc:hexane); IR (KBr) ν_{max}: 3010, 1652, cm^{-1}; ^{1}H NMR (300 MHz, $CDCl_3$) δ 8.34 (1H, s, ArH), 7.46-7.44 (2H, m, ArH), 6.87 (1H, d, *J* = 8.2 Hz, ArH), 3.93 (3H, s, OCH_3), 3.93 (3H, s, OCH_3), 3.33 (3H, s, CH_3), 2.76 (3H, s, CH_3); ^{13}C NMR (400 MHz, $CDCl_3$) δ 186.9, 153.4, 152.6, 149.2, 131.3, 124.2, 123.9, 110.4, 110.2, 56.0, 55.9, 47.9, 41.5; ESMS *m/z*: 281.1 [M^+ + 1]; Anal. calcd for $C_{13}H_{16}N_2O_5$: C, 55.71; H, 5.75; N, 9.99; found: C, 55.69; H, 5.72; N, 9.94.

(*E*)-3-(Dimethylamino)-2-nitro-1-(3,4,5-trimethoxyphenyl)prop-2-en-1-one (16b): Yield = 90%; yellow solid; mp 132–134 °C; R_f 0.10 (3:7 EtOAc:hexane); IR (KBr) ν_{max}: 3410, 1653 cm^{-1}; ^{1}H NMR (300 MHz, $CDCl_3$) δ 8.35 (1H, s, ArH), 7.10 (2H, s, ArH), 3.89 (9H, s, CH_3), 3.37 (3H, s, CH_3), 2.78 (3H, s, CH_3); ^{13}C NMR (75 MHz, $CDCl_3$) δ 187.0, 153.2, 152.8, 142.6, 133.5, 123.9, 107.0, 106.1, 60.9, 56.3, 48.0, 41.9; ESMS *m/z*: 311.1 [M^+ + 1]; Anal. calcd for $C_{14}H_{18}N_2O_6$: C, 54.19; H, 5.85; N, 9.03; found: C, 54.25; H, 5.81; N, 9.00.

Compounds **7a**,**b** and **17a**–**c** were synthesized in a similar manner as described in reference [57].

4-(1-Methyl-1*H*-indol-3-yl)-5-nitropyrimidin-2-amine (7a):

Yield = 76%; yellow solid; mp >250 °C; R_f 0.17 (3:7 EtOAc:hexane); IR (KBr) ν_{max}: 3015, 2960; ^{1}H NMR (300 MHz, DMSO-d_6) δ 8.85 (1H, s, ArH), 7.97-7.94 (2H, m, ArH), 7.76 (2H, s, NH_2), 7.53 (1H, d, *J* = 8.04 Hz, ArH), 7.28 (1H, t, *J* = 7.09 Hz, ArH), 7.17 (1H, t, *J* = 7.10 Hz, ArH), 3.87 (3H, s, CH_3); ^{13}C NMR (75 MHz, DMSO-d_6) δ 162.9, 156.8, 156.6, 136.8, 134.6, 133.5,

126.1, 122.3, 121.4, 121.2, 110.6, 109.3, 33.1; ESMS *m/z*: 270.1 [M^+ + 1]; Anal. calcd for $C_{13}H_{11}N_5O_2$: C, 57.99; H, 4.12; N, 26.01; found: C, 57.92; H, 4.17; N, 26.05.

4-(5-Methoxy-1-methyl-1*H*-indol-3-yl)-5-nitropyrimidin-2-amine (7b): Yield = 77% yellow solid; mp 210–212 °C; R_f 0.15, (3:7 EtOAc:hexane); IR (KBr) ν_{max}: 3018, 1641, 1216 cm^{-1}; ^1H NMR (300 MHz, DMSO-d_6) δ 8.83 (1H, s, ArH), 7.89 (1H, s, ArH), 7.74 (2H, s, NH_2) 7.47-7.42 (2H, m, ArH), 6.90 (1H, dd, J_1 = 2.28 Hz, J_2 = 8.82 Hz, ArH), 3.84 (3H, s, OCH_3), 3.80 (3H, s, CH_3); ^{13}C NMR (75 MHz, DMSO-d_6) δ 162.9, 156.7, 156.6, 155.1, 134.87, 133.3, 131.9, 126.8, 112.0, 111.3, 108.7, 103.7, 55.4, 33.2; ESMS *m/z*: 300.1 [M^+ + 1]; Anal. calcd for $C_{14}H_{13}N_5O_3$: C, 56.18; H, 4.38; N, 23.40; found: C, 56.15; H, 4.40; N, 23.43.

4-(3,4-Dimethoxyphenyl)-5-nitropyrimidin-2-amine (17a): Yield = 86%; yellow solid; mp 236–238 °C; R_f 0.21 (3:7 EtOAc:hexane); IR (KBr) ν_{max}: 3093, 2365, 1647 cm^{-1}; ^1H NMR (300 MHz, DMSO-d_6) δ 8.92 (1H, s, ArH), 7.99 (2H, s, NH_2), 7.11 (1H, d, J = 1.20 Hz, ArH), 7.07-7.00 (2H, m, ArH), 3.81 (3H, s, OCH_3), 3.75 (3H, s, OCH_3); ^{13}C NMR (50 MHz, DMSO-d_6) δ 163.0, 162.2, 156.9, 150.5, 148.2, 134.4, 128.1, 121.3, 111.7, 111.1, 55.6, 55.5; ESMS *m/z*: 277.0 [M^+ + 1]; Anal. calcd for $C_{12}H_{12}N_4O_4$: C, 52.17; H, 4.38; N, 20.28; O; found: C, 52.19; H, 4.39; N, 20.32.

5-Nitro-4-(3,4,5-trimethoxyphenyl)pyrimidin-2-amine (17b): Yield = 72%; yellow solid; mp >250 °C; R_f 0.30 (3:7 EtOAc:hexane); IR (KBr) ν_{max}: 3150, 1576 cm^{-1}; ^1H NMR (300 MHz, DMSO-d_6) δ 8.92(1H, s, ArH), 6.73 (2H, s, ArH), 5.82 (2H, s, NH_2), 3.90 (3H, s, OCH_3), 3.87 (6H, s, 2 x OCH_3); ^{13}C NMR (75 MHz, DMSO-d_6) δ 163.1, 162.3, 156.7, 153.3, 140.3, 136.2, 130.3, 105.5, 60.9, 56.3; ESMS *m/z*: 307.1 [M^+ + 1]; Anal. calcd for $C_{13}H_{14}N_4O_5$: C, 50.98; H, 4.61; N, 18.29; found: C, 50.92; H, 4.65; N, 18.30.

***N,N*-Dimethyl-5-nitro-4-(3,4,5-trimethoxyphenyl)pyrimidin-2-amine (17c):** Yield = 85%; yellow solid; mp 164–166 °C; R_f 0.12 (3:7 EtOAc:hexane); IR (KBr) ν_{max}: 3015, 2926, 1612 cm^{-1}; ^{1}H NMR (300 MHz, DMSO-d_6) δ 9.05 (1H, s, ArH), 6.84 (2H, s, ArH), 3.77 (6H, s, 2 x OCH_3), 3.73 (3H, s, OCH_3), 3.28 (6H, s, 2 x CH_3); ^{13}C NMR (75 MHz, DMSO-d_6) δ 161.9, 160.3, 156.5, 152.6, 139.1, 133.4, 131.8, 105.8, 60.1, 56.1, 37.3; ESMS *m/z*: 335.1 [M$^+$ + 1]; Anal. calcd for $C_{15}H_{18}N_4O_5$: C, 53.89; H, 5.43; N, 16.76; found: C, 53.91; H, 5.45; N, 16.79.

Synthesis of 2-amino-4-(1-methyl-1*H*-indol-3-yl)pyrimidin-5-ylamine (2a): To a solution of 4-(1-methyl-1*H*-indol-3-yl)-5-nitropyrimidin-2-ylamine (**7a**) (3.71 mmol) in methanol (20 mL) was added 10% Pd on charcoal (0.1 g) and the reaction mixture was placed in hydrogenation assembly at ambient temperature for 2 h at a hydrogen pressure of 4 bar. After completion of the reaction as evident by TLC analysis, the reaction mixture was filtered over celite and the filtrate was concentrated and triturated with diethyl ether to furnish 4-(1-methyl-1*H*-indol-3-yl)pyrimidine-2,5-diamine (**2a**) as a light-yellow solid.

Yield = 93%; yellow solid; mp 158–160 °C; R_f 0.10 (3:7 EtOAc:hexane); IR (KBr) ν_{max}: 3020, 2975 cm^{-1}; 1H NMR (300 MHz, DMSO-d_6) δ 8.68 (1H, d, *J* = 7.83 Hz, ArH), 8.16 (1H, s, ArH), 7.88 (1H, s, ArH), 7.48 (1H, d, *J* = 8.04 Hz, ArH), 7.26-7.21 (1H, m, ArH), 7.14 (1H, t, *J* = 7.11 Hz, ArH), 5.67 (2H, s, NH_2), 4.28 (2H, s, NH_2), 3.86 (3H, s, CH_3); ^{13}C NMR (75 MHz, DMSO-d_6) δ 157.5, 149.2, 146.4, 136.4, 132.5, 129.4, 126.8, 123.5, 122.1, 120.0, 111.1, 109.6, 32.9; ESMS *m/z*: 240.1 [M$^+$ + 1]; Anal. calcd for $C_{13}H_{13}N_5$: C, 65.25; H, 5.48; N, 29.27; found: C, 65.20; H, 5.49; N, 29.31.

Compounds **2b**, **18a** and **18b** were synthesized analogously to compound **2a** starting from **7b**, **17a** and **17b**, respectively.

4-(5-Methoxy-1-methyl-1*H*-indol-3-yl)pyrimidine-2,5-diamine (2b): Yield = 95%; yellow solid; mp 216–218 °C; R_f 0.02 (3:7 EtOAc:hexane); IR (KBr) ν_{max}: 3316, 3019, 1216 cm^{-1}; ^1H NMR (300 MHz, DMSO-d_6) δ 8.17 (1H, d, J = 2.31 Hz, ArH), 8.10 (1H, s, ArH), 7.85 (1H, s, ArH), 7.37 (1H, d, J = 8.76 Hz, ArH), 6.87 (1H, dd, J_1 = 2.61 Hz, J_2 = 8.85 Hz, ArH), 5.65 (2H, s, NH_2), 4.24 (2H, s, NH_2), 3.82 (6H, s, OCH_3, CH_3); ^{13}C NMR (75 MHz, DMSO-d_6) δ 157.6, 154.4, 149.6, 146.4, 133.0, 131.9, 129.3, 127.4, 111.9, 110.7, 110.4, 105.5, 55.5, 33.1; ESMS *m/z*: 270.0 [M^+ + 1]; Anal. calcd for $C_{14}H_{15}N_5O$: C, 62.44; H, 5.61; N, 26.01; found: C, 62.40; H, 5.63; N, 26.04.

4-(3,4-Dimethoxyphenyl)pyrimidine-2,5-diamine (18a): Yield = 92%; yellow solid; mp 198–200 °C; R_f 0.15 (3:7 EtOAc:hexane); IR (KBr) ν_{max}: 3153, 2367, 1633 cm^{-1}; ^1H NMR (300 MHz, $CDCl_3$) δ 7.91 (1H, s, ArH), 7.40 (2H, d, J_1 = 4.9 Hz, ArH), 7.03 (1H, d, J = 8.82 ArH), 5.73 (2H, s, NH_2), 4.29 (2H, s, NH_2), 3.80 (3H, s, OCH_3), 3.79 (3H, s, OCH_3); ^{13}C NMR (75 MHz, DMSO-d_6) δ 157.5, 149.9, 149.3, 148.4, 147.5, 130.4, 129.9, 121.0, 111.8, 111.3, 55.5, 55.4; ESMS *m/z*: 247.1 [M^+ + 1]; Anal. calcd for $C_{12}H_{14}N_4O_2$: C, 58.53; H, 5.73; N, 22.75; found: C, 58.50; H, 5.75; N, 22.79.

4-(3,4,5-Trimethoxyphenyl)pyrimidine-2,5-diamine (18b): Yield = 96%; yellow solid; mp 216–218 °C; R_f 0.10 (3:7 EtOAc:hexane); IR (KBr) ν_{max}: 3197, 1624 cm^{-1}; ^1H NMR (300 MHz, DMSO-d_6) δ 7.93 (1H, s, ArH), 7.08 (2H, s, ArH), 5.74 (2H, s, NH_2), 4.38 (2H, s, NH_2), 3.82 (6H, s, 2 X OCH_3), 3.71 (3H, s, OCH_3); ^{13}C NMR (75 MHz, DMSO-d_6) δ 157.5, 152.7, 149.5, 147.8, 137.9, 132.9, 130.6, 105.7, 60.1, 55.9; ESMS *m/z*: 277.1 [M^+ + 1]; Anal. calcd for $C_{13}H_{16}N_4O_3$: C, 56.51; H, 5.84; N, 20.28; found: C, 56.55; H, 5.80; N, 20.30.

General Procedure for the synthesis of pyrimido-β-carbolines (9a–e and 12a–d): In a similar manner as described in a procedure from reference **[57]**.

6-(4-Chlorophenyl)-7-methyl-7*H*-pyrimido[4',5':5,6]pyrido[3,4-*b*]indol-2-amine (9a): Yield = 73%; yellow solid; mp >250 °C; R_f 0.20 (3:7 EtOAc:hexane); IR (KBr) ν_{max}: 3020, 2850 cm^{-1}; ^1H NMR (300 MHz, DMSO-d_6) δ 9.11 (1H, s, ArH), 8.89 (1H, d, *J* = 7.68 Hz, ArH), 7.77-7.69 (3H, m, ArH), 7.65-7.62 (3H, m, ArH), 7.44 (1H, t, *J* = 7.26 Hz, ArH), 7.13(2H, s, NH_2), 3.54 (3H, s, CH_3); ^{13}C NMR (75 MHz, DMSO-d_6) δ 161.3, 161.2, 145.3, 142.3, 140.9, 138.1, 135.7, 133.4, 131.4, 128.8, 128.1, 127.0, 123.6, 121.0, 120.9, 118.6, 110.7, 33.1; ESMS *m/z*: 360.1 [M^+ + 1]; Anal. calcd for $C_{20}H_{14}ClN_5$: C, 66.76; H, 3.92; N, 19.46; found: C, 66.78; H, 3.95; N, 19.47.

6-(4-Ethoxyphenyl)-7-methyl-7*H*-pyrimido[4',5':5,6]pyrido[3,4-*b*]indol-2-amine (9b): Yield = 82%; yellow solid; mp >250 °C; R_f 0.16 (3:7 EtOAc:hexane); IR (KBr) ν_{max}: 3010, 1590 cm^{-1}; ^1H NMR (300 MHz, DMSO-d_6) δ 9.10 (1H, s, ArH), 8.89 (1H, d, *J* = 7.74 Hz, ArH), 7.76 (1H, d, *J* = 8.34 Hz, ArH), 7.66-7.59 (3H, m, ArH), 7.44 (1H, t, *J* = 7.20 Hz, ArH), 7.12 (2H, s, NH_2), 7.08 (2H, d, *J* = 5.04 Hz, ArH), 4.14 (2H, q, *J* = 6.90 Hz, ArH), 3.56 (3H, s, CH_3), 1.40 (3H, t, *J* = 6.90 Hz, CH_3); ^{13}C NMR (75 MHz, DMSO-d_6) δ 161.1, 158.8, 145.3, 143.7, 140.9, 136.0, 131.4, 130.8, 128.9, 126.9, 123.7, 121.2, 120.9, 118.5, 113.9, 110.8, 63.2, 33.0, 14.7; ESMS *m/z*: 370.1 [M^+ + 1]; Anal. calcd for $C_{22}H_{19}N_5O$: C, 71.53; H, 5.18; N, 18.96; found: C, 71.50; H, 5.19; N, 18.98.

6-(4-Methoxyphenyl)-7-methyl-7*H*-pyrimido[4',5':5,6]pyrido[3,4-*b*]indol-2-ylamine (9c): Yield = 78%; yellow solid; mp >250 °C; R_f 0.16 (3:7 EtOAc:hexane); IR (KBr) ν_{max}: 3010, 2851 cm^{-1}; ^1H NMR (300 MHz, DMSO-d_6) δ 9.10 (1H, s, ArH), 8.89 (1H, d, *J* = 7.83 Hz, ArH), 7.74 (1H, d, *J* = 8.34 Hz, ArH), 7.65-7.60 (3H, m, ArH), 7.43 (1H, t, *J* = 7.20 Hz, ArH), 7.12 (2H, d, *J* = 8.67 Hz, ArH), 7.07(2H, s, NH_2), 3.87 (3H, s, OCH_3), 3.55 (3H, s, CH_3); ^{13}C NMR (75 MHz, DMSO-d_6) δ 161.1, 159.5, 156.6, 145.3, 143.7, 141.0, 136.1, 131.5, 130.8, 130.3,

128.9, 126.9, 123.7, 121.2, 120.9, 118.5, 114.4, 113.5, 110.8, 55.2, 33.0; ESMS *m/z*: 356.1 [M^+ + 1]; Anal. calcd for $C_{21}H_{17}N_5O$: C, 70.97; H, 4.82; N, 19.71; found: C, 70.92; H, 4.83; N, 19.74.

6-(4-Bromophenyl)-7-methyl-7*H*-pyrimido[4',5':5,6]pyrido[3,4-*b*]indol-2-ylamine (9d): Yield = 85%; yellow solid; mp >250 °C; R_f 0.21 (3:7 EtOAc:hexane); IR (KBr) ν_{max}: 3010, 2855 cm^{-1}; ^1H NMR (300 MHz, DMSO-d_6) δ 9.13 (1H, s, ArH), 8.89 (1H, d, *J* = 7.56 Hz, ArH), 8.01 (1H, d, *J* = 1.71 Hz, ArH), 7.85-7.62 (5H, m, ArH), 7.45 (1H, t, *J* = 7.41 Hz, ArH), 7.16 (2H, s, NH_2), 3.58 (3H, s, CH_3); ^{13}C NMR (75 MHz, DMSO-d_6) δ 161.4, 161.2, 145.3, 140.9, 140.9, 139.7, 135.7, 131.4, 130.9, 130.2, 129.9, 128.7, 123.7, 121.0, 118.8, 110.8, 33.1; ESMS *m/z*: 404.1 [M^+ + 1]; Anal. calcd for $C_{20}H_{14}BrN_5$: C, 59.42; H, 3.49; N, 17.32; found: C, 59.40; H, 3.46; N, 17.35.

6-(3,4-Dichlorophenyl)-7-methyl-7*H*-pyrimido[4',5':5,6]pyrido[3,4-*b*]indol-2-amine (9e): Yield = 80%; yellow solid; mp >250 °C; R_f 0.24 (3:7 EtOAc:hexane); IR (KBr) ν_{max}: 3050, 1586 cm^{-1}; ^1H NMR (300 MHz, DMSO-d_6) δ 9.11 (1H, s, ArH), 8.88 (1H, d, *J* = 7.62 Hz, ArH), 7.83-7.61 (3H, m, ArH), 7.67-7.61 (3H, m, ArH), 7.43 (1H, t, *J* = 7.32 Hz, ArH), 7.14 (2H, s, NH_2), 3.53 (3H, s, CH_3); ^{13}C NMR (75 MHz, DMSO-d_6) δ 161.3, 161.2, 145.3, 142.4, 140.9, 138.5, 135.7, 131.7, 131.0, 128.8, 127.0, 123.7, 122.1, 121.1, 118.7, 110.8, 33.2; ESMS *m/z*: 394.1 [M^+ + 1]; Anal. calcd for $C_{20}H_{13}Cl_2N_5$: C, 60.93; H, 3.32; N, 17.76; found: C, 60.90; H, 3.30; N, 17.78.

10-Methoxy-7-methyl-6-(4-chlorophenyl)-6,7-dihydro-5*H*-pyrimido[4',5':5,6]pyrido[3,4-*b*]indol-2-amine (12a): Yield = 90% yellow solid; mp 244–246 °C; R_f 0.30 (5:5 EtOAc:hexane); IR (KBr) ν_{max}: 3194, 1624, 1455 cm^{-1}; ^1H NMR (300 MHz, DMSO-d_6) δ 7.95 (1H, s, ArH), 7.59 (1H, s, ArH), 7.45 (1H, d, *J*=8.91 Hz, ArH), 7.17 (2H, d, *J* = 8.43 Hz, ArH), 7.03 (1H, d, *J* =

8.91 Hz, ArH), 6.85 (2H, d, J = 8.43 Hz, ArH), 5.94 (2H, s, NH_2), 5.66 (1H, s, CH), 5.66 (1H, s, NH), 3.90 (3H, s, OCH_3), 3.74 (3H, s, CH_3); ^{13}C NMR (50 MHz, DMSO-d_6) δ 158.6, 152.3, 150.2, 149.2, 142.1, 132.7, 131.1, 130.8, 129.5, 128.1, 127.9, 125.9, 121.1, 113.2, 109.7, 107.8, 56.8, 55.7, 33.1; ESMS *m/z*: 392.1 [M^+ + 1]; Anal. calcd for $C_{21}H_{18}ClN_5O$: C, 64.37; H, 4.63; N, 17.87; found: C, 64.35; H, 4.60; N, 17.85.

10-Methoxy-7-methyl-6-(4-fluorophenyl)-6,7-dihydro-5*H*-pyrimido[4',5':5,6]pyrido[3,4-*b*]indol-2-amine (12b): Yield = 91%; yellow solid; mp 238–240 °C; R_f 0.025 (5:5 EtOAc:hexane); IR (KBr) ν_{max}: 3464, 2925, 1212 cm^{-1}; ^{1}H NMR (300 MHz, DMSO-d_6) δ 7.94 (1H, s, ArH), 7.59 (1H, s, ArH), 7.44 (1H, d, J = 8.91Hz, ArH), 7.03 (1H, d, J = 8.88 Hz, ArH), 6.95-6.83 (4H, m, ArH), 5.94 (2H, s, NH_2), 5.64 (1H, s,CH), 5.64 (1H, s, NH,), 3.89 (3H, s, OCH_3), 3.74 (3H, s, CH_3); ^{13}C NMR (50 MHz, DMSO-d_6) δ 158.6, 152.3, 150.3, 149.2, 139.3, 132.8, 131.1, 129.6, 129.4, 128.2, 125.9, 121.5, 114.9, 114.4, 113.3, 109.7, 107.9, 56.8, 55.6, 33.1; ESMS *m/z*: 376.2 (M^++1); Anal. calcd for $C_{21}H_{18}FN_5O$: C, 67.19; H, 4.83; N, 18.66; found: C, 67.2; H, 4.82; N, 18.64.

10-Methoxy-7-methyl-6-(4-methoxyphenyl)-6,7-dihydro-5*H*-pyrimido[4',5':5,6]pyrido[3,4-*b*]indol-2-amine (12c): Yield = 87%; yellow solid; mp 214–216 °C; R_f 0.05 (5:5 EtOAc:hexane); IR (KBr) ν_{max}: 3458, 3019, 1216 cm^{-1}; ^{1}H NMR (300 MHz, DMSO-d_6) δ 7.93 (1H, s, ArH), 7.59 (1H, s, ArH), 7.42 (1H, d, J = 8.88 Hz, ArH), 7.02 (1H, d, J = 8.91 Hz, ArH), 6.75 (2H, d, J = 8.64 Hz, ArH), 6.64 (2H, d, J = 8.7 Hz, ArH), 5.89 (2H, s, NH_2), 5.61 (1H, s, CH), 5.61 (1H, s, NH), 3.89 (3H, s, OCH_3), 3.72 (3H, s, OCH_3) 3.60 (3H, s, CH_3); ^{13}C NMR (50 MHz, DMSO-d_6) δ 158.52, 157.5, 152.2, 150.2, 149.1, 135.0, 132.8, 130.9, 128.7, 128.6, 126.0, 122.2, 113.5, 113.2, 109.4, 108.0, 56.8, 55.7, 54.8, 33.1; ESMS *m/z*: 388.2 [M^+ + 1]; Anal. calcd for $C_{22}H_{21}N_5O_2$: C, 68.20; H, 5.46; N, 18.08; found: C, 68.21; H, 5.47; N, 18.06.

10-Methoxy-7-methyl-6-(3,4-dimethoxyphenyl)-6,7-dihydro-5*H*-pyrimido[4',5':5,6]pyrido[3,4-*b*]indol-2-amine (12d): Yield = 88%; yellow solid; mp >250 °C; R_f 0.02 (5:5 EtOAc:hexane); IR (KBr) ν_{max}: 3098, 2363, 16636 cm^{-1}; ^{1}H NMR (300 MHz, DMSO-d_6) δ 7.92 (1H, s, ArH), 7.66 (1H, s, ArH), 7.43 (1H, d, *J* = 8.94 Hz, ArH), 7.03 (1H, d, *J* = 8.85 Hz, ArH), 6.94 (1H, s, ArH), 6.52 (1H, d, *J* = 8.4 Hz, ArH), 5.92 (1H, s, ArH), 5.89 (2H, s, NH_2), 5.56 (1H, s, CH), 5.56 (1H, s, NH), 3.90 (3H, s, OCH_3), 3.75 (3H, s, OCH_3) 3.64 (3H, s, OCH_3) 3.59 (3H, s, CH_3); ^{13}C NMR (50 MHz, DMSO-d_6) δ 158.5, 152.1, 150.2, 149.2, 148.4, 147.1, 135.4, 132.7, 130.8, 128.6, 126.0, 121.9, 119.2, 113.4, 112.1, 110.6, 109.3, 107.8, 56.8, 55.9, 55.3, 55.1, 33.0; ESMS *m/z*: 418.2 [M$^+$ + 1]; Anal. calcd for $C_{23}H_{23}N_5O_3$: C, 66.17; H, 5.55; N, 16.78; found: C, 66.19; H, 5.57; N, 16.76.

Synthesis of *N*,*N*-dimethyl-4-(1-methyl-1*H*-indol-3-yl)-5-nitropyrimidin-2-amine (8): To a stirred solution of dimethylguanidine sulfate salt (17.34 mmol) and sodium methoxide (17.34 mmol) in methanol (20 mL) was added (*E*)-3-(dimethylamino)-1-(1-methyl-1*H*-indol-3-yl)-2-(nitromethyl)prop-2-en-1-one (**6a**) (5.78 mmol) and the reaction mixture was heated under reflux for 16 h. After completion of the reaction as evident by TLC analysis, ethanol was distilled off, water (20 mL) was added, and the title compound was extracted with ethyl acetate (3 x 15 mL). The organic layer was washed with brine, dried over Na_2SO_4 and concentrated in vacuo. The obtained residue was triturated with diethyl ether to furnish pure compound **8** as a light-yellow solid.

Yield = 76%; yellow solid; mp 132–134 °C; R_f 0.62 (3:7 EtOAc:hexane); IR (KBr) ν_{max}: 2990, 2851 cm^{-1}; ^{1}H NMR (300 MHz, $CDCl_3$) δ 8.96 (1H, s, ArH), 8.08 (1H, d, *J* = 7.59 Hz, ArH), 7.77 (1H, s, ArH), 7.38-7.22 (3H, m, ArH), 3.85 (3H, s, CH_3), 3.38 (3H, s, CH_3), 3.35 (3H, s, CH_3); ^{13}C NMR (75 MHz, $CDCl_3$) δ 160.9, 156.4, 137.2, 134.4, 132.9, 126.8, 122.8, 121.8,

121.6, 110.6, 109.9, 37.9, 33.4; ESMS *m/z*: 298.1 [M^+ + 1]; Anal. calcd for $C_{15}H_{15}N_5O_2$: C, 60.60; H, 5.09; N, 23.56; found: C, 60.64; H, 5.05; N, 23.59.

Compounds **2c** and **18c** were synthesized analogously to compound **2a** starting from **8** and **17c**, respectively.

***N*^{2},*N*^{2}-Dimethyl-4-(1-methyl-1*H*-indol-3-yl)pyrimidine-2,5-diamine (2c):**Yield = 95%; yellow solid; mp 164–166 °C; R_f 0.40 (3:7 EtOAc:hexane); IR (KBr) ν_{max}: 3060, 2850 cm^{-1}; ^{1}H NMR (300 MHz, $CDCl_3$) δ 8.49 (1H, d, *J* = 7.71 Hz, ArH), 7.97 (1H, s, ArH), 7.88 (1H, s, ArH), 7.38-7.22 (3H, m, ArH), 3.85 (3H, s, CH_3), 3.23 (6H, s, 2 X CH_3); ^{13}C NMR (75 MHz, $CDCl_3$) δ 158.6, 150.4, 146.5, 137.2, 131.2, 126.9, 122.8, 122.7, 120.7, 112.4, 109.4, 37.8, 33.2; ESMS *m/z*: 268.2 [M^+ + 1]; Anal. calcd for $C_{15}H_{17}N_5$: C, 67.39; H, 6.41; N, 26.20; found: C, 67.44; H, 6.40; N, 26.16

***N*^{2},*N*^{2}-Dimethyl-4-(3,4,5-trimethoxyphenyl)pyrimidine-2,5-diamine (18c):** Yield = 92%; yellow solid; mp 126–128 °C; R_f 0.46 (3:7 EtOAc:hexane); IR (KBr) ν_{max}: 3320, 2930, 1596 cm^{-1}; ^{1}H NMR (300 MHz, DMSO-d_6) δ 8.06 (1H, s, ArH), 7.18 (2H, s, ArH), 4.45 (2H, s, NH_2), 3.82 (6H, s, 2 x OCH_3), 3.72 (3H, s, OCH_3), 3.05 (6H, s, 2 x CH_3); ^{13}C NMR (75 MHz, DMSO-d_6) δ 156.9, 152.7, 148.9, 147.7, 138.0, 133.2, 129.9, 105.7, 60.0, 55.8, 37.2; ESMS *m/z*: 305.3 [M^+ + 1]; Anal. calcd for $C_{15}H_{20}N_4O_3$: C, 59.20; H, 6.62; N, 18.41; found: C, 57.20; H, 6.65; N, 18.45.

General Procedure for the synthesis of pyrimido-β-carbolines (9f–k and 20a–l): In a similar manner as described in a procedure from reference [57].

6-(4-Bromophenyl)-*N*,*N*,7-trimethyl-7*H*-pyrimido[4',5':5,6]pyrido[3,4-*b*]indol-2-amine (9f): Yield = 72%; yellow solid; mp 230–232 °C; R_f 0.70 (3:7 EtOAc:hexane); IR (KBr) ν_{max}: 3010, 2851 cm^{-1}; ^{1}H NMR (300 MHz, $CDCl_3$) δ 9.26 (1H, s, ArH), 8.93 (1H, d, *J* = 7.71 Hz, ArH),

7.70 (2H, d, J = 8.31 Hz, ArH), 7.65-7.44 (5H, m, ArH), 3.56 (3H, s, CH_3), 3.47 (6H, s, 2 x CH_3); ^{13}C NMR (75 MHz, $CDCl_3$) δ 161.0, 159.9, 145.7, 142.6, 141.5, 138.7, 136.4, 131.7, 131.1, 128.9, 127.1, 124.5, 123.2, 122.1, 121.4, 120.2, 109.8, 37.7, 33.3; ESMS m/z: 432.2 [M^+ + 1]; Anal. calcd for $C_{22}H_{18}BrN_5$: C, 61.12; H, 4.20; N, 16.20; found: C, 61.10; H, 4.24; N, 16.23.

***N,N*,7-Trimethyl-6-(4-nitrophenyl)-7*H*-pyrimido[4',5':5,6]pyrido[3,4-*b*]indol-2-amine (9g):** Yield = 76%; yellow solid; mp 234–236 °C; R_f 0.62 (3:7 EtOAc:hexane); IR (KBr) ν_{max}: 3000, 2910 cm^{-1}; ^{1}H NMR (300 MHz, $CDCl_3$) δ 9.26 (1H, s, ArH), 8.95 (1H, d, J = 7.77 Hz, ArH), 8.43 (2H, d, J = 8.58 Hz, ArH), 7.90 (2H, d, J = 8.58 Hz, ArH), 7.67-7.63 (1H, m, ArH), 7.54-7.47 (2H, m, ArH), 3.56 (3H, s, CH_3), 3.48 (6H, s, 2 x CH_3); ^{13}C NMR (75 MHz, $CDCl_3$) δ 161.3, 160.0, 148.0, 146.3, 145.7, 141.6, 130.6, 129.1, 127.5, 124.6, 123.8, 122.2, 121.7, 116.0, 109.9, 37.8, 33.7, 29.8; ESMS m/z: 399.2 [M^+ + 1]; Anal. calcd for $C_{22}H_{18}N_6O_2$: C, 66.32; H, 4.55; N, 21.09; found: C, 66.35; H, 4.50; N, 21.05.

6-(4-Methoxyphenyl)-*N,N*,7-trimethyl-7*H*-pyrimido[4',5':5,6]pyrido[3,4-*b*]indol-2-amine (9h): Yield = 79%; yellow solid; mp 200–202 °C; R_f 0.22 (3:7 EtOAc:hexane); IR (KBr) ν_{max}: 3060, 2850, 1590 cm^{-1}; ^{1}H NMR (300 MHz, $CDCl_3$) δ 9.27 (1H, s, ArH), 8.94 (1H, d, J = 7.77 Hz, ArH), 7.60 (3H, d, J = 8.49 Hz, ArH), 7.51-7.43 (2H, m, ArH), 7.08 (2H, d, J = 8.58 Hz, ArH), 3.91 (3H, s, OCH_3), 3.56 (3H, s, CH_3), 3.46 (6H, s, 2 x CH_3); ^{13}C NMR (75 MHz, $CDCl_3$) δ 160.8, 160.1, 159.8, 145.7, 144.0, 141.4, 136.7, 132.2, 130.7, 128.9, 126.8, 124.4, 122.1, 121.4, 119.9, 113.9, 109.8, 55.5, 37.7, 33.1; ESMS m/z: 384.1 [M^+ + 1]; Anal. calcd for $C_{23}H_{21}N_5O$: C, 72.04; H, 5.52; N, 18.26; found: C, 72.00; H, 5.50; N, 18.28.

6-(3,4-Dimethoxyphenyl)-*N,N*,7-trimethyl-7*H*-pyrimido[4',5':5,6]pyrido[3,4-*b*]indol-2-amine (9i): Yield = 81%; yellow solid; mp 214–216 °C; R_f 0.12 (3:7 EtOAc:hexane); IR (KBr) ν_{max}: 3010, 1590 cm^{-1}; ^{1}H NMR (300 MHz, $CDCl_3$) δ 9.28 (1H, s, ArH), 8.95 (1H, d, J =

7.89 Hz, ArH), 7.64-7.59 (1H, m, ArH), 7.53-7.44 (2H, m, ArH), 7.20 (2H, d, *J* = 7.38 Hz, ArH), 7.03 (1H, d, *J* = 8.10 Hz, ArH), 3.98 (3H, s, OCH_3), 3.95 (3H, s, OCH_3), 3.58 (3H, s, CH_3), 3.47 (6H, s, 2 x CH_3); ^{13}C NMR (75 MHz, $CDCl_3$) δ 160.9, 159.9, 149.6, 149.0, 145.8, 143.9, 141.5, 136.7, 132.3, 128.8, 126.9, 124.5, 122.2, 121.3, 119.9, 112.5, 111.1, 109.8, 56.1, 37.7, 33.0; ESMS *m/z*: 414.2 [M^+ + 1]; Anal. calcd for $C_{24}H_{23}N_5O_2$: C, 69.72; H, 5.61; N, 16.94; found: C, 69.70; H, 5.54; N, 16.97.

6-(4-Chlorophenyl)-*N*,*N*,7-trimethyl-7*H*-pyrimido[4',5':5,6]pyrido[3,4-*b*]indol-2-amine (9j): Yield = 86%; yellow solid; mp 224–226 °C; R_f 0.46 (3:7 EtOAc:hexane); IR (KBr) ν_{max}: 3063, 2851, 1690 cm^{-1}; ^{1}H NMR (300 MHz, $CDCl_3$) δ 9.26 (1H, s, ArH), 8.93 (1H, d, *J* = 7.77 Hz, ArH), 7.64-7.61 (3H, m, ArH), 7.55-7.44 (4H, m, ArH), 3.55 (3H, s, CH_3), 3.47 (6H, s, 2 x CH_3); ^{13}C NMR (75 MHz, $CDCl_3$) δ 160.9, 159.8, 145.6, 142.5, 141.4, 138.2, 136.4, 134.9, 130.8, 128.8, 128.7, 127.0, 124.4, 122.0, 121.4, 120.1, 109.7, 37.6, 33.2; ESMS *m/z*: 388.2 [M^+ + 1]; Anal. calcd for $C_{22}H_{18}ClN_5$: C, 68.13; H, 4.68; N, 18.06; found: C, 68.13; H, 4.64; N, 18.00.

6-(2-Chlorophenyl)-*N*,*N*,7-trimethyl-7*H*-pyrimido[4',5':5,6]pyrido[3,4-*b*]indol-2-amine (9k): Yield = 78%; yellow solid; mp 198–200 °C; R_f 0.45 (3:7 EtOAc:hexane); IR (KBr) ν_{max}: 3063, 2851, 1690 cm^{-1}; ^{1}H NMR (300 MHz, $CDCl_3$) δ 9.29 (1H, s, ArH), 8.95 (1H, d, *J* = 7.74 Hz, ArH), 7.64-7.44 (7H, m, ArH), 3.52 (3H, s, CH_3), 3.48 (6H, s, 2 x CH_3); ^{13}C NMR (75 MHz, $CDCl_3$) δ 161.2, 160.1, 146.1, 141.3, 141.2, 138.7, 136.7, 134.2, 131.6, 130.5, 129.7, 128.8, 127.4, 127.0, 124.5, 122.1, 121.4, 119.6, 109.8, 37.8, 31.0; ESMS *m/z*: 388.2 [M^+ + 1]; Anal. calcd for $C_{22}H_{18}ClN_5$: C, 68.13; H, 4.68; N, 18.06; found: C, 68.15; H, 4.67; N, 18.02.

6-(4-Fluorophenyl)-8,9-dimethoxypyrimido[5,4-*c*]isoquinolin-2-amine (20a): Yield = 86%; yellow solid; mp 246–248 °C; R_f 0.05 (3:7 EtOAc:hexane); IR (KBr) ν_{max}: 2889, 2368, 1651 cm^{-1}; ^{1}H NMR (400 MHz, DMSO-d_6) δ 9.04 (1H, s, ArH), 8.25 (1H, s, ArH), 7.83-7.78

(2H, m, ArH), 7.43-7.37 (2H, m, ArH), 7.32 (1H, s, ArH), 7.11 (2H, s, NH_2), 4.02 (3H, s, OCH_3), 3.82 (3H, s, OCH_3); ^{13}C NMR (100 MHz, DMSO-d_6) δ 161.9, 161.7, 154.0, 152.7, 152.3, 145.8, 136.2, 132.0, 132.0, 130.0, 127.4, 124.3, 115.8, 115.5, 108.0, 103.8, 56.4, 56.2, 55.2; ESMS *m/z*: 351.3 [M^+ + 1]; Anal. calcd for $C_{19}H_{15}FN_4O_2$: C, 65.14; H, 4.32; F, 5.42; N, 15.99; found: C, 65.12; H, 4.30; F, 5.40; N, 15.98.

6-(4-Bromophenyl)-8,9-dimethoxypyrimido[5,4-*c*]isoquinolin-2-amine (20b): Yield = 82%; yellow solid; mp >250 °C; R_f 0.06 (3:7 EtOAc:hexane); IR (KBr) ν_{max}: 3182, 2850, 1645 cm^{-1}; ^{1}H NMR (300 MHz, DMSO-d_6) δ 9.04 (1H, s, ArH), 8.23 (1H, s, ArH), 7.79-7.70 (4H, m, ArH), 7.31 (1H, s, ArH), 7.10 (2H, s, NH_2) 4.00 (3H, s, OCH_3), 3.82 (3H, s, OCH_3); ^{13}C NMR (100 MHz, DMSO-d_6) δ 161.1, 160.9, 152.9, 151.9, 151.4, 144.9, 138.1, 131.2, 130.9, 129.2, 126.6, 123.3, 121.6, 107.1, 102.9, 55.6, 55.4; ESMS *m/z*: 411.1 [M^+ + 1]; Anal. calcd for $C_{19}H_{15}BrN_4O_2$: C, 55.49; H, 3.68; N, 13.62; found: C, 55.47; H, 3.66; N, 13.60.

6-(4-Chlorophenyl)-8,9-dimethoxypyrimido[5,4-c]isoquinolin-2-amine (20c): Yield = 80%; yellow solid; mp >250 °C; R_f 0.05 (3:7 EtOAc:hexane); IR (KBr) ν_{max}: 3166, 2370, 1648 cm^{-1}; ^{1}H NMR (300 MHz, DMSO-d_6) δ 9.04 (1H, s, ArH), 8.24 (1H, s, ArH), 7.78 (2H, d, J = 8.31, ArH), 7.63 (2H, d, J = 8.22, ArH), 7.31 (1H, s, ArH), 7.12 (2H, s, NH_2), 4.00 (3H, s, OCH_3), 3.82 (3H, s, OCH_3); ^{13}C NMR (100 MHz, $CDCl_3$) δ 161.2, 161.0, 153.0, 152.0, 151.5, 145.0, 137.8, 133.2, 131.0, 129.2, 128.1, 126.6, 123.4, 114.2, 107.1, 103.0, 55.6, 55.4, 54.4; ESMS *m/z*: 367.3 [M^+ + 1]; Anal. calcd for $C_{19}H_{15}ClN_4O_2$: C, 62.21; H, 4.12; N, 15.27; ; found: C, 62.20; H, 4.10; N, 15.28.

7,8,9-Trimethoxy-6-(4-methoxyphenyl)pyrimido[5,4-*c*]isoquinolin-2-amine (20d): Yield = 89%; yellow solid; mp >250 °C; R_f 0.21 (3:7 EtOAc:hexane); IR (KBr) ν_{max}: 3021, 1598 cm^{-1}; ^{1}H NMR (300 MHz, DMSO-d_6) δ 9.01 (1H, s, ArH), 8.21 (1H, s, ArH), 7.60 (2H, d, J = 8.31 Hz,

ArH), 7.40 (2H, d, J = 8.31 Hz, ArH), 7.17 (2H, s, NH_2), 4.05 (3H, s, OCH_3), 3.84 (3H, s, OCH_3), 3.31 (3H, s, OCH_3), 3.28 (3H, s, OCH_3); ^{13}C NMR (75 MHz, DMSO-d_6) δ 161.4, 161.2, 158.5, 155.9, 153.2, 150.4, 144.7, 135.7, 129.7, 129.3, 118.5, 112.2, 99.6, 60.9, 60.8, 56.1, 55.1; ESMS *m/z*: 393.2 [M^+ + 1]; Anal. calcd for $C_{21}H_{20}N_4O_4$: C, 64.28; H, 5.14; N, 14.28; found: C, 64.30; H, 5.12; N, 14.25.

7,8,9-Trimethoxy-6-(4-chlorophenyl)pyrimido[5,4-*c*]isoquinolin-2-amine (20e): Yield = 83%; yellow solid; mp >250 °C; R_f 0.23 (3:7 EtOAc:hexane); IR (KBr) ν_{max}: 3063, 1626 cm^{-1}; ^{1}H NMR (300 MHz, DMSO-d_6) δ 9.02 (1H, s, ArH), 8.22 (1H, s, ArH), 7.47 (4H, s, ArH), 7.20 (2H, s, NH_2), 4.05 (3H, s, OCH_3), 3.84 (3H, s, OCH_3), 3.27 (3H, s, OCH_3); ^{13}C NMR (75 MHz, DMSO-d_6) δ 162.0, 161.8, 156.6, 152.5, 150.5, 145.3, 145.1, 142.7, 132.1, 130.6, 129.7, 129.3, 127.3, 118.8, 100.1, 61.2, 61.2, 56.6; ESMS *m/z*: 397.1 [M^+ + 1]; Anal. calcd for $C_{20}H_{17}ClN_4O_3$: C, 60.53; H, 4.32; N, 14.12; found: C, 60.50; H, 4.35; N, 14.17.

7,8,9-Trimethoxy-6-(4-fluorophenyl)pyrimido[5,4-*c*]isoquinolin-2-amine (20f): Yield = 85%; yellow solid; mp >250 °C; R_f 0.22 (3:7 EtOAc:hexane); IR (KBr) ν_{max}: 3022, 1621 cm^{-1}; ^{1}H NMR (300 MHz, $CDCl_3$) δ 9.01 (1H, s, ArH), 8.21 (1H, s, ArH), 7.50-7.46 (2H, m, ArH), 7.26-7.16 (2H, m, ArH), 7.16 (2H, s, NH_2), 4.04 (3H, s, OCH_3), 3.84 (3H, s, OCH_3), 3.25 (3H, s, OCH_3); ^{13}C NMR (75 MHz, $CDCl_3$) δ 161.5, 161.3, 156.1, 152.4, 150.1, 144.8, 144.7, 139.8, 139.7, 130.4, 130.2, 129.2, 128.8, 118.5, 113.9, 113.4, 99.7, 60.76, 56.1; ESMS *m/z*: 381.2 [M^+ + 1]; Anal. calcd for $C_{20}H_{17}FN_4O_3$: C, 63.15; H, 4.50; N, 14.73; found: C, 63.12; H, 4.53; N, 14.75.

7,8,9-Trimethoxy-6-(4-bromophenyl)pyrimido[5,4-*c*]isoquinolin-2-amine (20g): Yield = 87%; yellow solid; mp >250 °C; R_f 0.19 (3:7 EtOAc:hexane); IR (KBr) ν_{max}: 3021, 1608 cm^{-1}; ^{1}H NMR (300 MHz, DMSO-d_6) δ 9.07 (1H, s, ArH), 8.27 (1H, s, ArH), 7.66 (2H, d, J = 8.34 Hz,

ArH), 7.46 (2H, d, J = 8.34 Hz, ArH), 7.23 (2H, s, NH_2), 4.11 (3H, s, OCH_3), 3.90 (3H, s, OCH_3), 3.34 (3H, s, OCH_3); ^{13}C NMR (75 MHz, DMSO-d_6) δ 161.6, 161.3, 156.2, 152.1, 150.0, 144.9, 144.6, 142.6, 130.4, 129.8, 129.2, 128.8, 120.2, 118.3, 99.6, 60.8, 60.7, 56.1; ESMS *m/z*: 441.1 [M^+ + 1]; Anal. calcd for $C_{20}H_{17}BrN_4O_3$: C, 54.44; H, 3.88; N, 12.70; found: C, 54.40; H, 3.89; N, 12.73.

***N,N*-Dimethyl-*N*-(7,8,9-trimethoxy-6-(4-nitrophenyl)pyrimido[5,4-*c*]isoquinolin-2-yl)amine (20h):** Yield = 81%; yellow solid; mp 186–188 °C; R_f 0.62 (3:7 EtOAc:hexane); IR (KBr) ν_{max}: 3016, 2941, 1599 cm^{-1}; ^1H NMR (300 MHz, $CDCl_3$) δ 9.19 (1H, s, ArH), 8.36-8.33 (3H, m, ArH), 7.68 (2H, d, J = 8.64 Hz, ArH), 5.53 (2H, s, NH_2), 4.18 (3H, s, OCH_3), 3.97 (3H, s, OCH_3), 3.37 (3H, s, CH_3); ^{13}C NMR (75 MHz, $CDCl_3$) δ 161.7, 161.4, 156.4, 151.1, 150.1, 149.6, 146.3, 144.9, 144.5, 129.4, 129.1, 128.7, 122.2, 118.1, 99.6, 60.7, 60.6, 56.1; ESMS *m/z*: 408.2 [M^+ + 1]; Anal. calcd for $C_{20}H_{17}N_5O_5$: C, 58.97; H, 4.21; N, 17.19; found: C, 58.93; H, 4.24; N, 17.15.

***N,N*-Dimethyl-*N*-(7,8,9-trimethoxy-6-(3,4-dimethoxyphenyl)pyrimido[5,4-*c*]isoquinolin-2-yl)amine (20i):** Yield = 84%; yellow solid; mp 126–128 °C; R_f 0.32 (3:7 EtOAc:hexane); IR (KBr) ν_{max}: 3020, 2933, 1595 cm^{-1}; ^1H NMR (300 MHz, $CDCl_3$) δ 9.18 (1H, s, ArH), 8.33 (1H, s, ArH), 7.10-7.08 (2H, m, ArH), 6.97-6.94 (1H, m, ArH), 4.14 (3H, s, OCH_3), 3.95 (6H, s, 2 x OCH_3), 3.91 (3H, s, OCH_3), 3.41 (6H, s, 2 x CH_3), 3.38 (3H, s, OCH_3); ^{13}C NMR (75 MHz, $CDCl_3$) δ 161.0, 159.8, 156.2, 153.9, 150.9, 148.5, 147.9, 145.3, 145.2, 136.4, 130.3, 128.6, 120.9, 119.4, 112.1, 110.4, 99.9, 61.3, 61.1, 56.0, 55.9, 37.4; ESMS *m/z*: 451.1 [M^+ + 1]; Anal. calcd for $C_{24}H_{26}N_4O_5$: C, 63.99; H, 5.82; N, 12.44; found: C, 63.97; H, 5.80; N, 12.40.

***N,N*-Dimethyl-*N*-(7,8,9-trimethoxy-6-(3,4-dichlorophenyl)pyrimido[5,4-*c*]isoquinolin-2-yl)amine (20j):** Yield = 83%; yellow solid; mp 122–124 °C; R_f 0.63 (3:7 EtOAc:hexane); IR

(KBr) ν_{max}: 3063, 2851, 1590 cm^{-1}; ^{1}H NMR (300 MHz, $CDCl_3$) δ 9.15 (1H, s, ArH), 8.31 (1H, s, ArH), 7.63 (1H, d, J = 1.38 Hz, ArH), 7.51 (1H, d, J = 8.19 Hz, ArH), 7.37-7.34 (1H, m, ArH), 4.13 (3H, s, OCH_3), 3.95 (3H, s, OCH_3), 3.42 (3H, s, OCH_3), 3.41 (6H, s, 2 x CH_3); ^{13}C NMR (75 MHz, $CDCl_3$) δ 156.6, 151.6, 150.4, 145.5, 145.2, 143.7, 135.9, 131.4, 131.4, 130.4, 130.3, 129.3, 128.7, 128.3, 128.0, 125.6, 119.0, 100.1, 61.2, 61.0, 56.2, 37.6; ESMS *m/z*: 459.2 [M^+ + 1]; Anal. calcd for $C_{22}H_{20}Cl_2N_4O_3$: C, 57.53; H, 4.39; N, 12.20; found: C, 57.57; H, 4.41; N, 12.25.

***N,N*-Dimethyl-*N*-(7,8,9-trimethoxy-6-(4-bromophenyl)pyrimido[5,4-*c*]isoquinolin-2-yl)amine (20k):**Yield = 87%; yellow solid; mp 190–192 °C; R_f 0.62 (3:7 EtOAc:hexane); IR (KBr) ν_{max}: 3018, 2944, 1596 cm^{-1}; ^{1}H NMR (300 MHz, $CDCl_3$) δ 9.15 (1H, s, ArH), 8.32 (1H, s, ArH), 7.57 (2H, d, J = 8.25 Hz, ArH), 7.40 (2H, d, J = 8.22 Hz, ArH), 4.13 (3H, s, OCH_3), 3.95 (3H, s, OCH_3), 3.40 (6H, s, 2 x CH_3), 3.36 (3H, s, OCH_3); ^{13}C NMR (75 MHz, $CDCl_3$) δ 161.2, 159.9, 156.4, 153.0, 150.6, 145.4, 145.2, 142.6, 130.5, 130.1, 128.7, 119.2, 100.1, 61.2, 61.1, 56.1, 37.5; ESMS *m/z*: 469.2 [M^+ + 1]; Anal. calcd for $C_{22}H_{21}BrN_4O_3$; C, 56.30; H, 4.51; N, 11.94.; found C, 56.32; H, 4.55; N, 11.96

***N,N*-Dimethyl-*N*-(7,8,9-trimethoxy-6-(4-methoxyphenyl)pyrimido[5,4-*c*]isoquinolin-2-yl)amine (20l):** Yield = 95%; yellow solid; mp 154–156 °C; R_f 0.37 (3:7 EtOAc:hexane); IR (KBr) ν_{max}: 3010, 2942, 1606 cm^{-1}; ^{1}H NMR (300 MHz, $CDCl_3$) δ 9.17 (1H, s, ArH), 8.33 (1H, s, ArH), 7.47 (2H, d, J = 8.55 Hz, ArH), 6.98 (2H, d, J = 8.52 Hz, ArH), 4.13 (3H, s, OCH_3), 3.96 (3H, s, OCH_3), 3.88 (6H, s, 2 x OCH_3), 3.40 (6H, s, 2 x CH_3); ^{13}C NMR (75 MHz, $CDCl_3$) δ 161.1, 159.9, 159.1, 156.2, 154.1, 152.6, 151.0, 148.7, 145.3, 136.2, 130.4, 129.8, 128.8, 119.5, 114.3, 112.9, 107.9, 100.1, 61.3, 56.1, 55.4, 37.6; ESMS *m/z*: 421.2 [M^+ + 1]; Anal. calcd for $C_{23}H_{24}N_4O_4$: C, 65.70; H, 5.75; N, 13.33; found: C, 65.72; H, 5.78; N, 13.30.

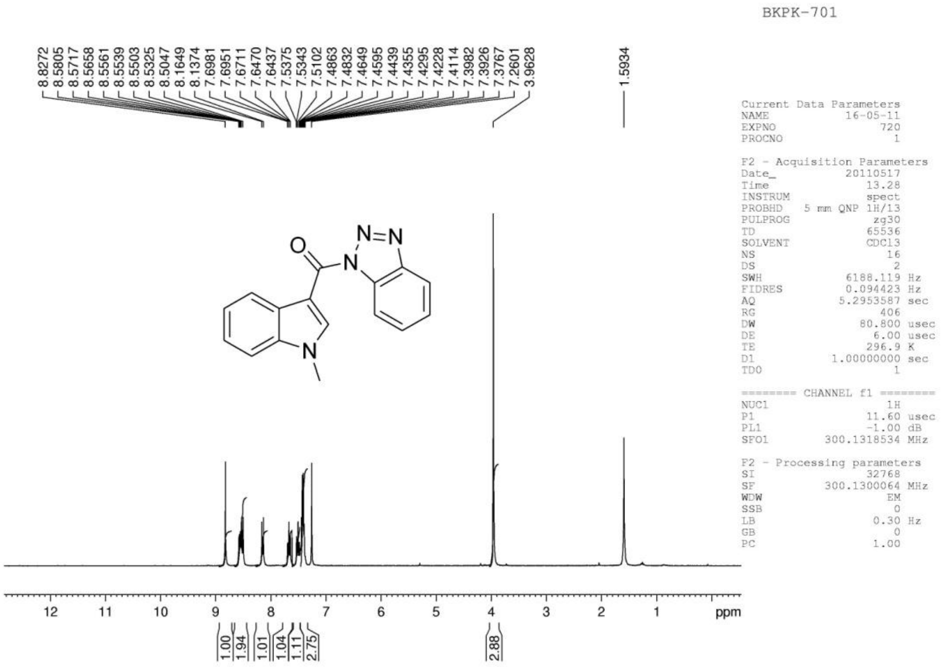

Figure 1: ^{1}H NMR of **4a**.

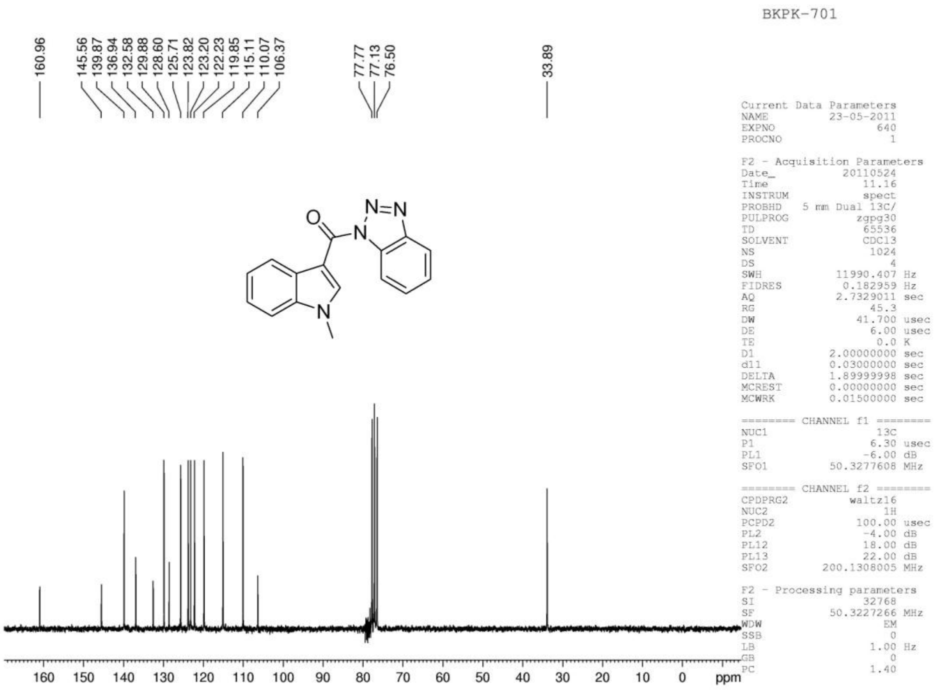

Figure 2: ^{13}C NMR of **4a**.

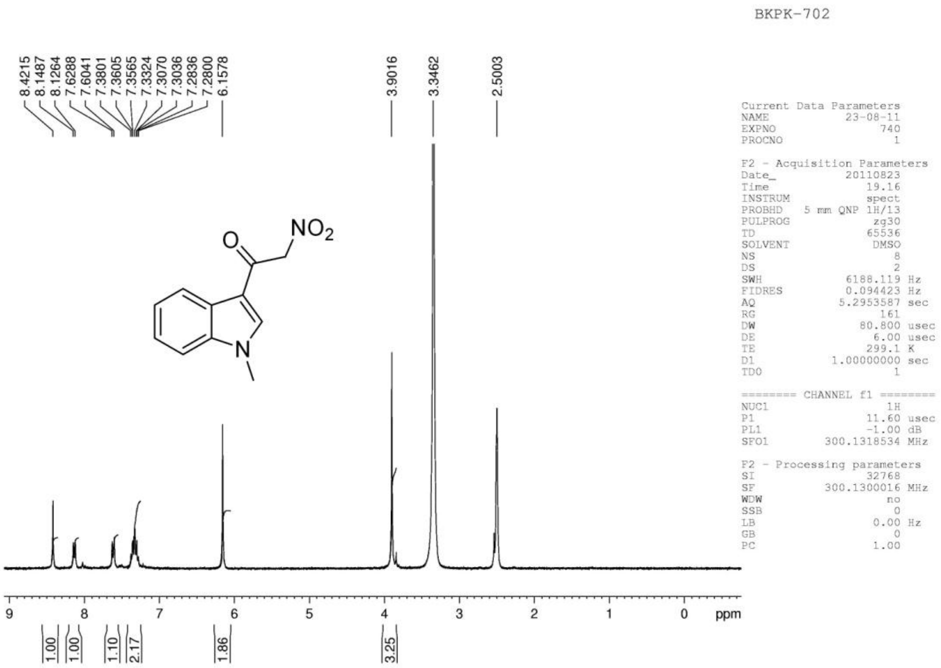

Figure 3: ^{1}H NMR of **5a**.

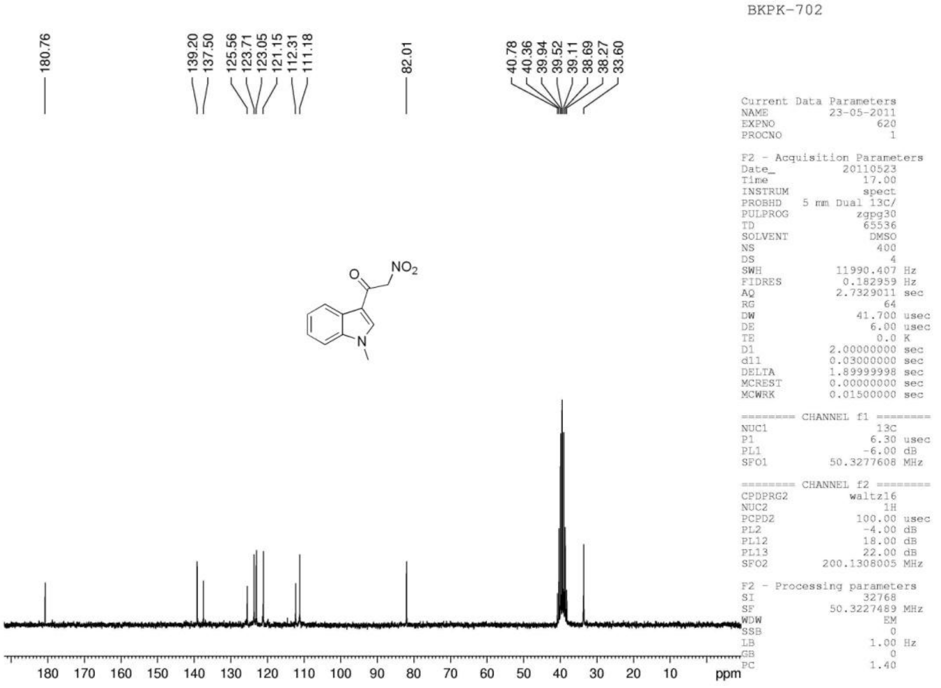

Figure 4: ^{13}C NMR of **5a**.

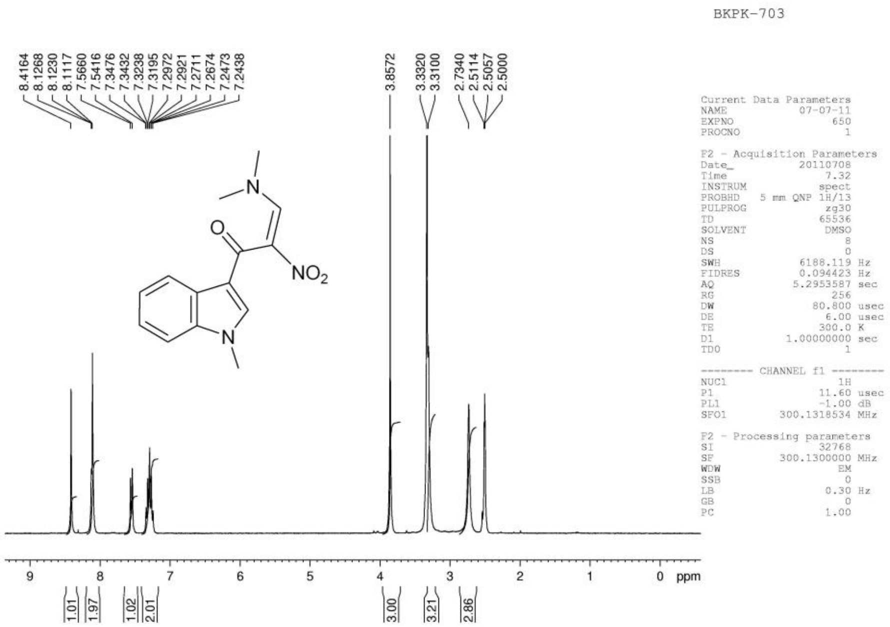

Figure 5: ^{1}H NMR of **6a**.

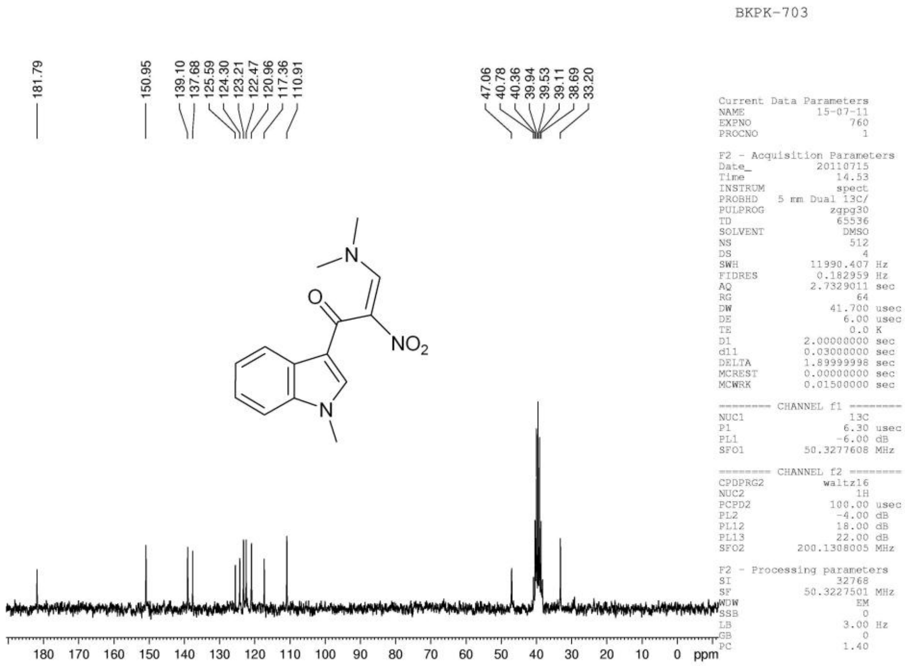

Figure 6: ^{13}C NMR of **6a**.

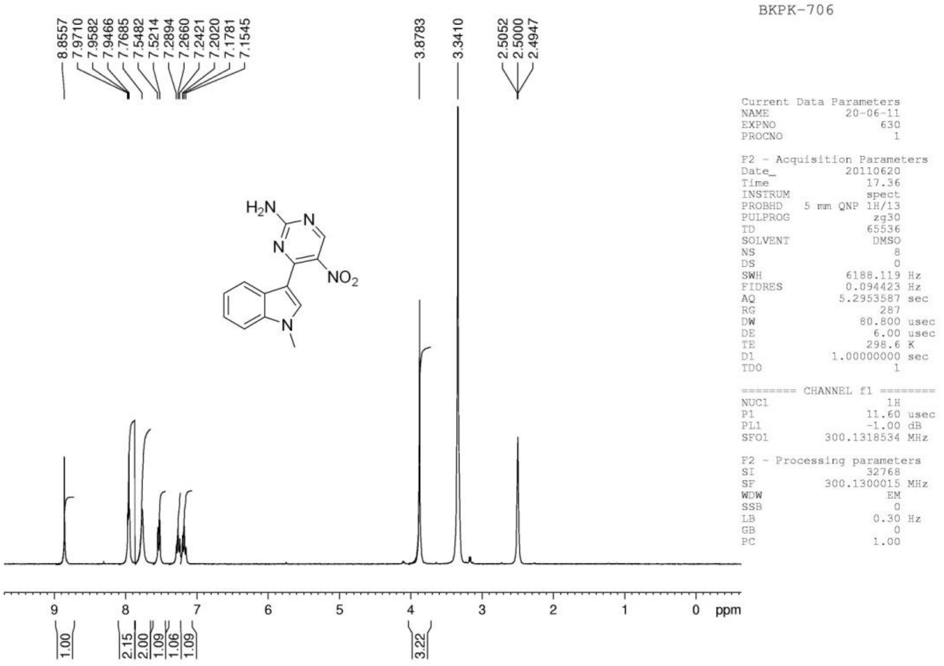

Figure 7: ^{1}H NMR of **7a**.

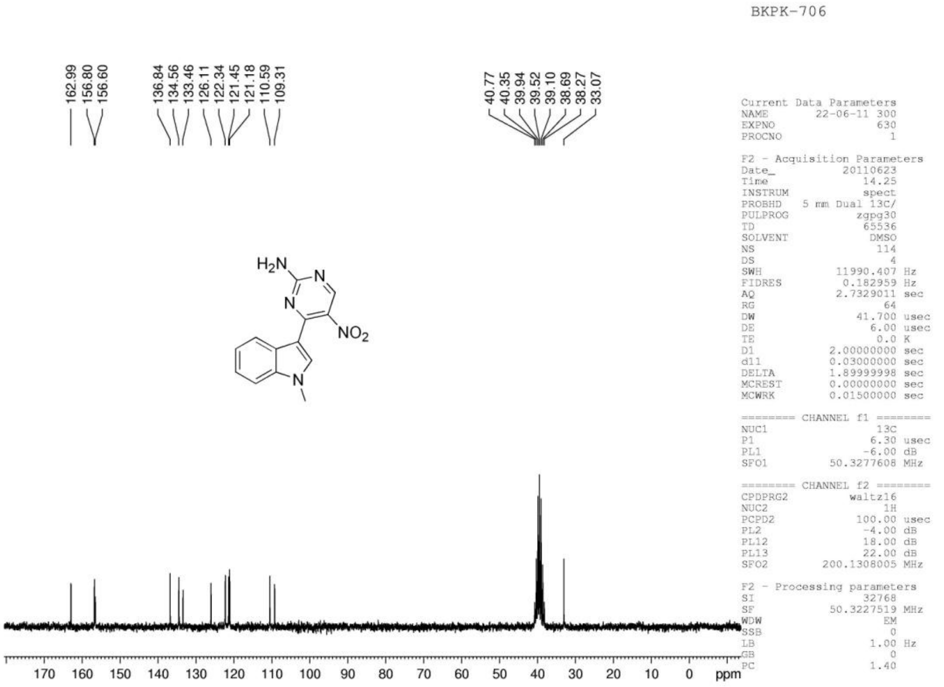

Figure 8: ^{13}C NMR of **7a**.

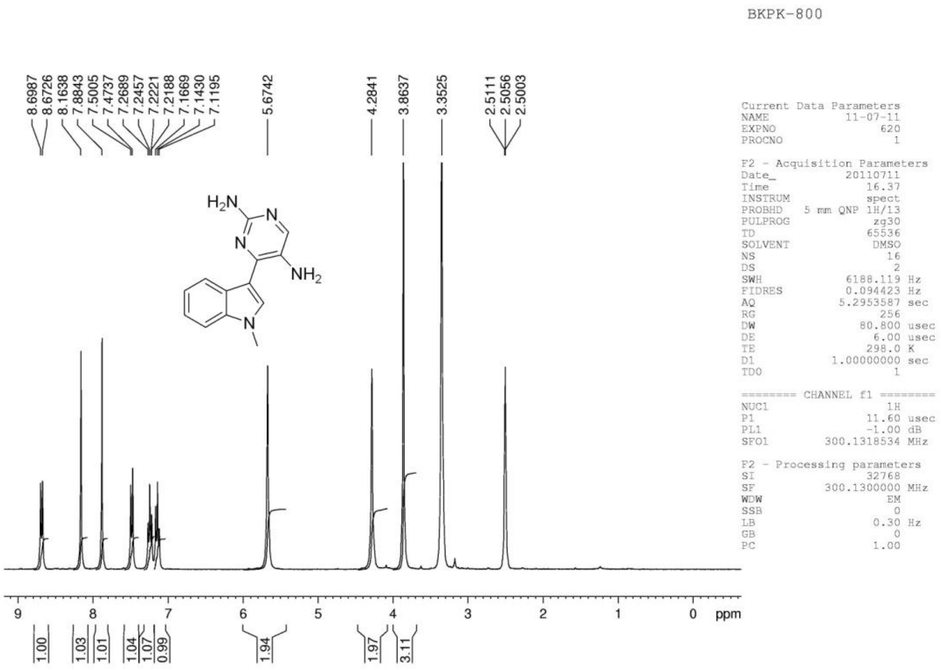

Figure 9: ^{1}H NMR of **2a**.

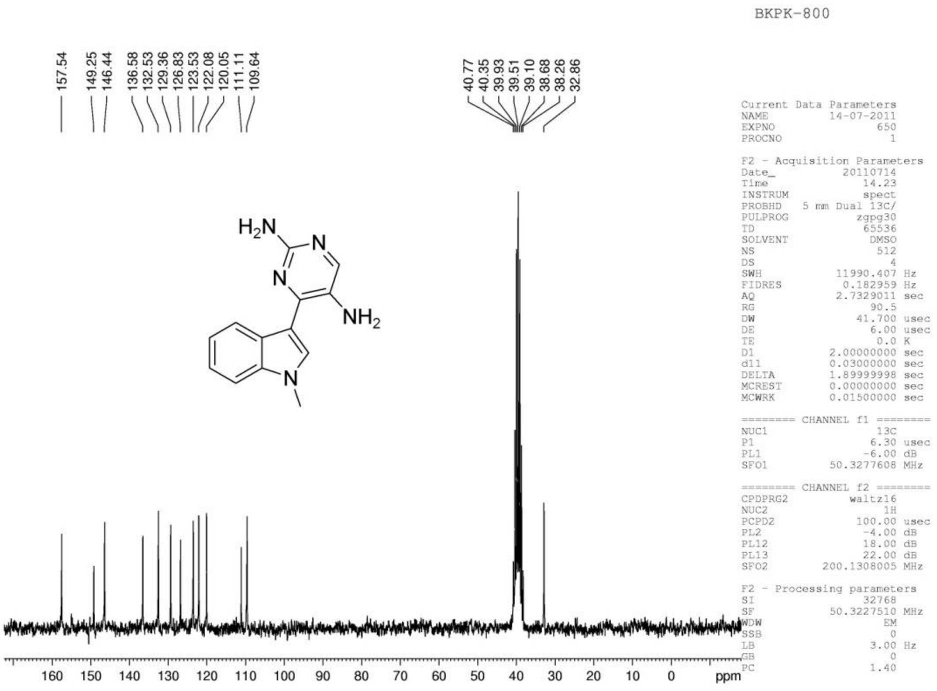

Figure 10: ^{13}C NMR of **2a**.

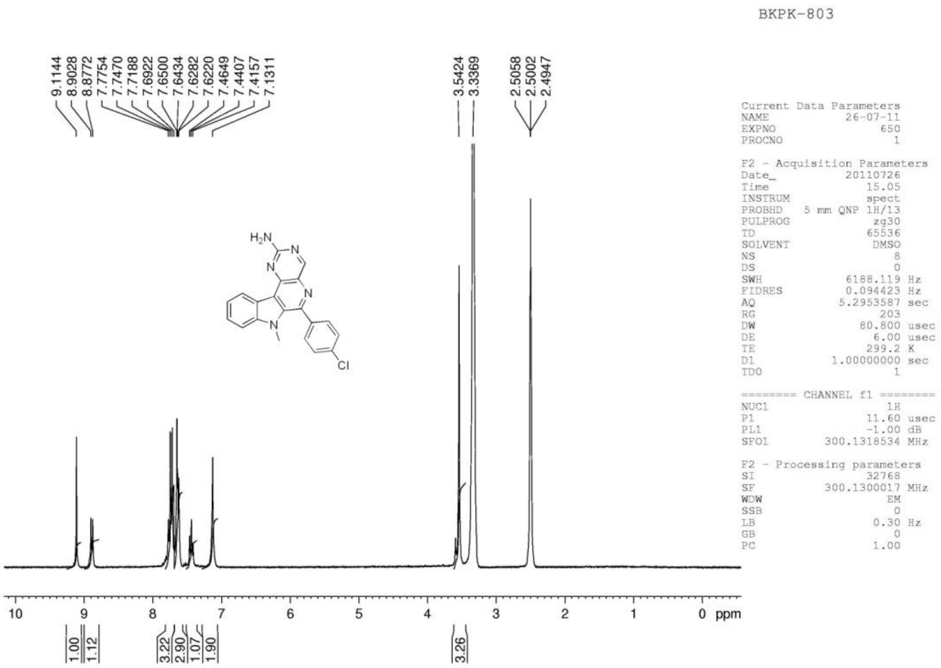

Figure 11: ^{1}H NMR of **9a**.

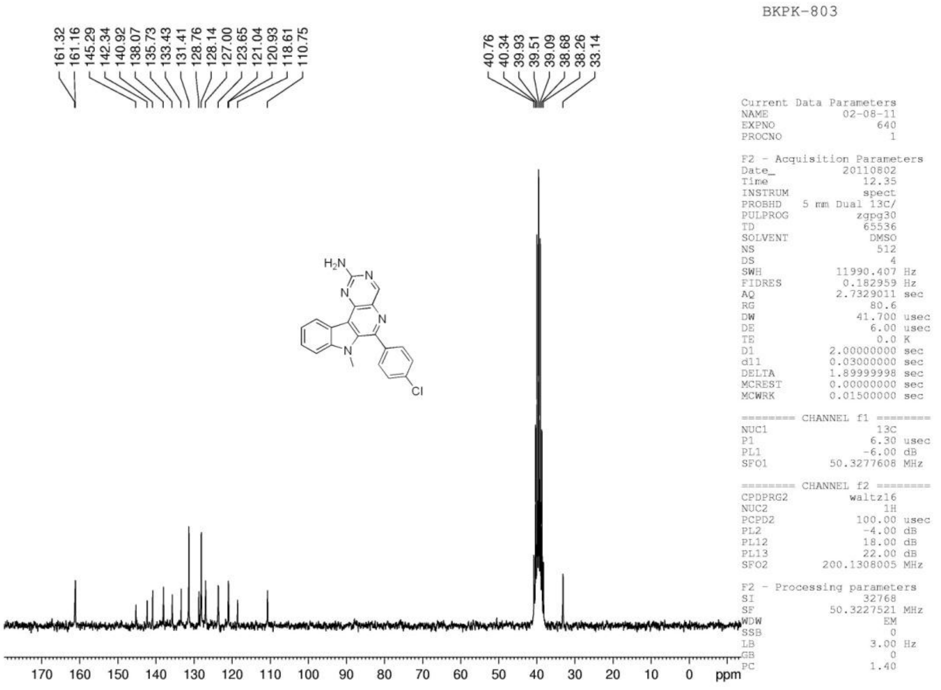

Figure 12: ^{13}C NMR of **9a**.

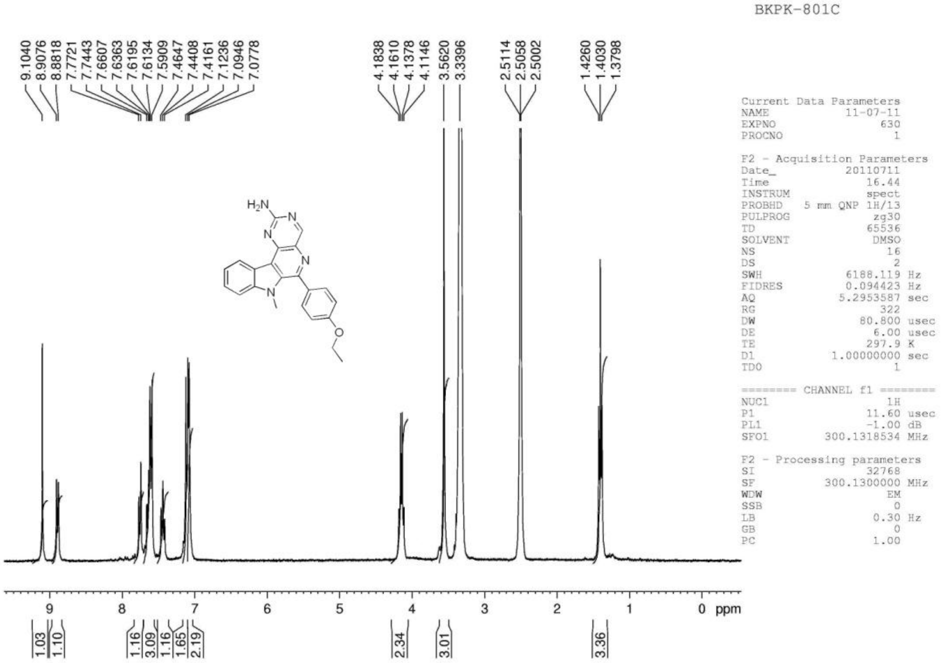

Figure 13: ^{1}H NMR of **9b**.

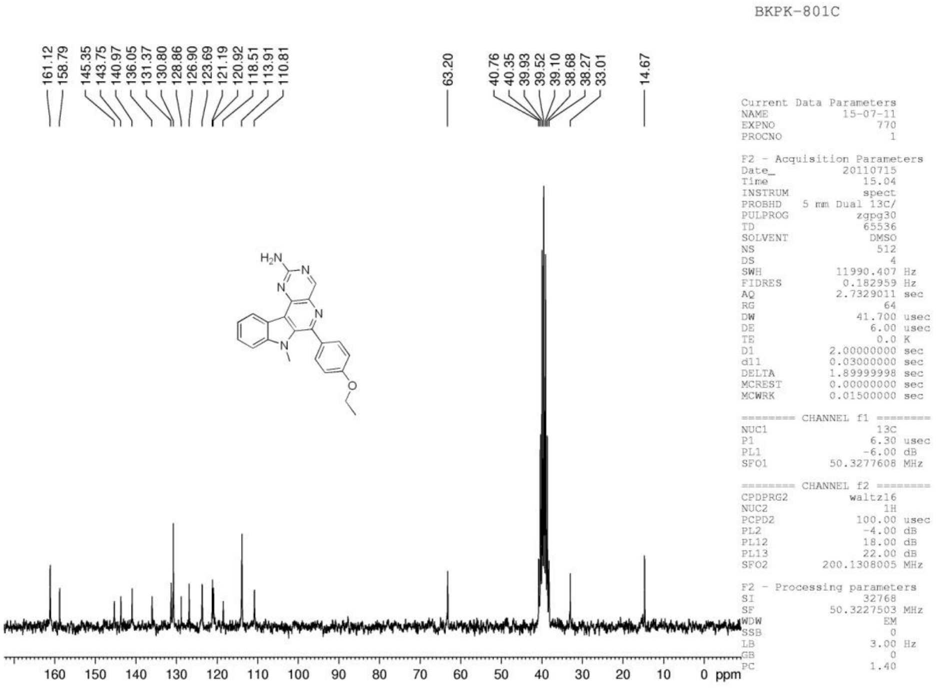

Figure 14: ^{13}C NMR of **9b**.

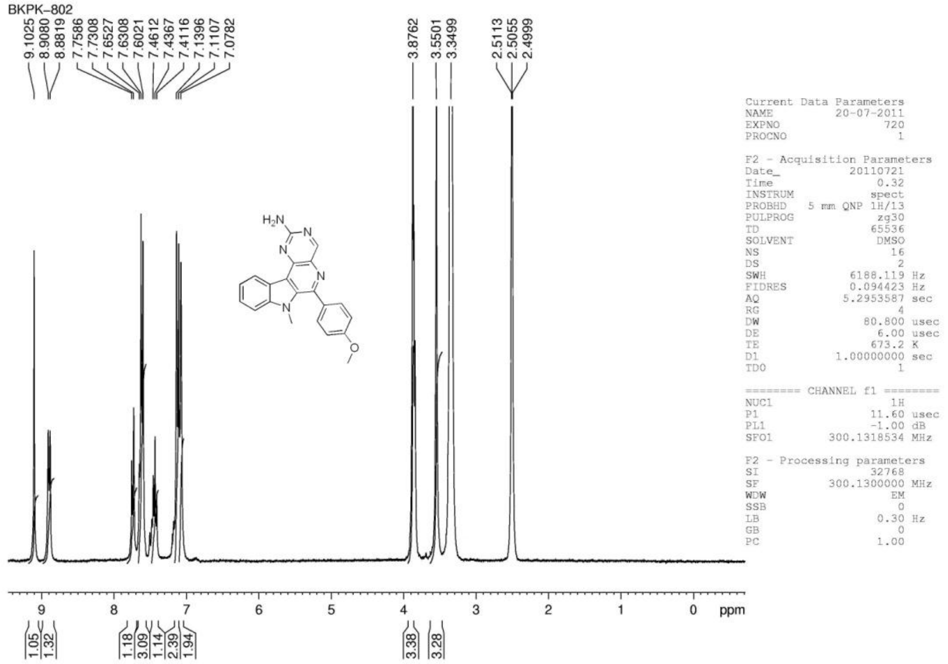

Figure 15: ^{1}H NMR of **9c**.

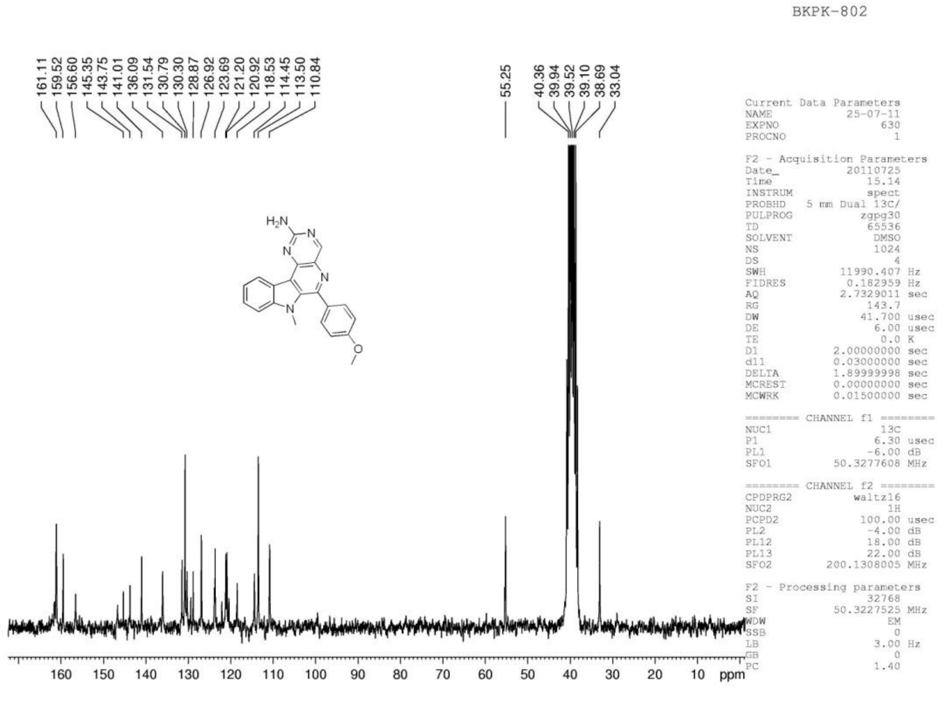

Figure 16: ^{13}C NMR of **9c**.

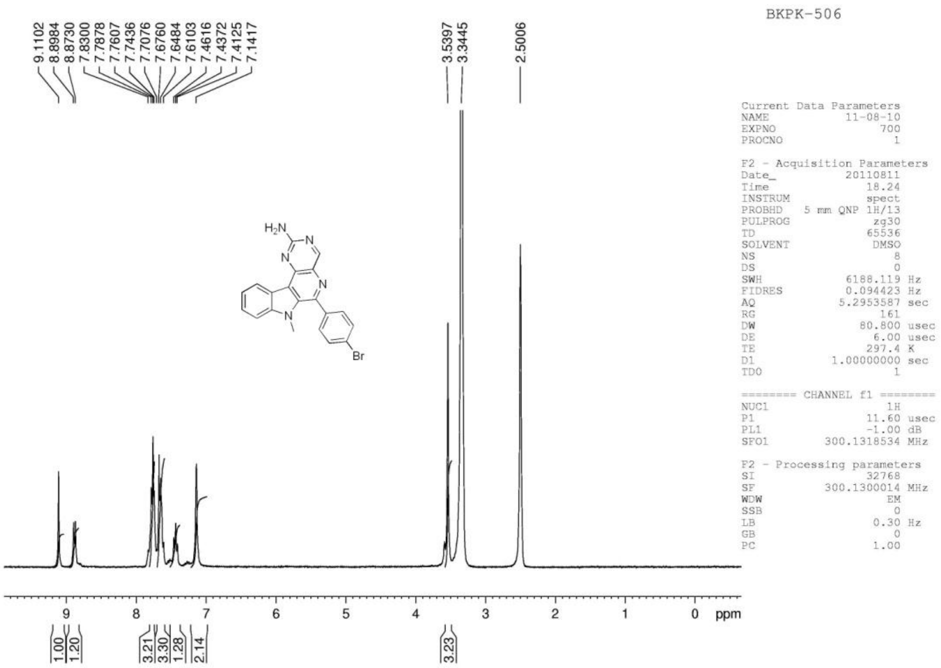

Figure 17: ^{1}H NMR of **9d**.

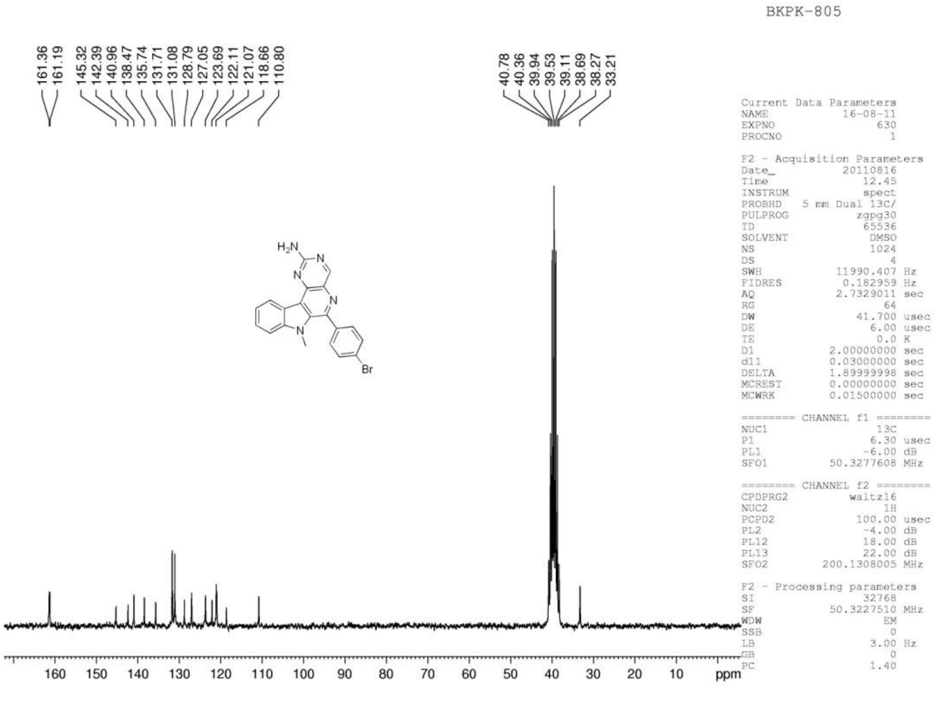

Figure 18: ^{13}C NMR of **9d**.

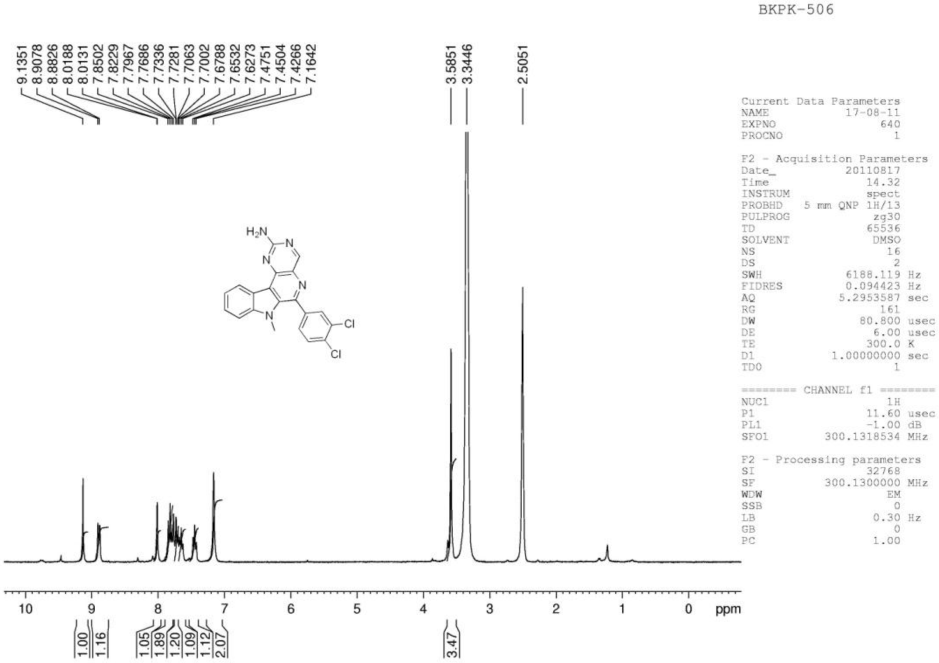

Figure 19: ^{1}H NMR of **9e**.

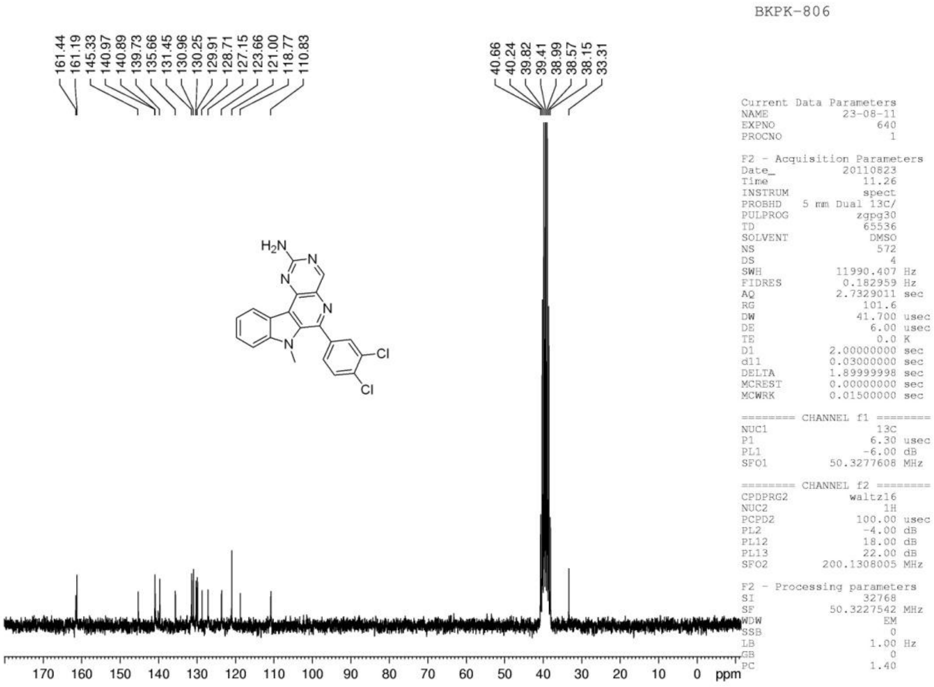

Figure 20: ^{13}C NMR of **9e**.

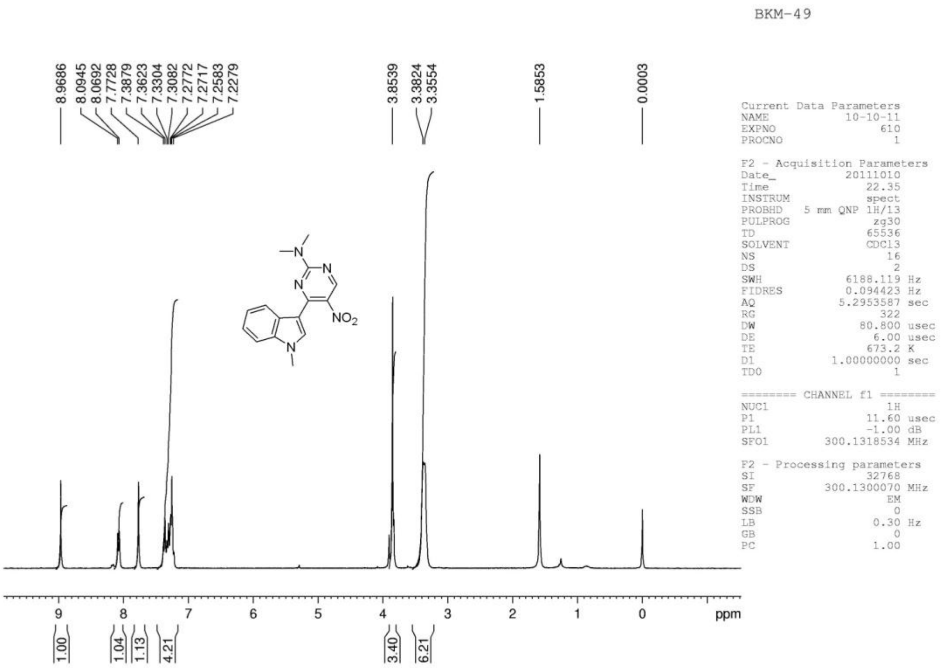

Figure 21: ^{1}H NMR of **8**.

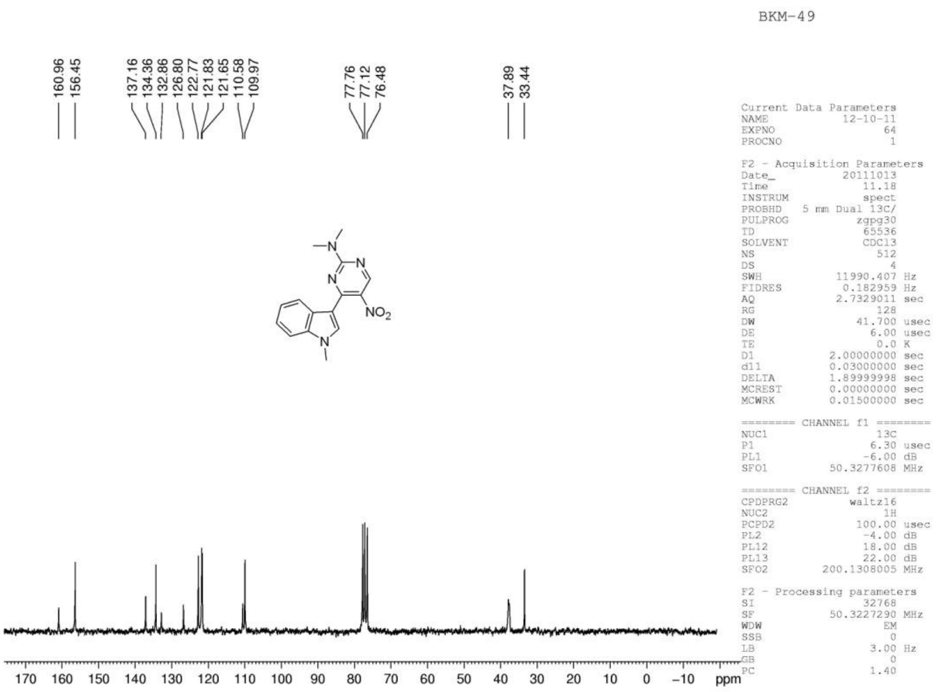

Figure 22: ^{13}C NMR of **8**.

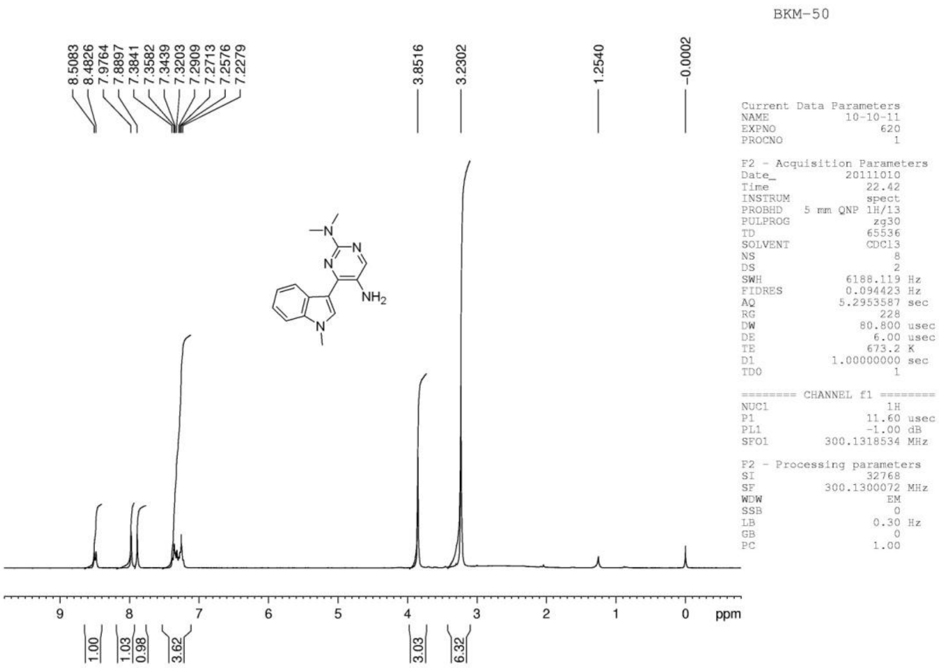

Figure 23: ^{1}H NMR of **2c**.

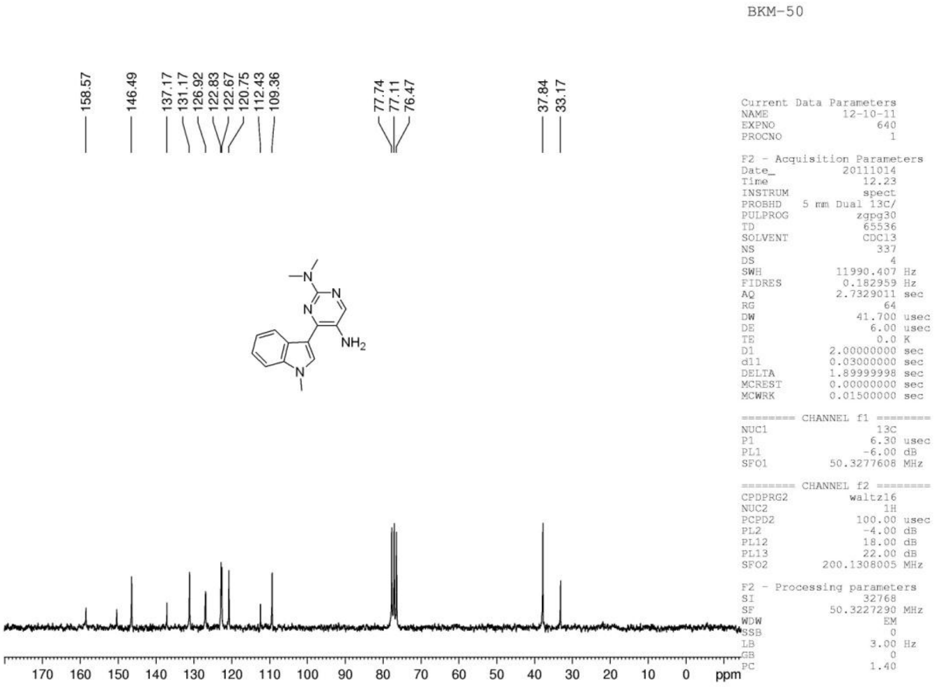

Figure 24: ^{13}C NMR of **2c**.

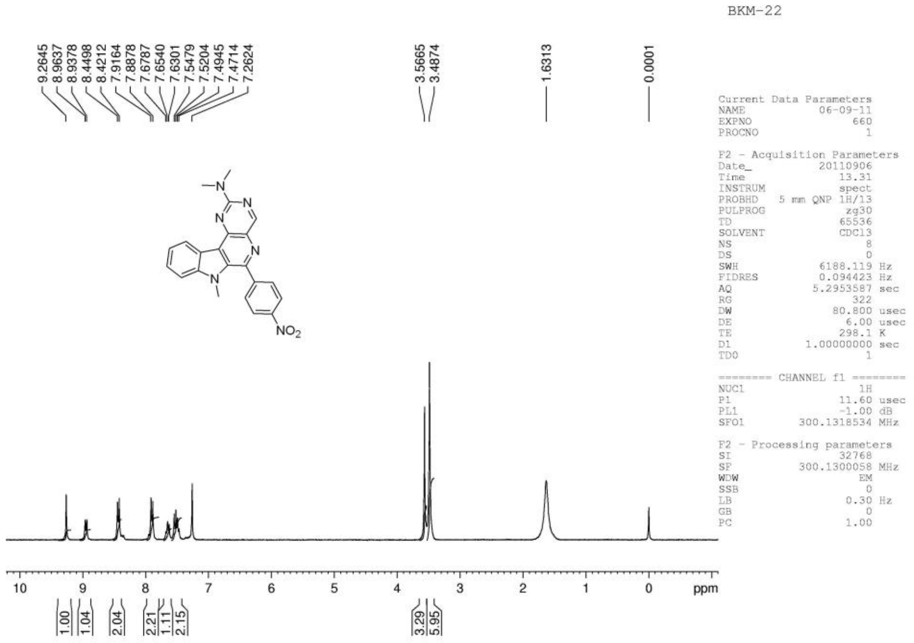

Figure 25: ^{1}H NMR of **9g**.

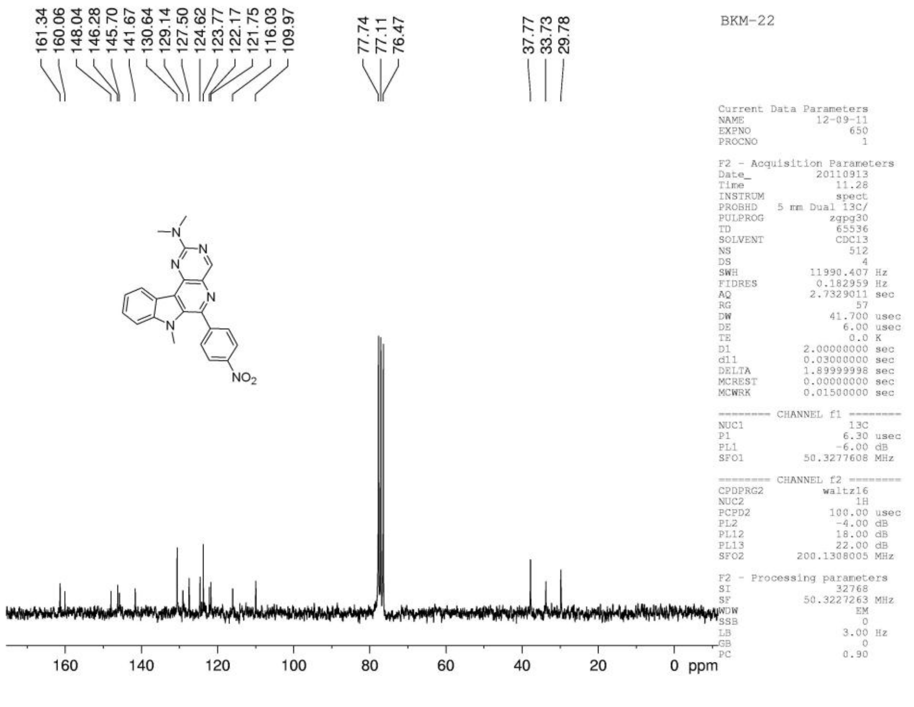

Figure 26: ^{13}C NMR of **9g**.

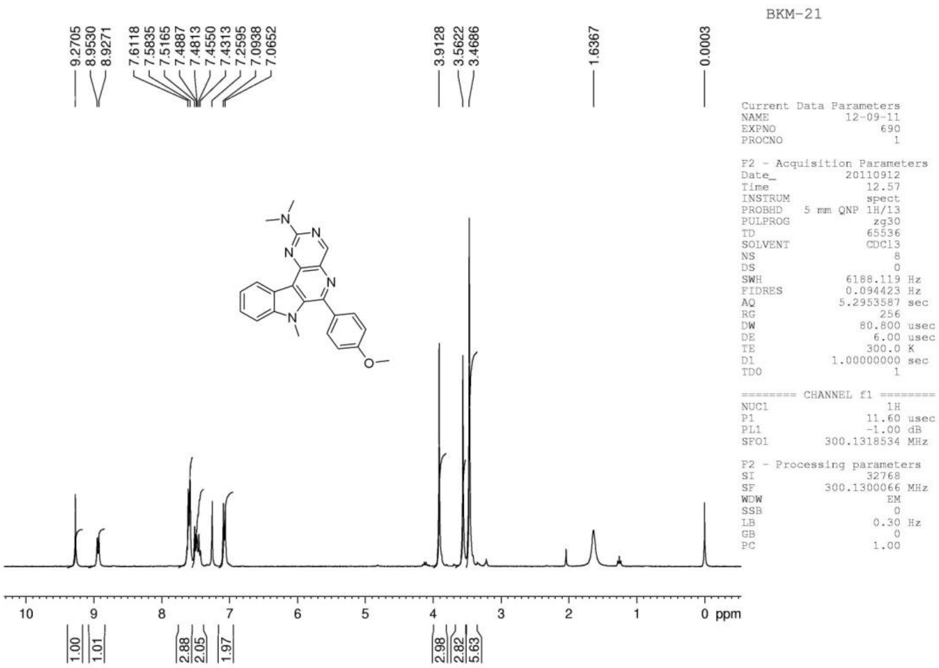

Figure 27: ^{1}H NMR of **9h**.

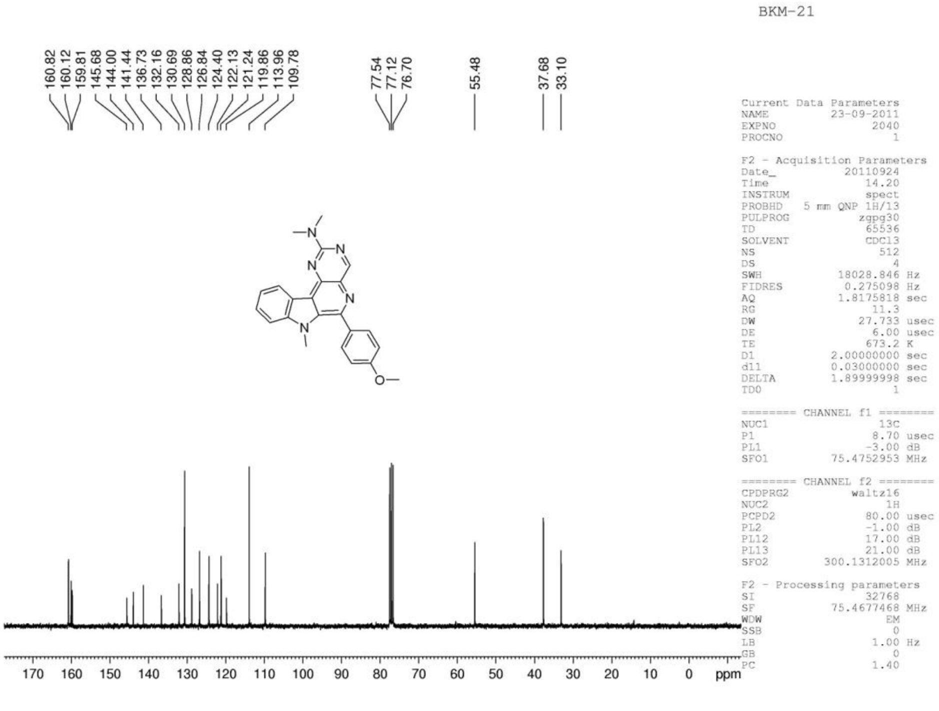

Figure 28: ^{13}C NMR of **9h**.

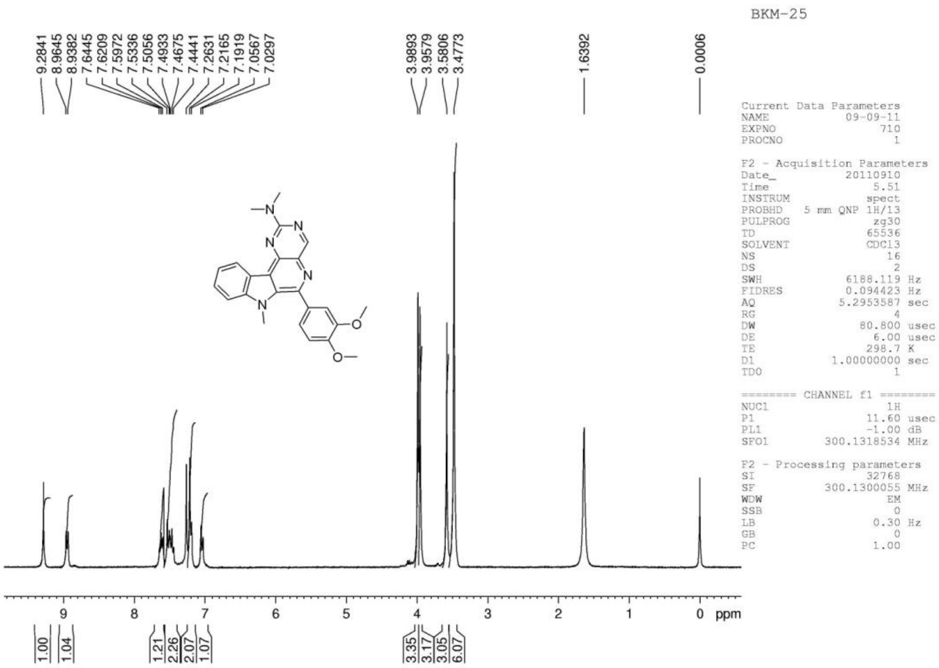

Figure 29: ^{1}H NMR of **9i**.

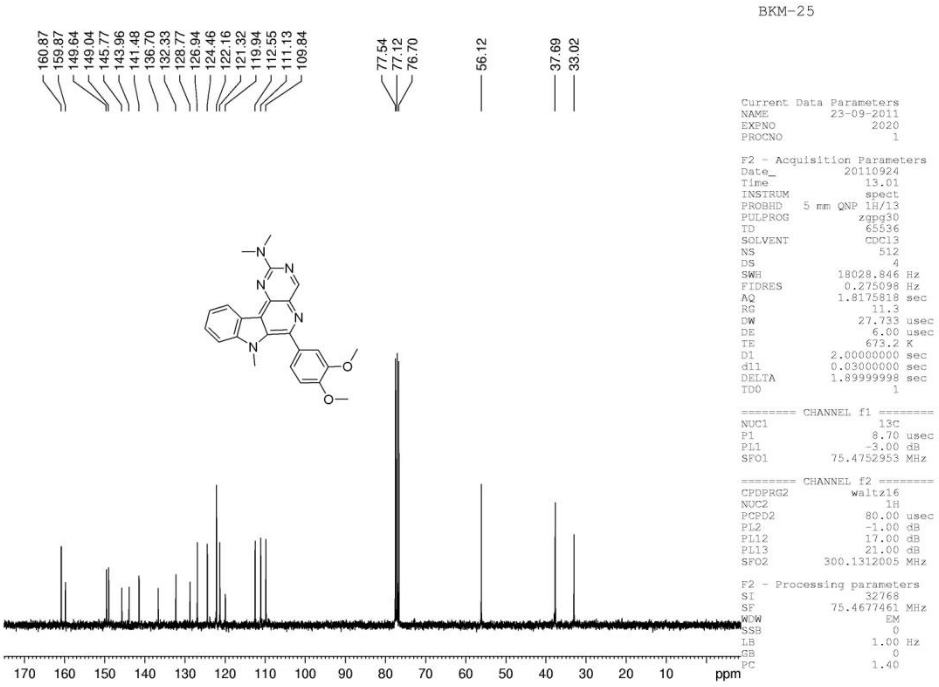

Figure 30: ^{13}C NMR of **9i**.

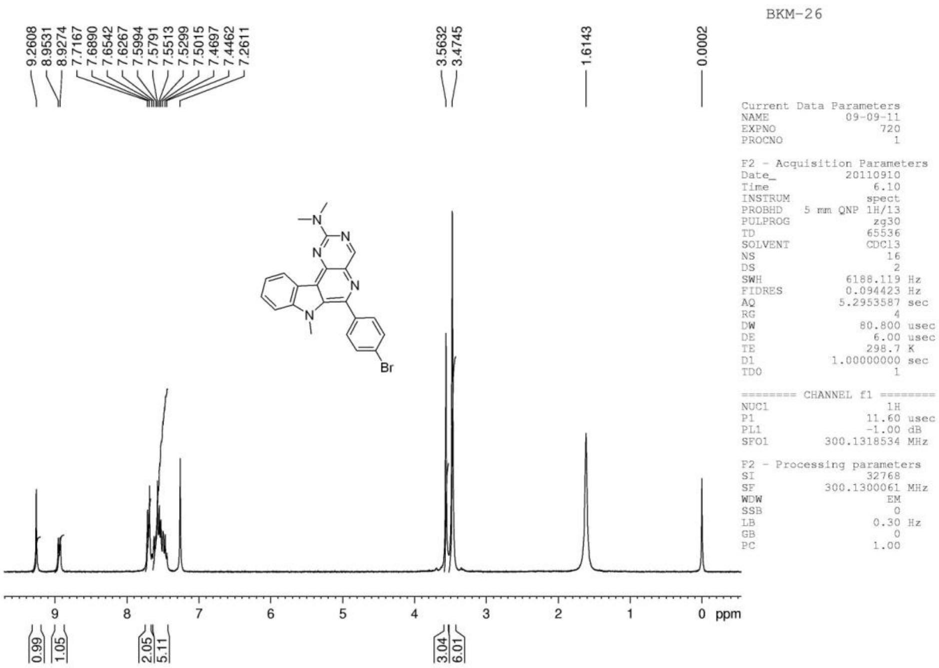

Figure 31: ^{1}H NMR of **9f**.

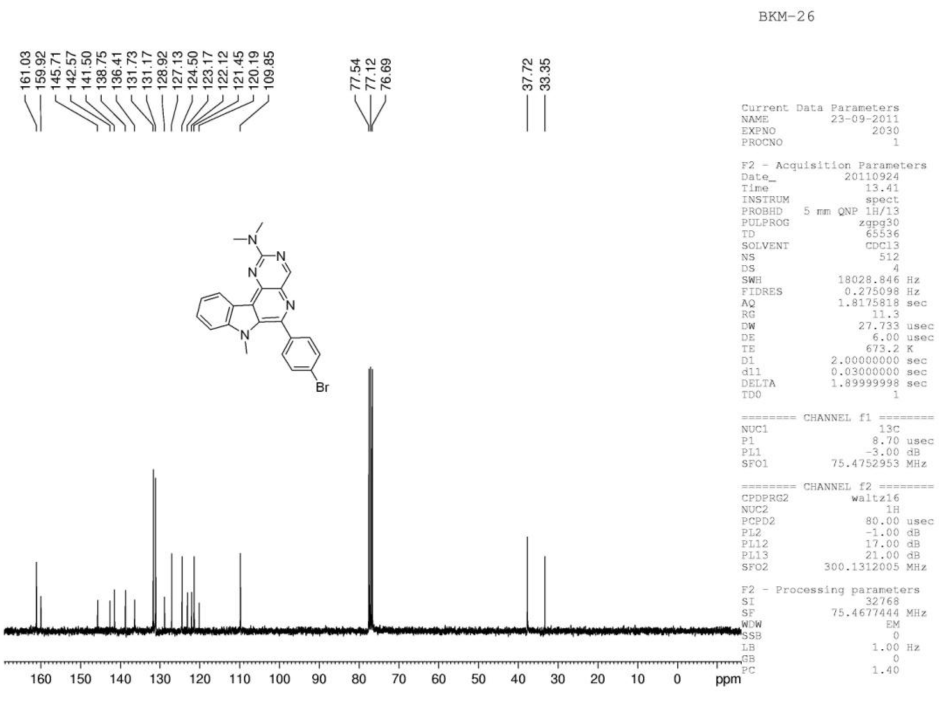

Figure 32: ^{13}C NMR of **9f**.

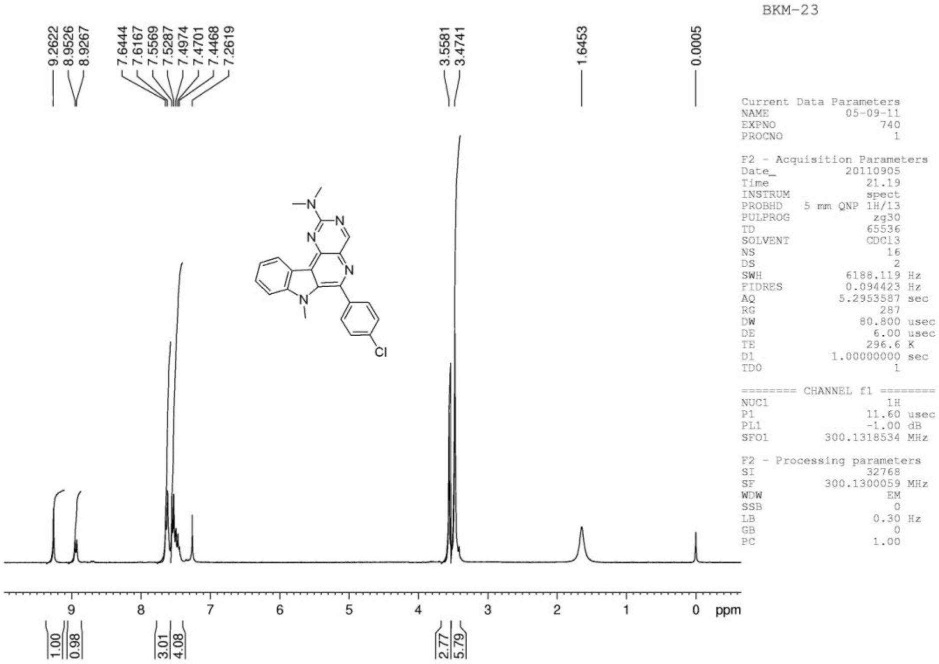

Figure 33: ^{1}H NMR of **9j**.

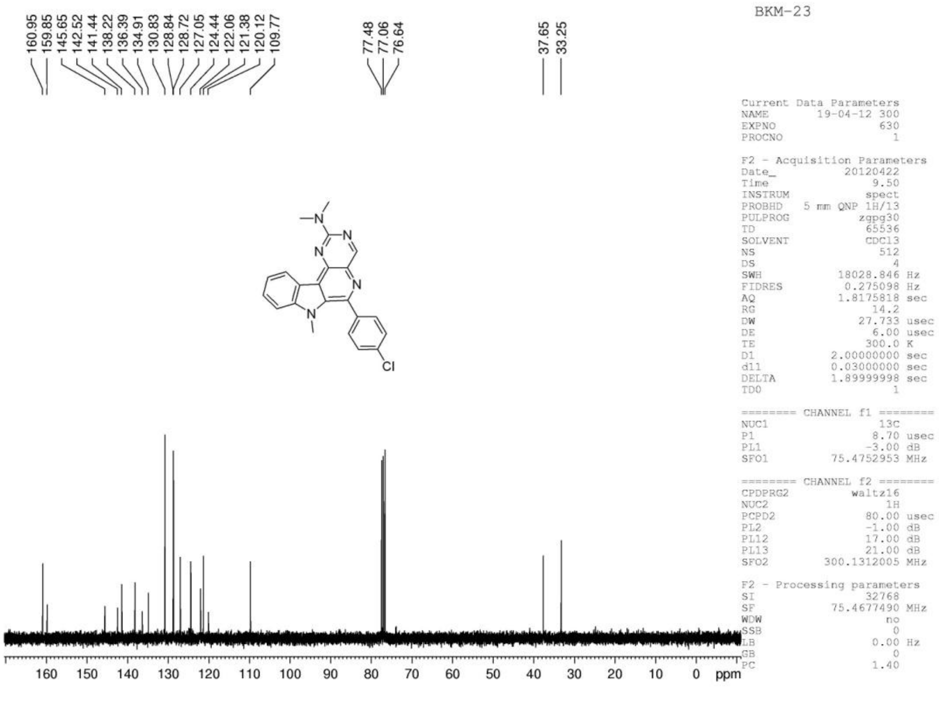

Figure 34: ^{13}C NMR of **9j**.

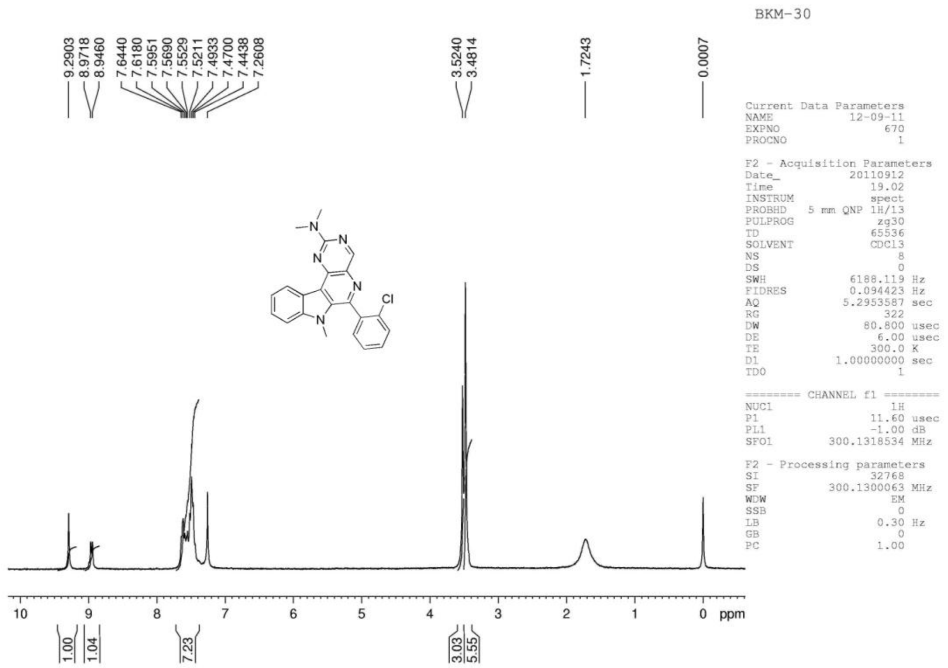

Figure 35: ^{1}H NMR of **9k**.

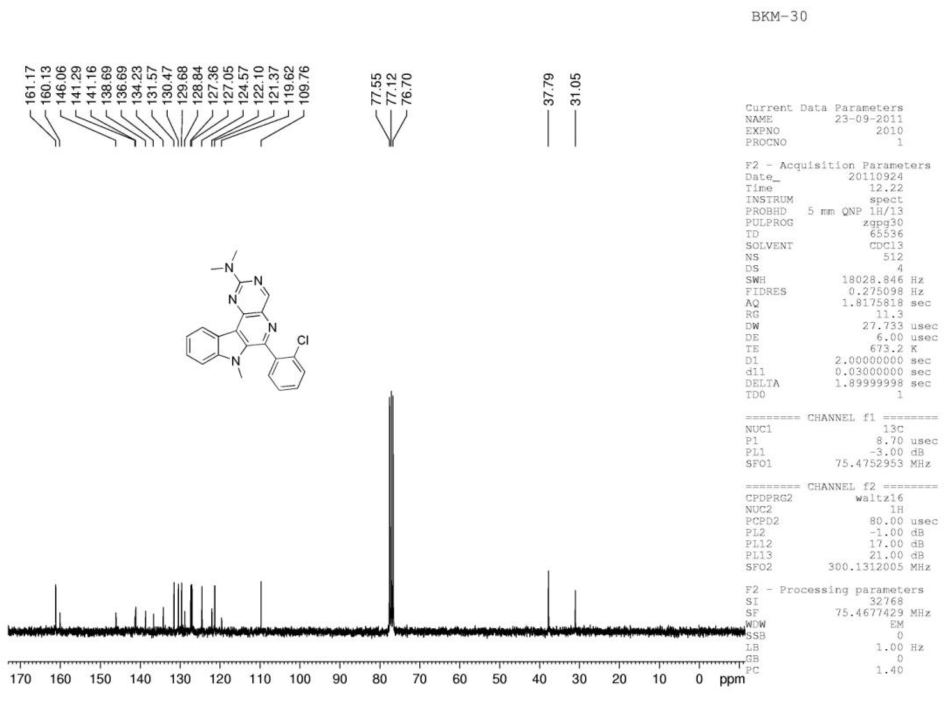

Figure 36: ^{13}C NMR of **9k**.

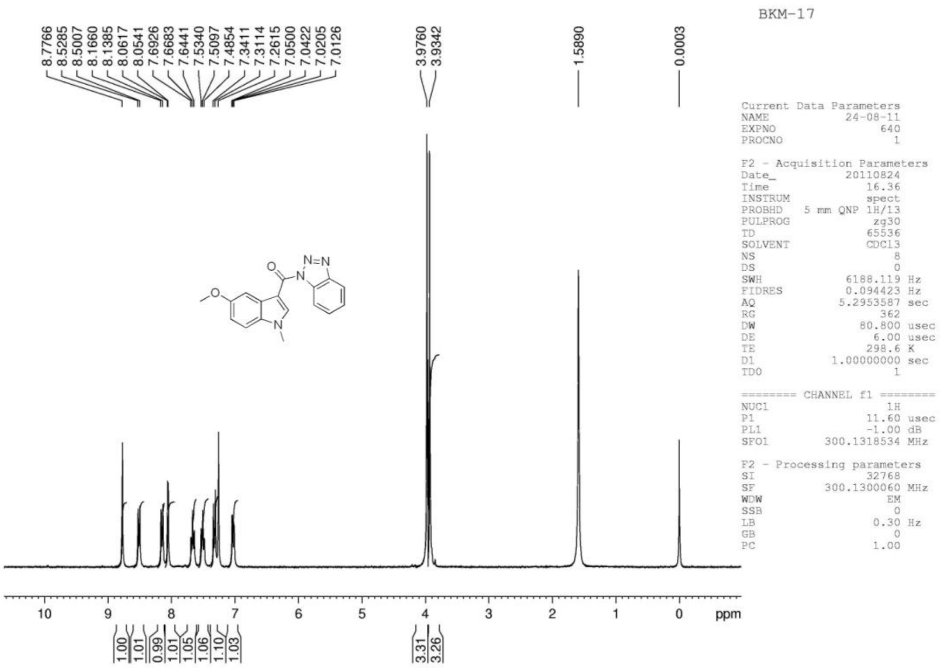

Figure 37: ^{1}H NMR of **4b**.

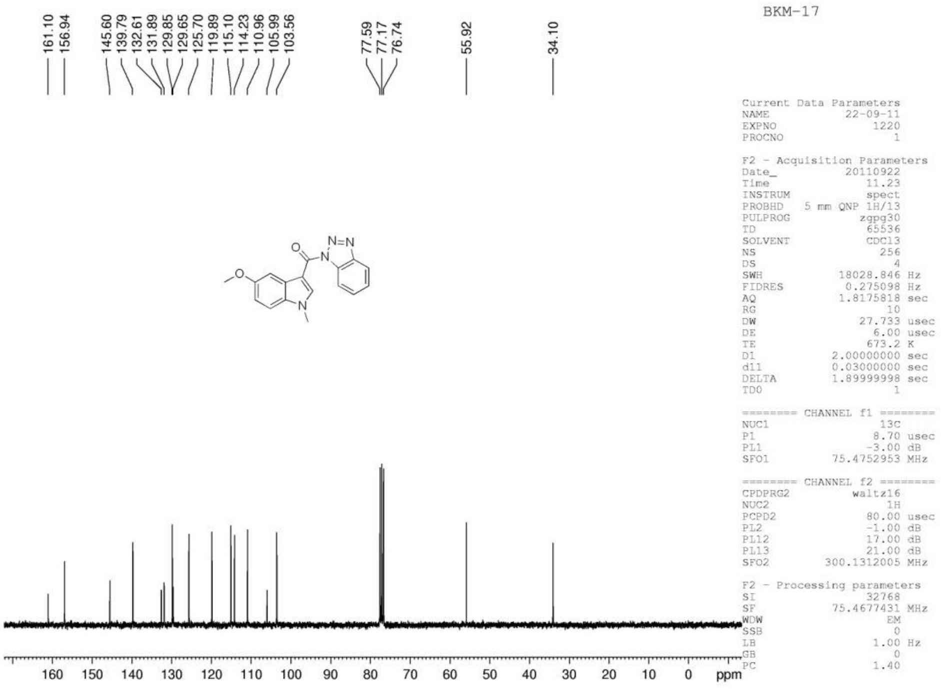

Figure 38: ^{13}C NMR of **4b**.

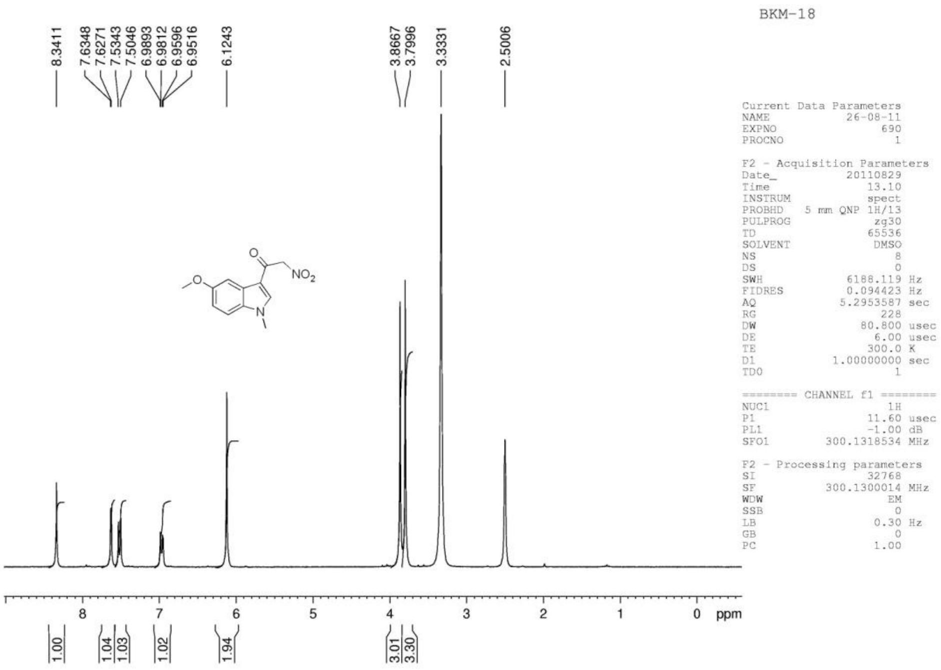

Figure 39: ^{1}H NMR of **5b**.

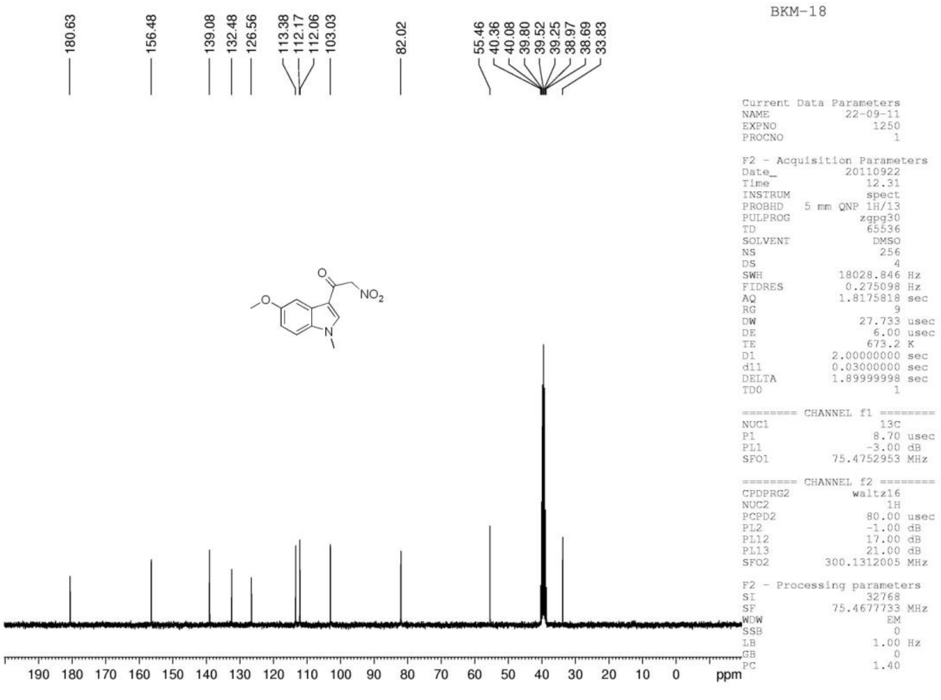

Figure 40: ^{13}C NMR of **5b**.

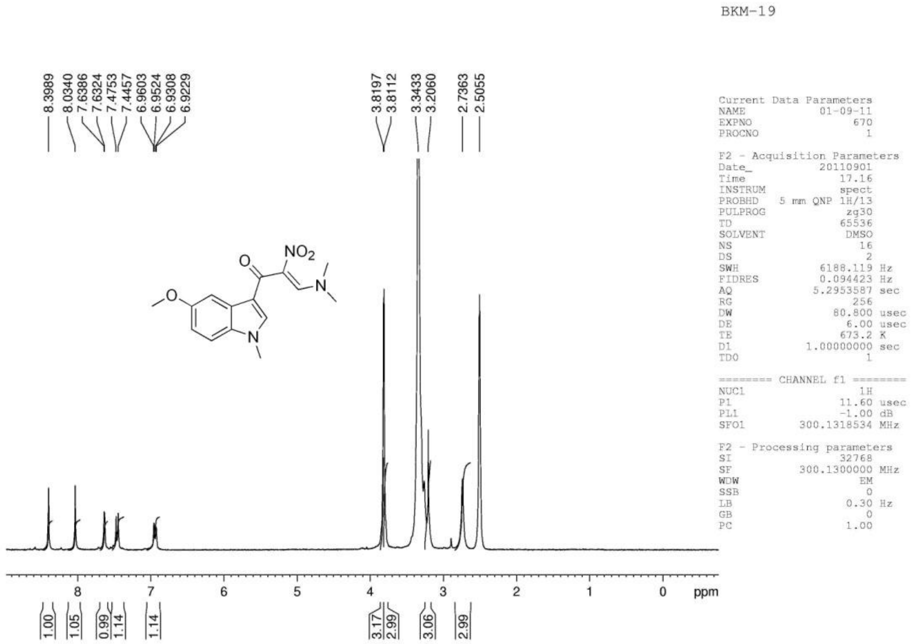

Figure 41: ^{1}H NMR of **6b**.

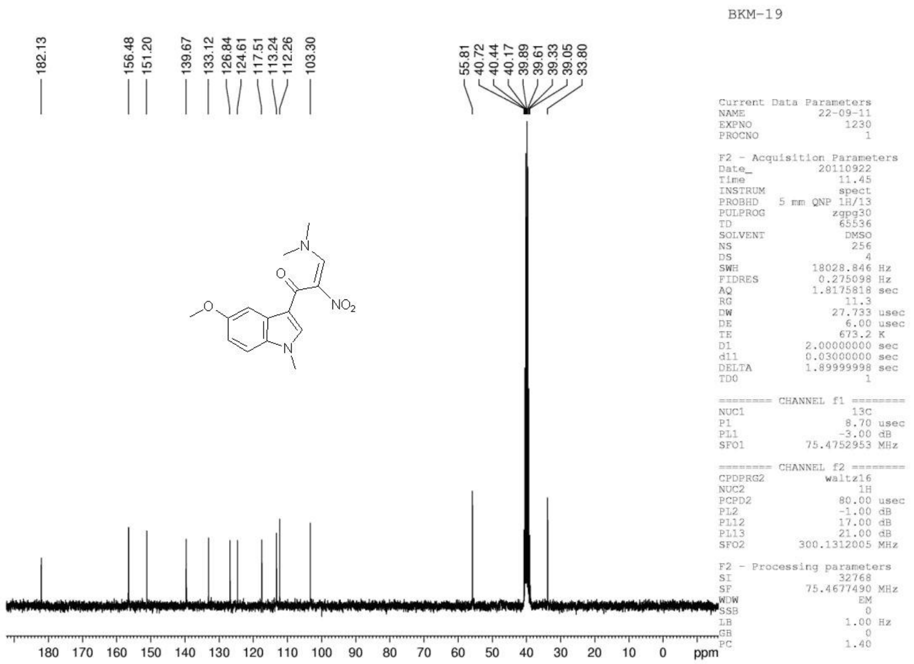

Figure 42: ^{13}C NMR of **6b**.

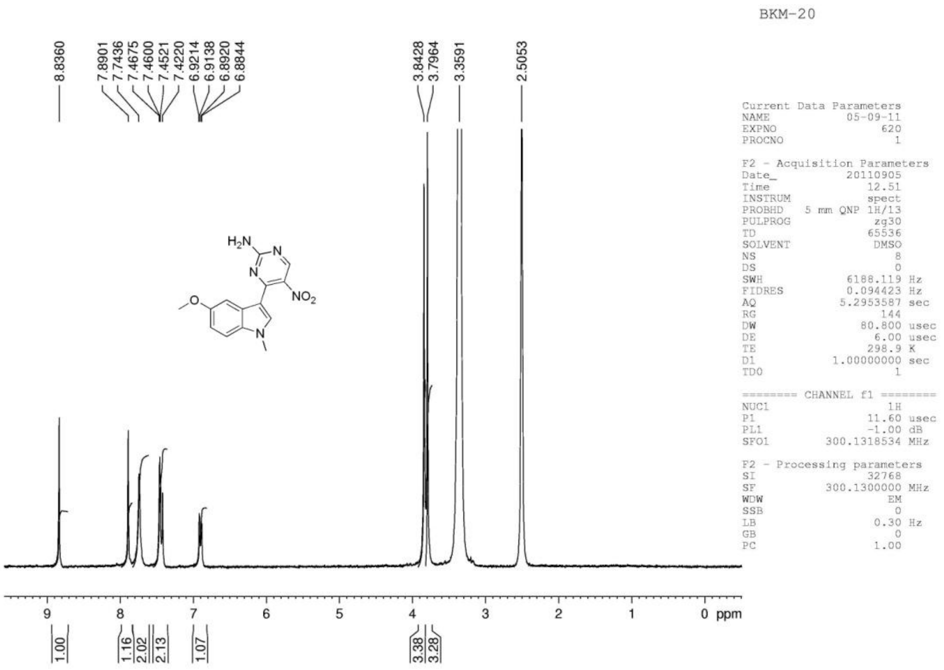

Figure 43: ^{1}H NMR of **7b**.

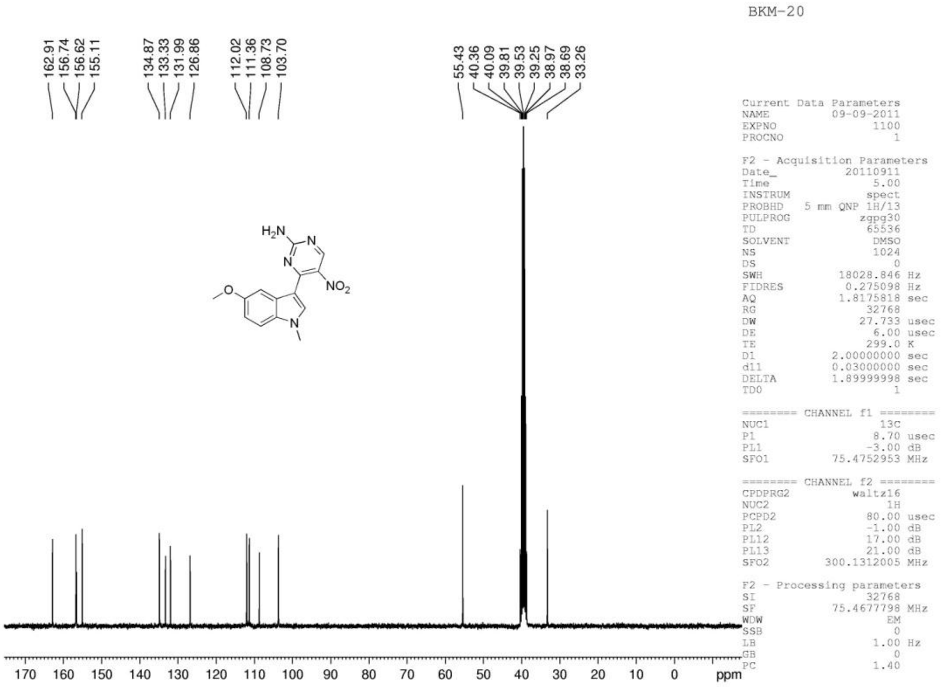

Figure 44: ^{13}C NMR of **7b**.

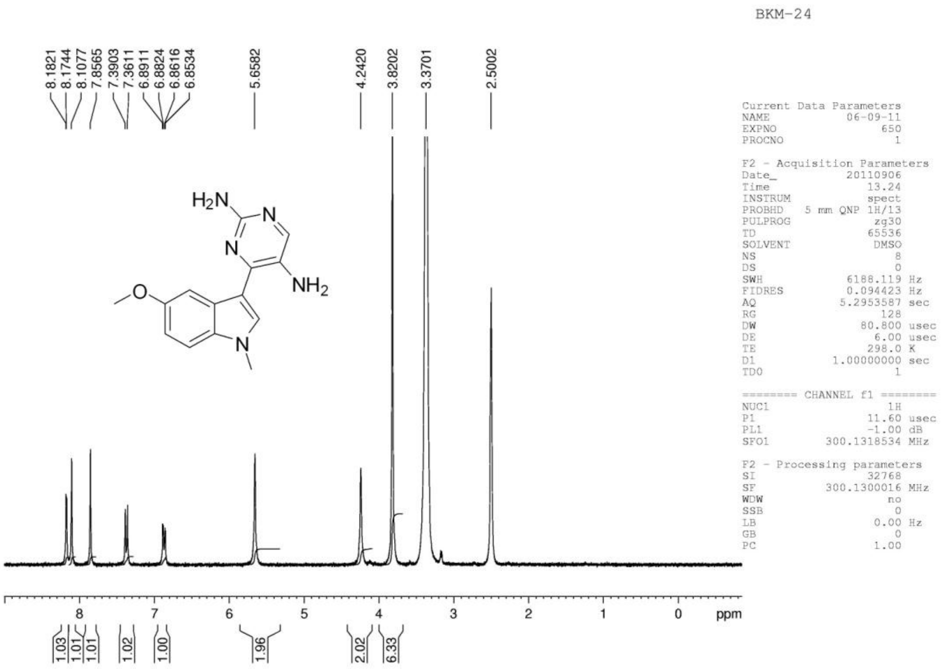

Figure 45: ^{1}H NMR of **2b**.

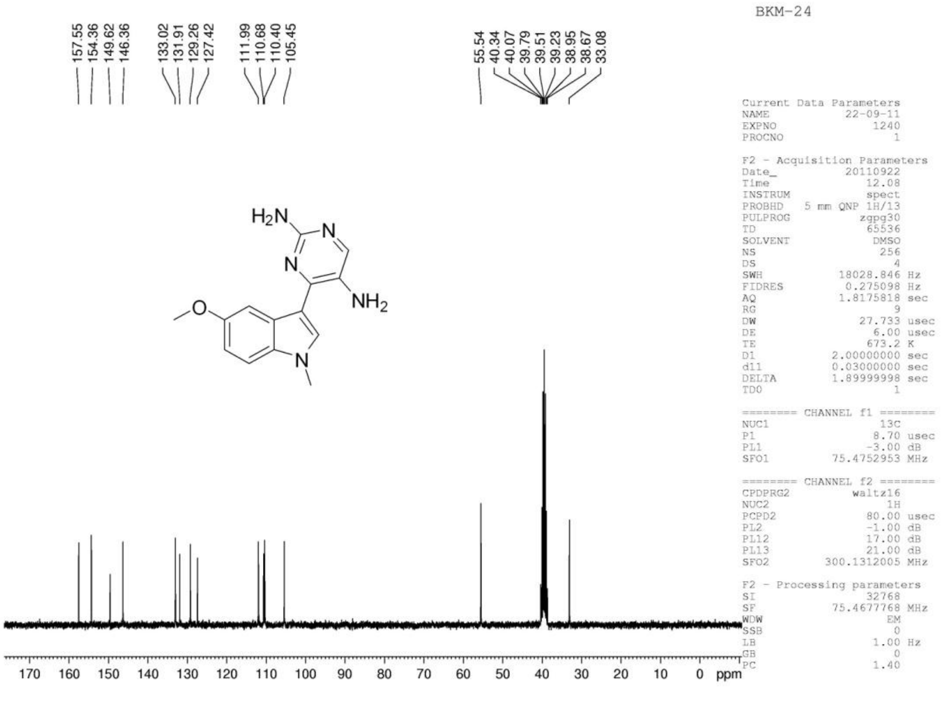

Figure 46: ^{13}C NMR of **2b**.

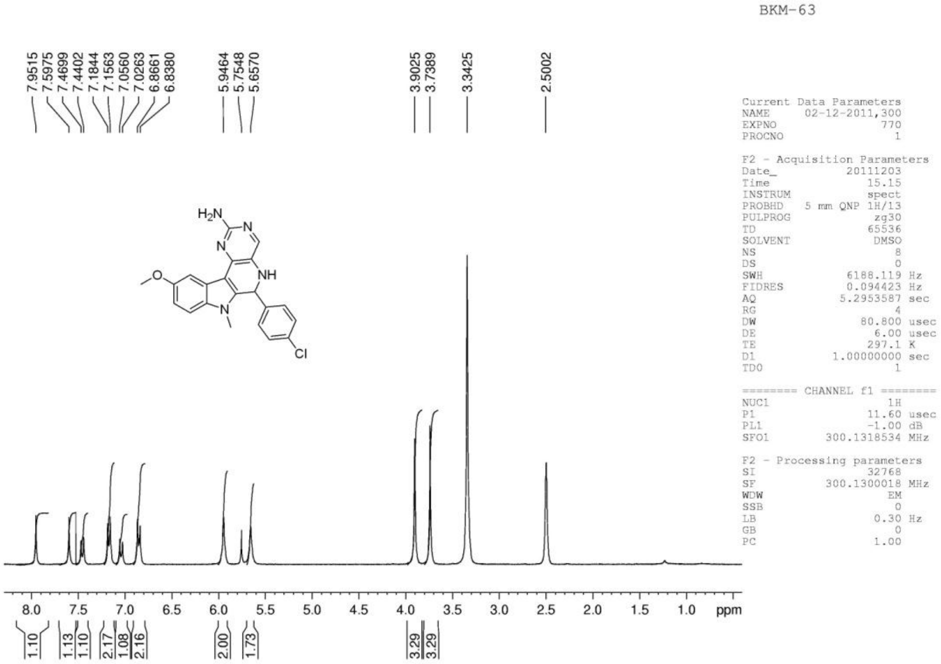

Figure 47: ^{1}H NMR of **12a**.

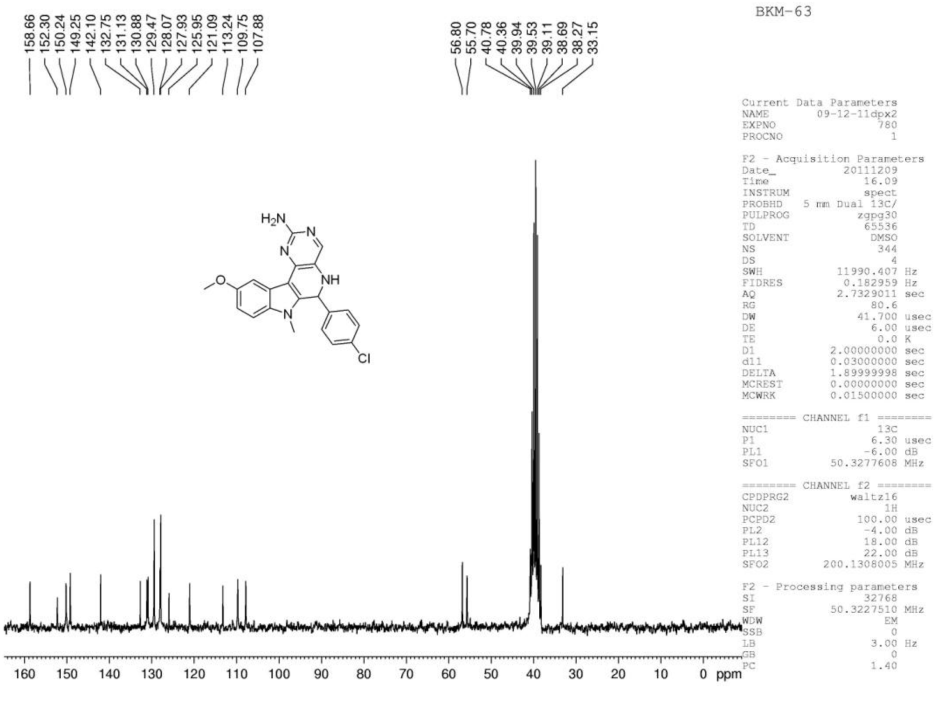

Figure 48: ^{13}C NMR of **12a**.

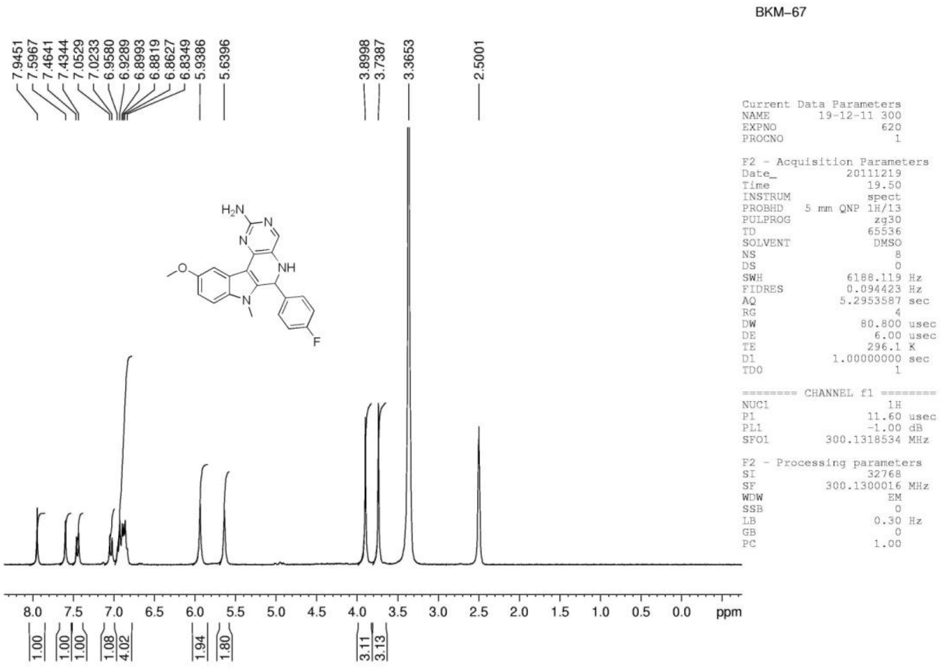

Figure 49: ^{1}H NMR of **12b**.

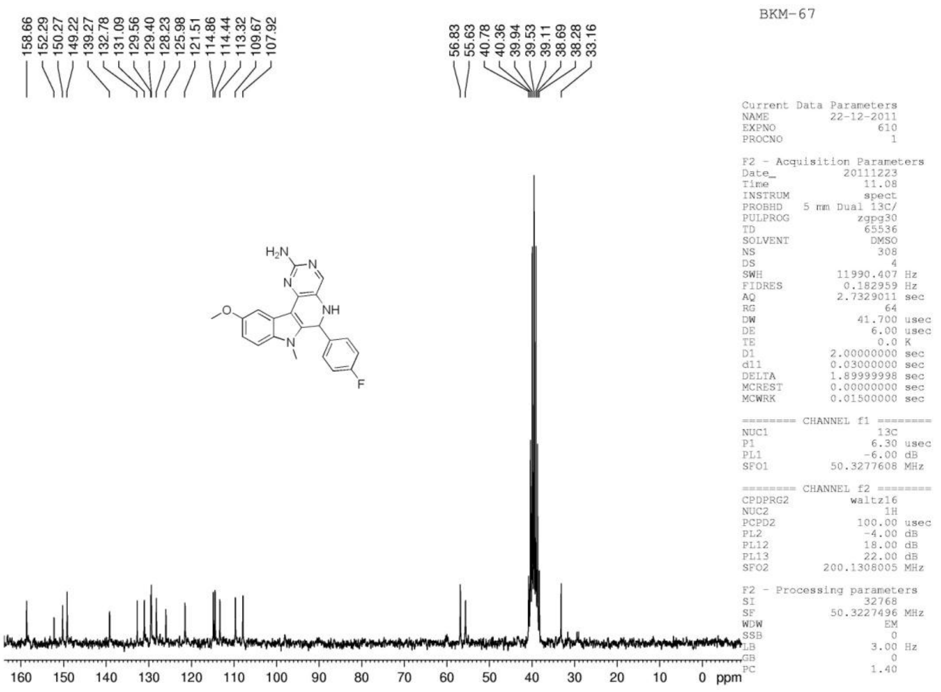

Figure 50: ^{13}C NMR of **12b**.

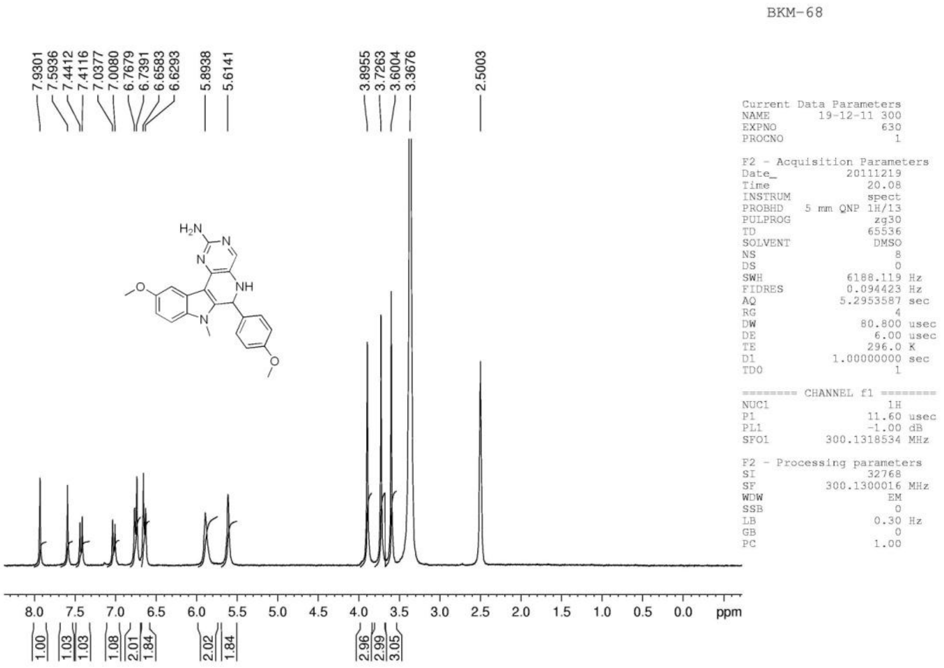

Figure 51: ^{1}H NMR of **12c**.

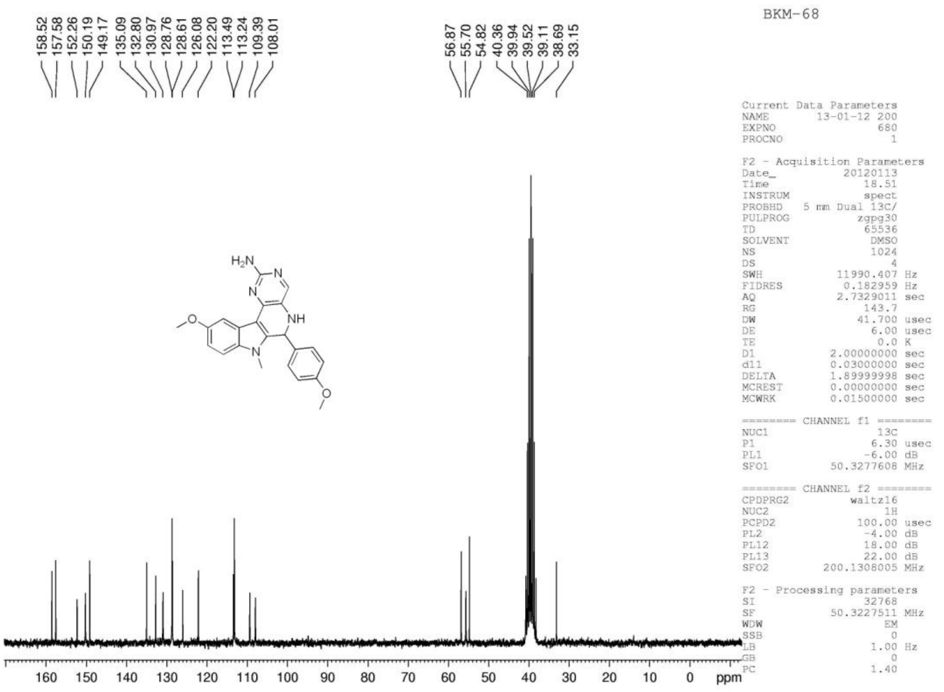

Figure 52: ^{13}C NMR of **12c**.

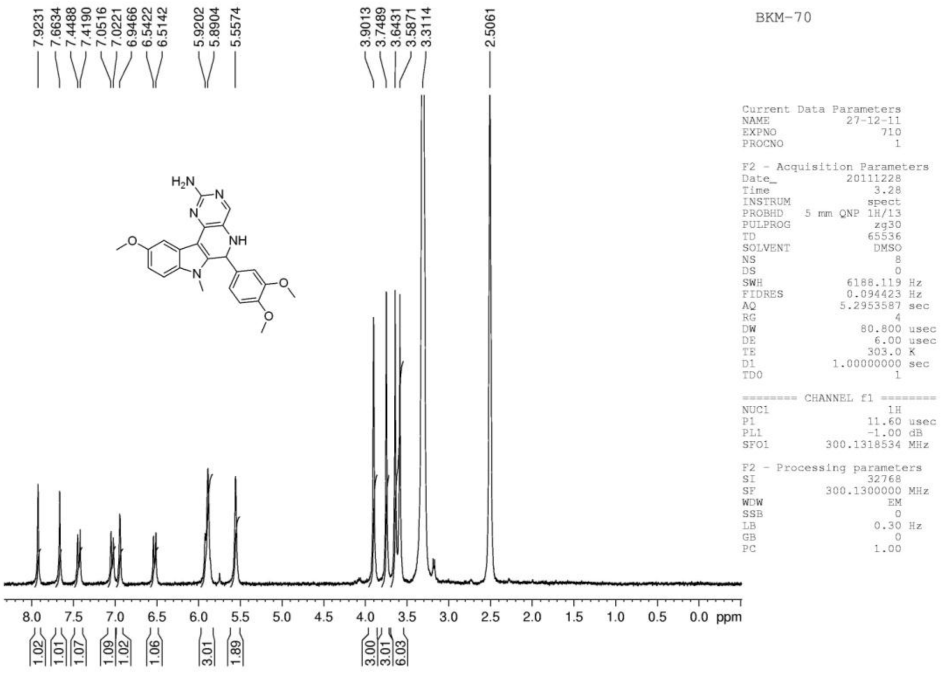

Figure 53: ^{1}H NMR of **12d**.

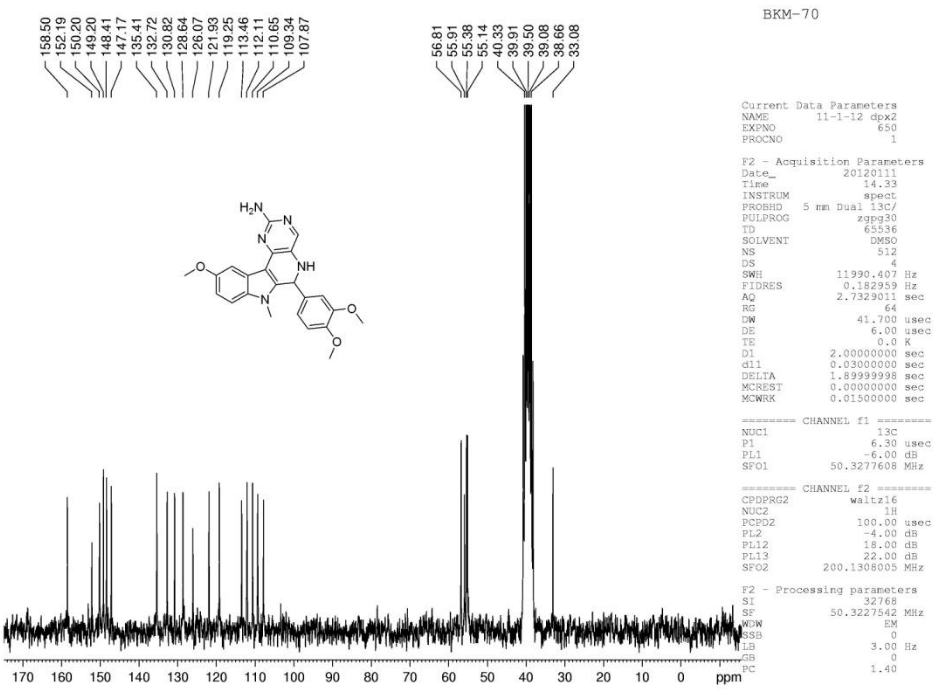

Figure 54: ^{13}C NMR of **12d**.

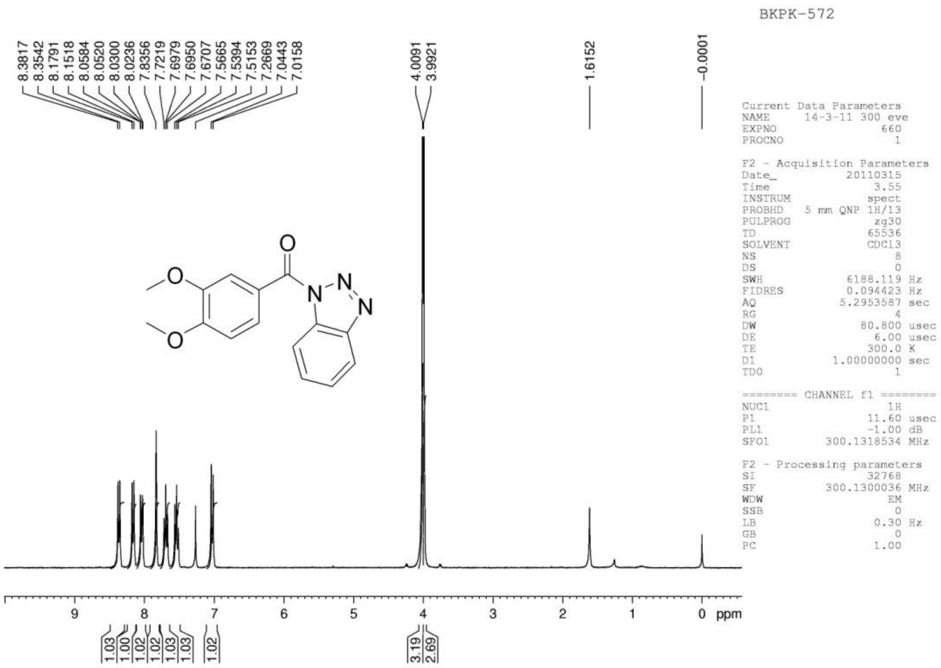

Figure 55: ^{1}H NMR of **14a**.

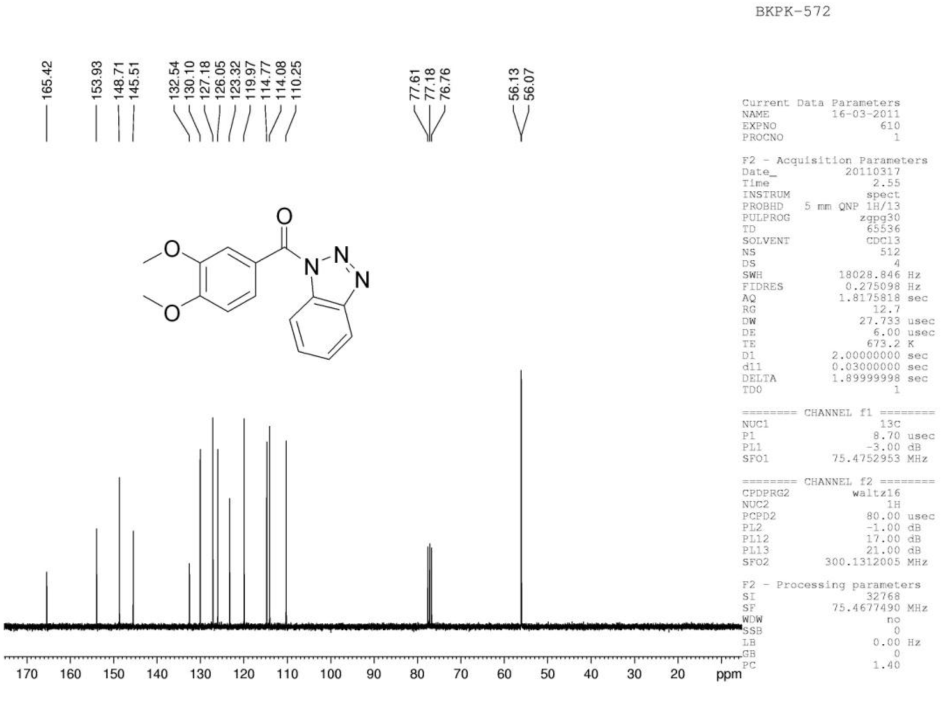

Figure 56: ^{13}C NMR of **14a**.

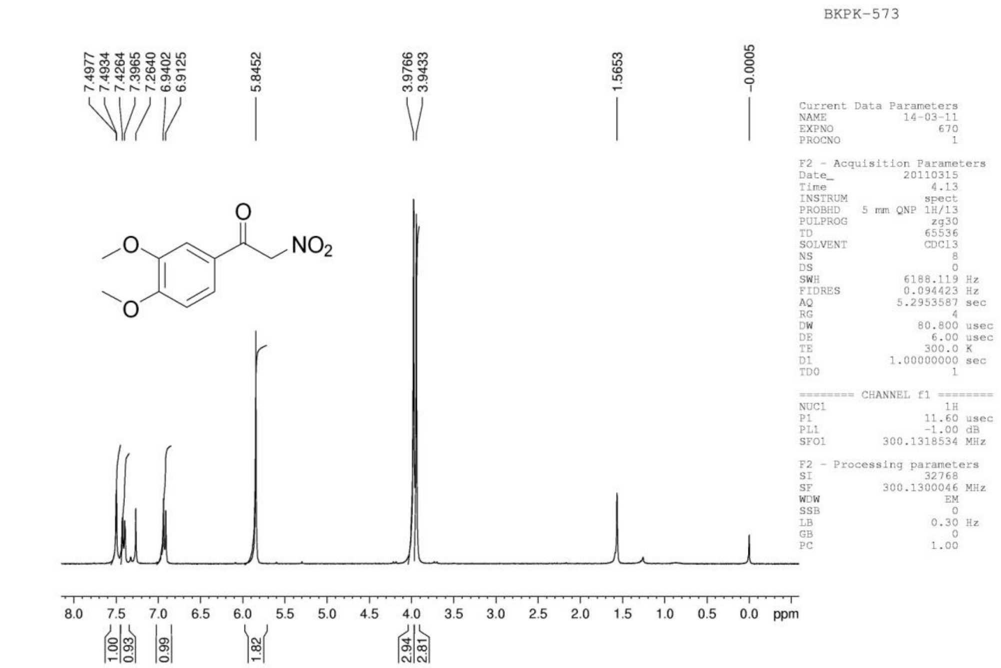

Figure 57: ^{1}H NMR of **15a**.

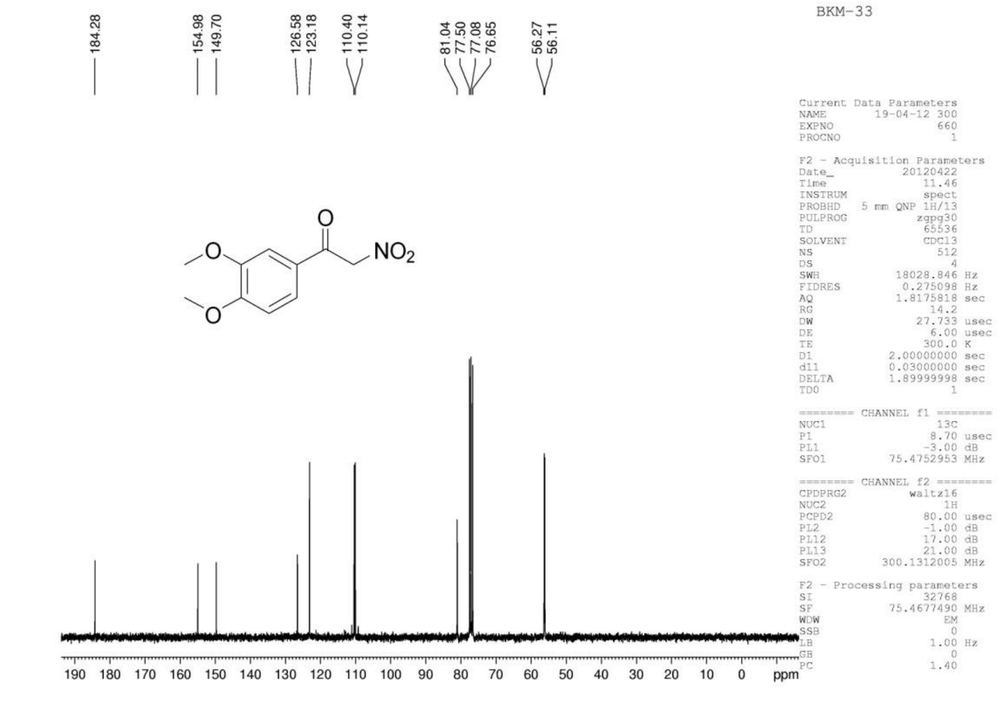

Figure 58: ^{13}C NMR of **15a**.

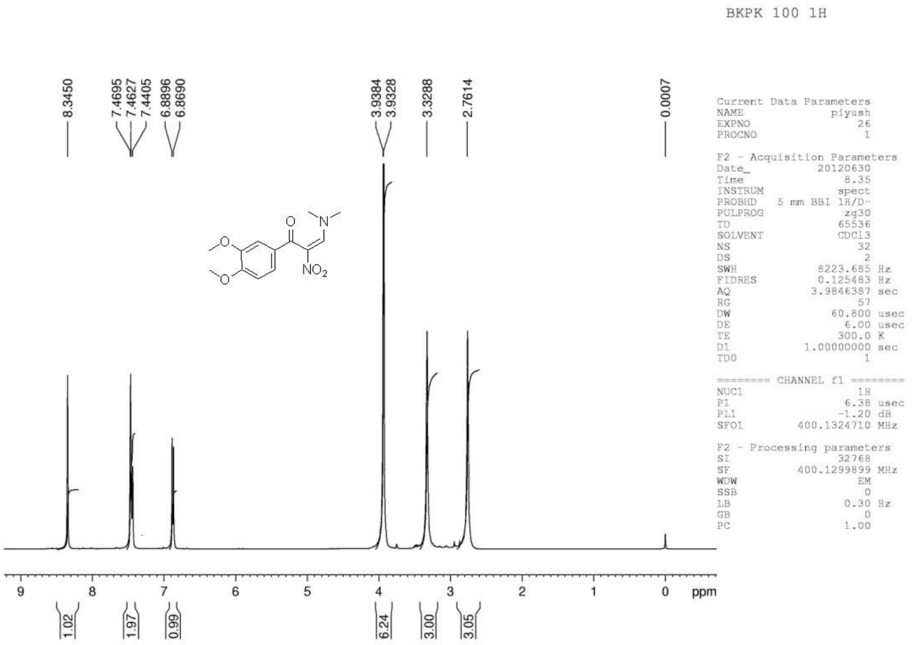

Figure 59: ^{1}H NMR of **16a**.

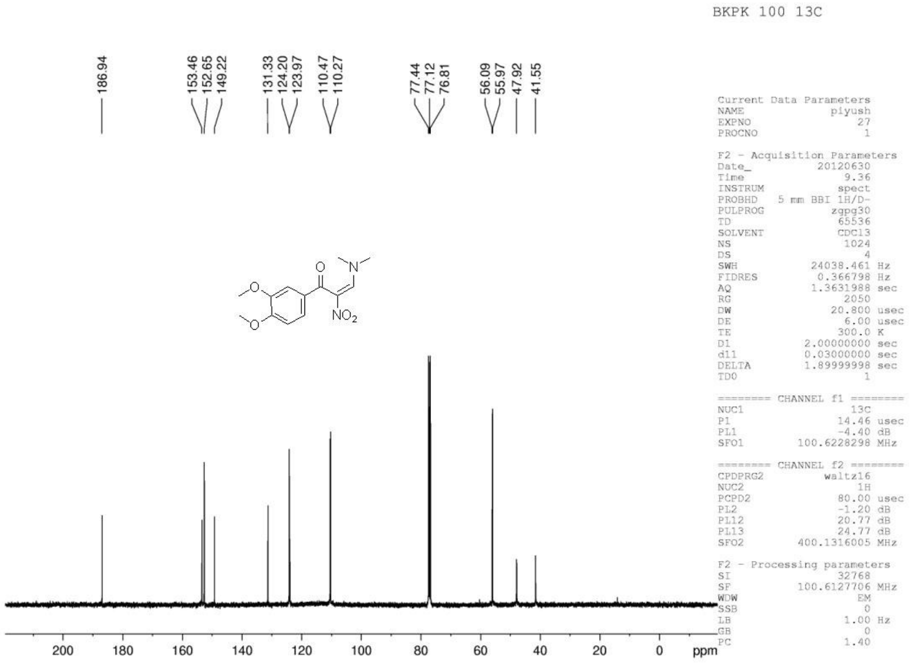

Figure 60: ^{13}C NMR of **16a**.

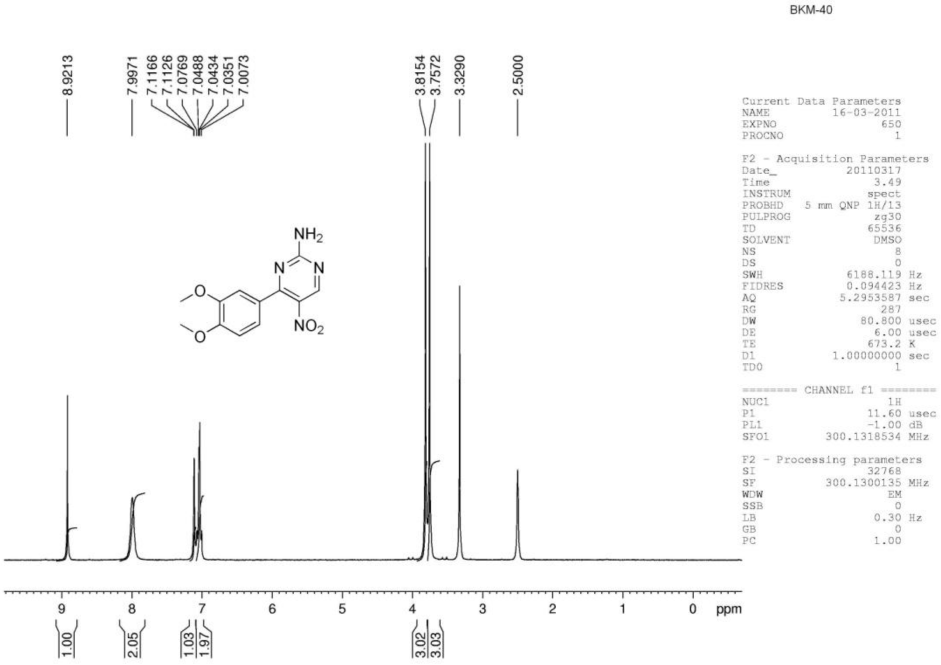

Figure 61: ^{1}H NMR of **17a**.

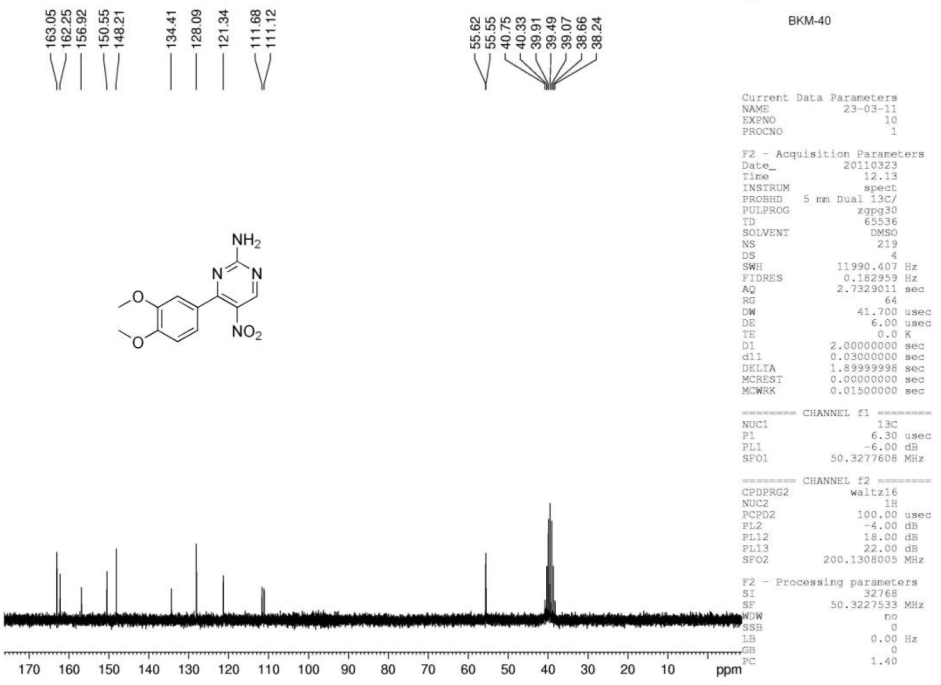

Figure 62: ^{13}C NMR of **17a**.

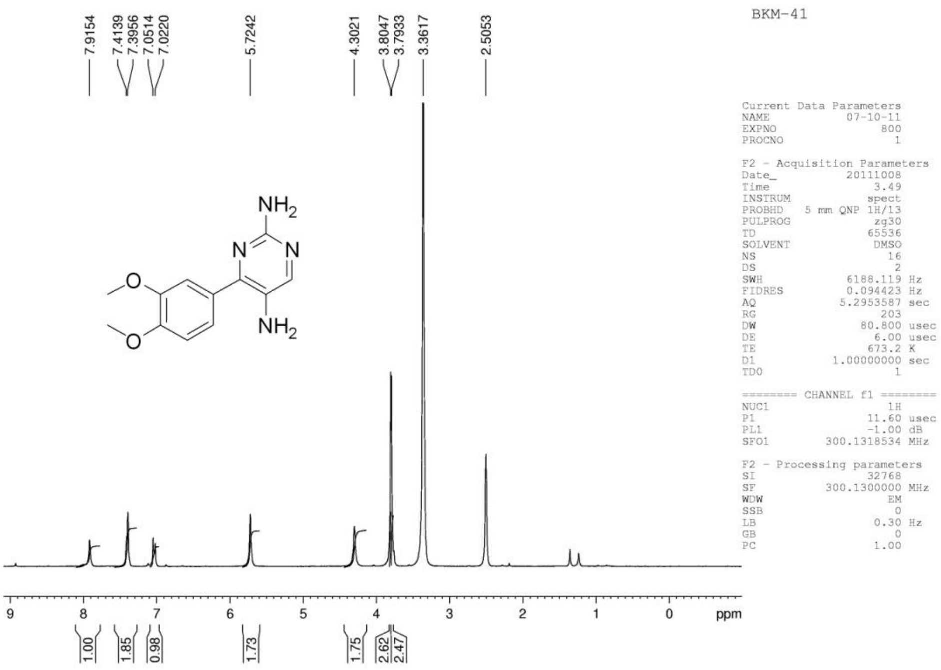

Figure 63: ^{1}H NMR of **18a**.

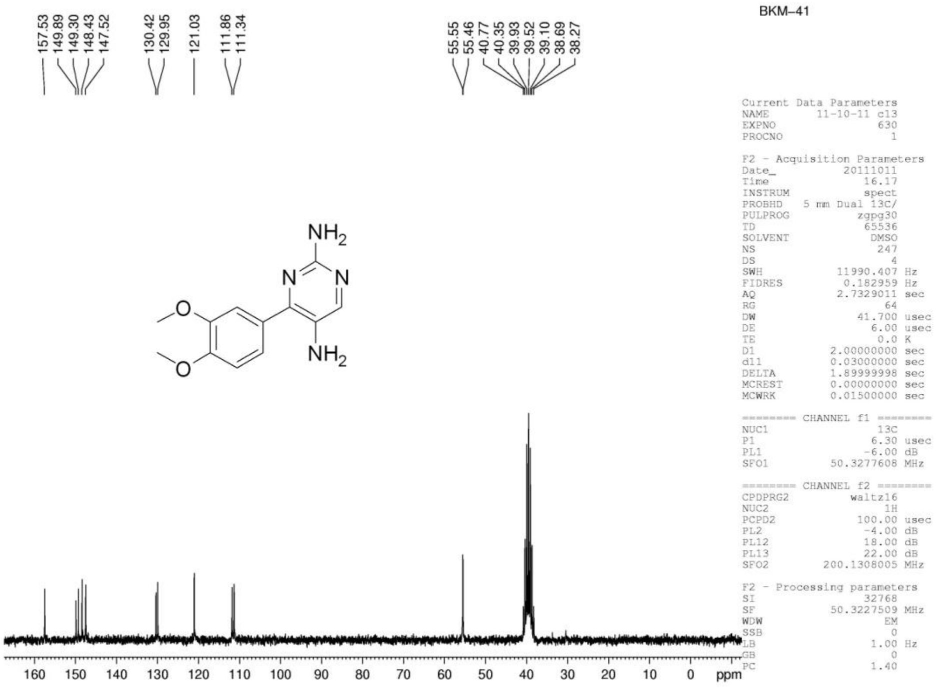

Figure 64: ^{13}C NMR of **18a**.

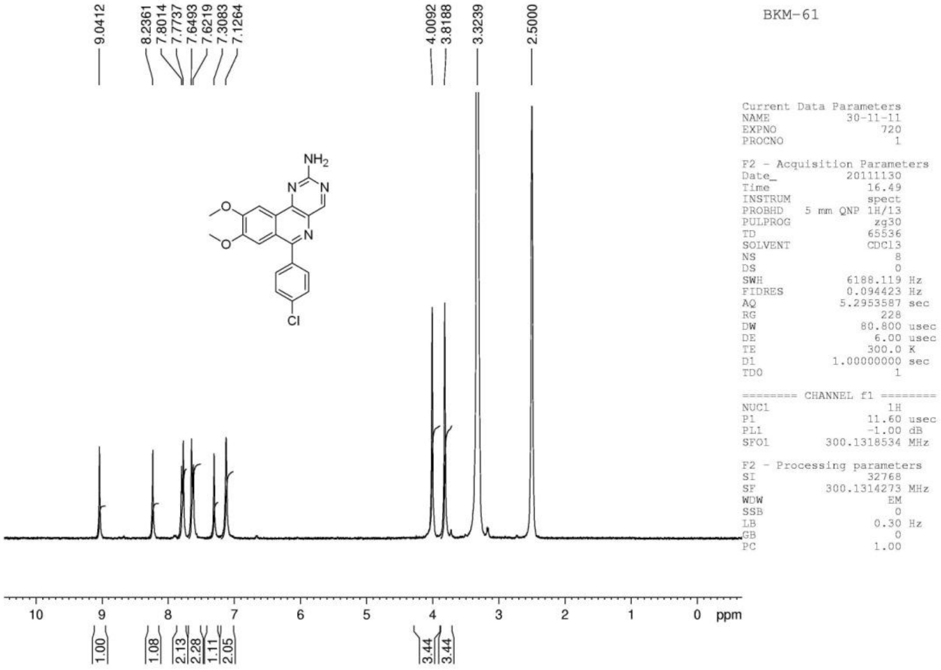

Figure 65: ^{1}H NMR of **20c**.

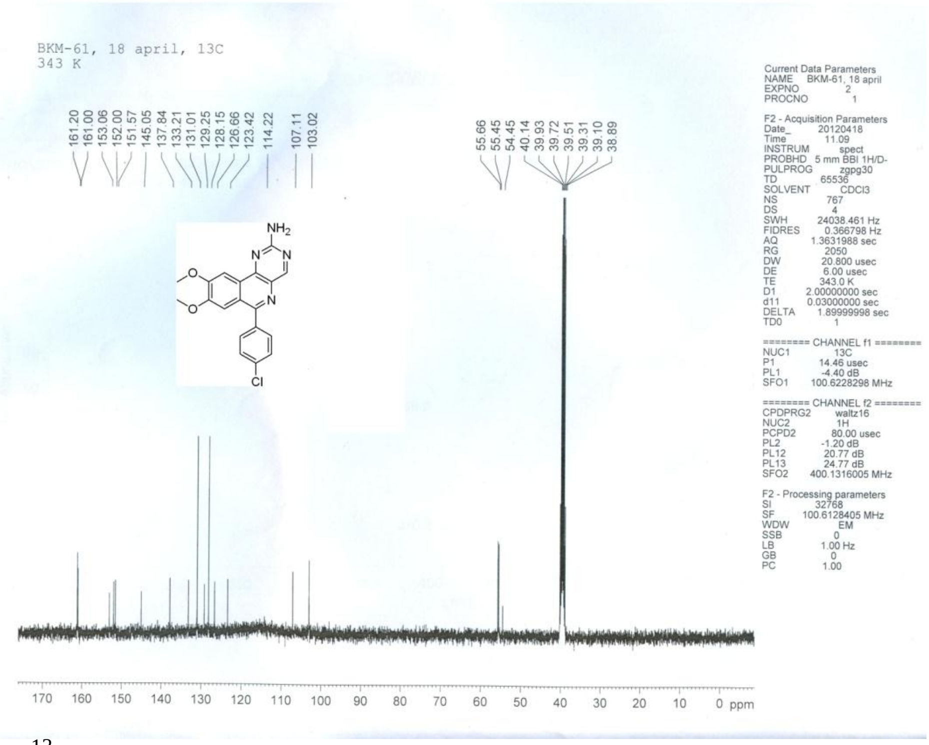

Figure 66: ^{13}C NMR of **20c**.

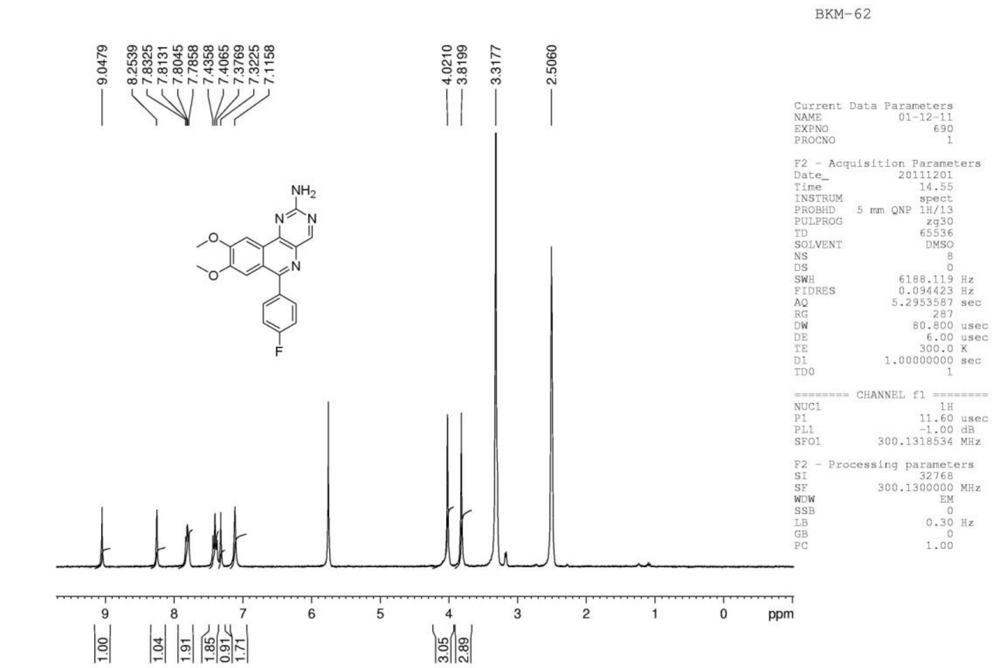

Figure 67: ^{1}H NMR of **20a**.

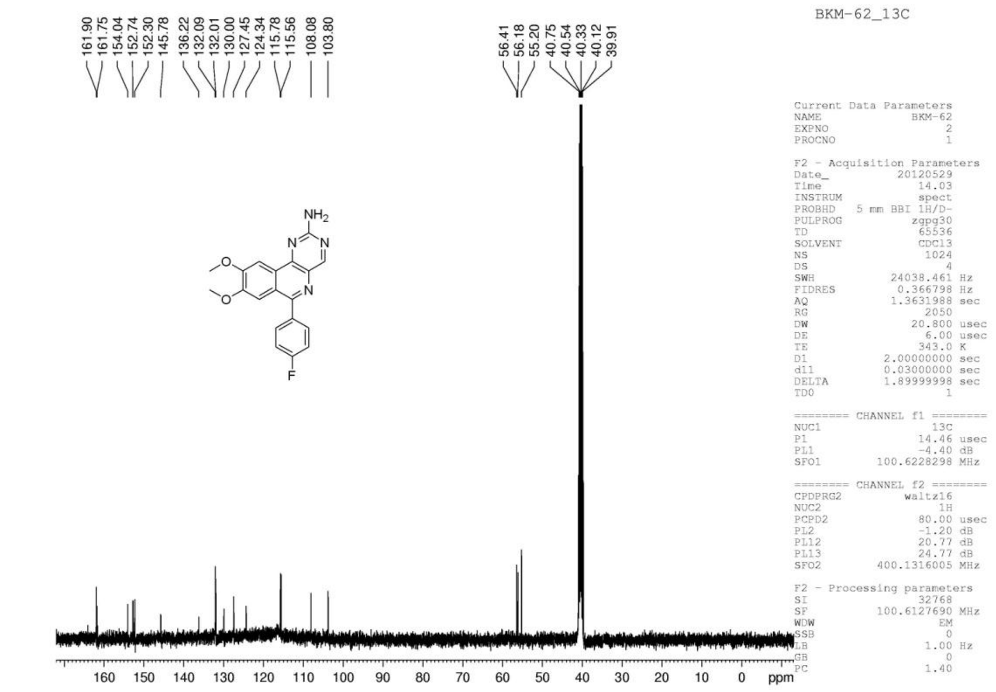

Figure 68: ^{13}C NMR of **20a**.

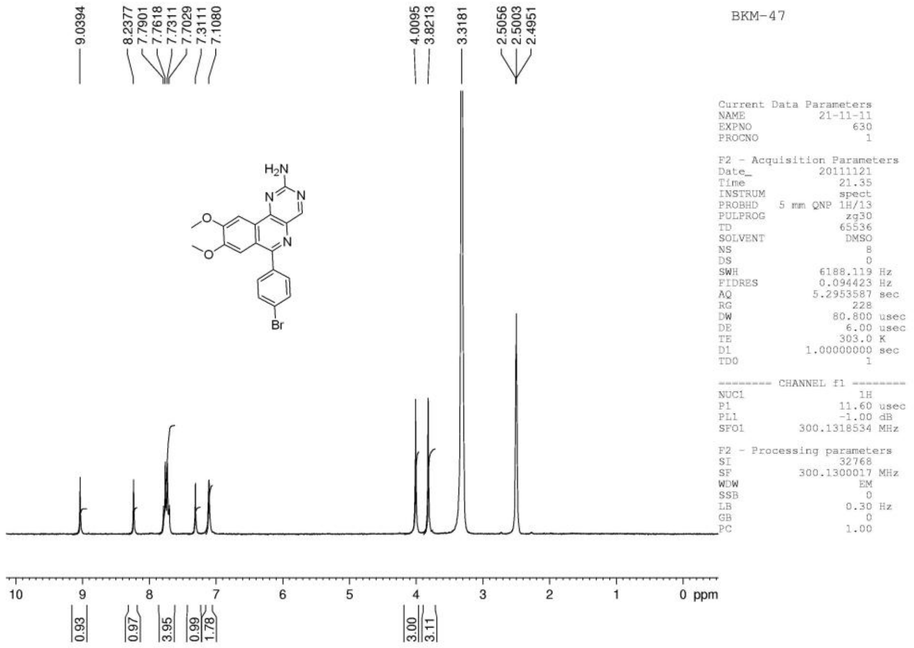

Figure 69: ^{1}H NMR of **20b**.

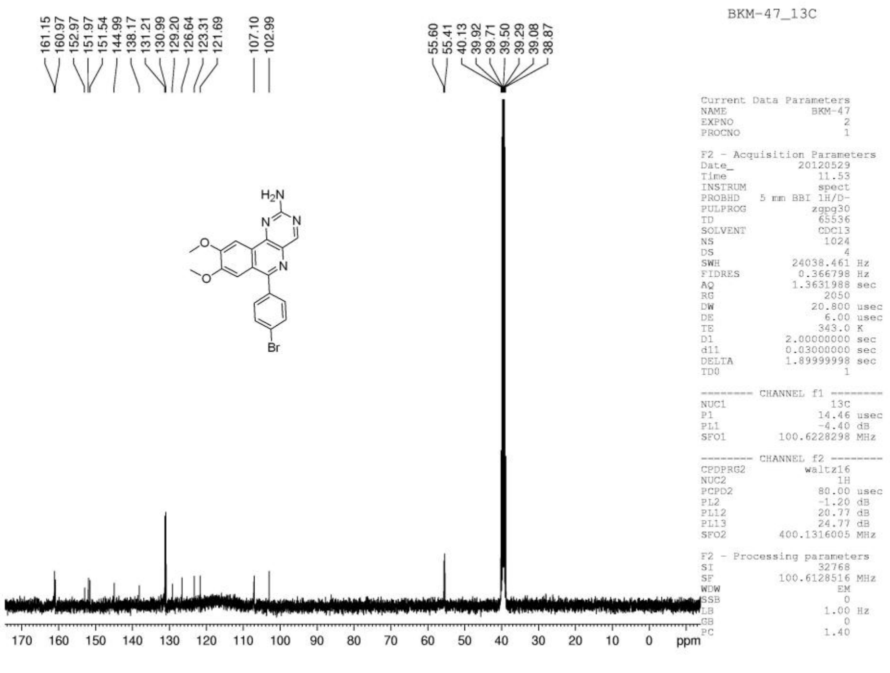

Figure 70: ^{13}C NMR of **20b**.

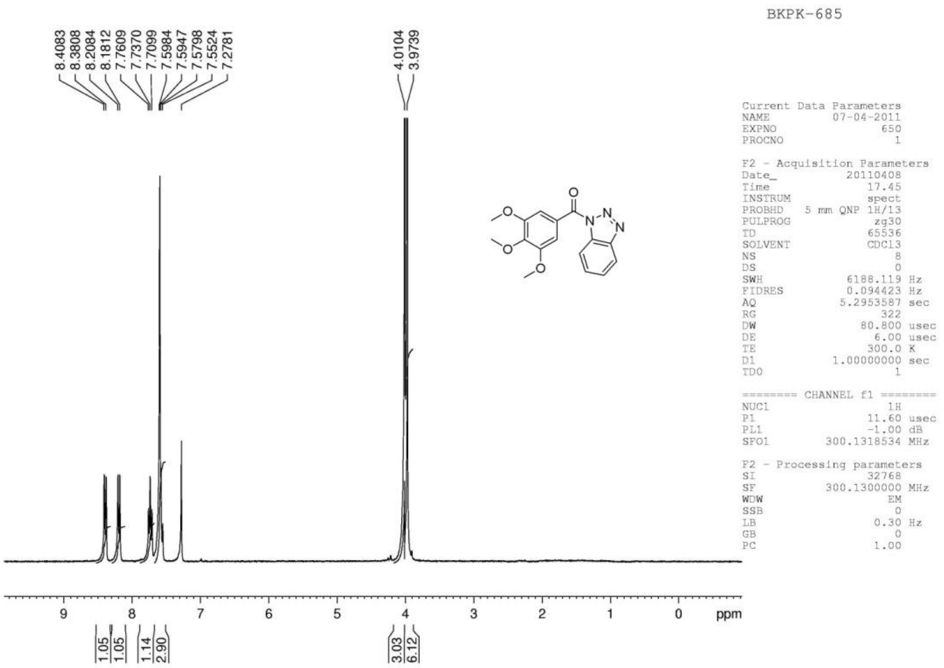

Figure 71: ^{1}H NMR of **14b**.

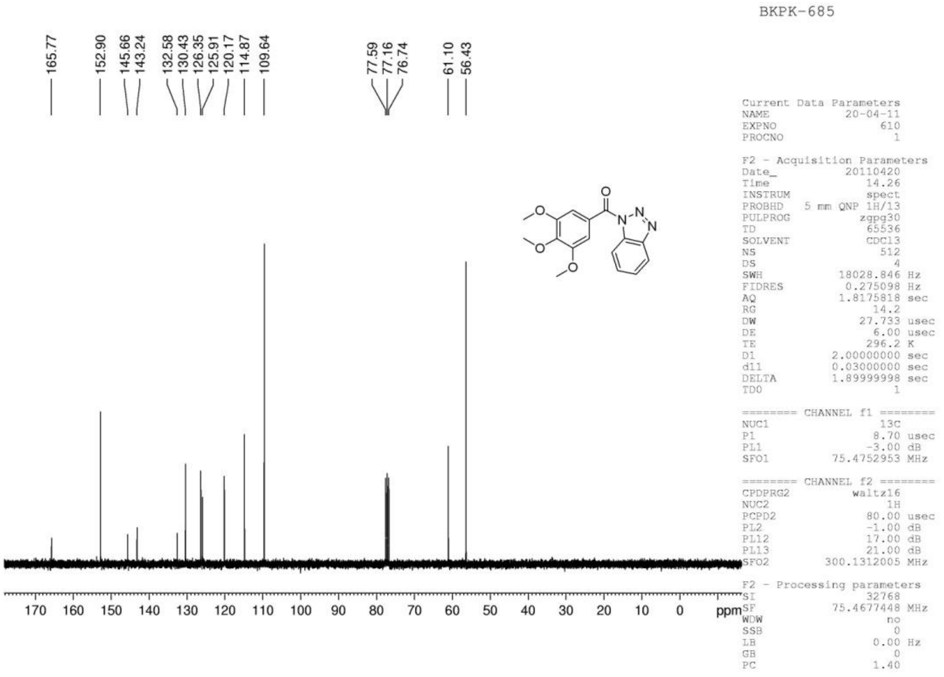

Figure 72: ^{13}C NMR of **14b**.

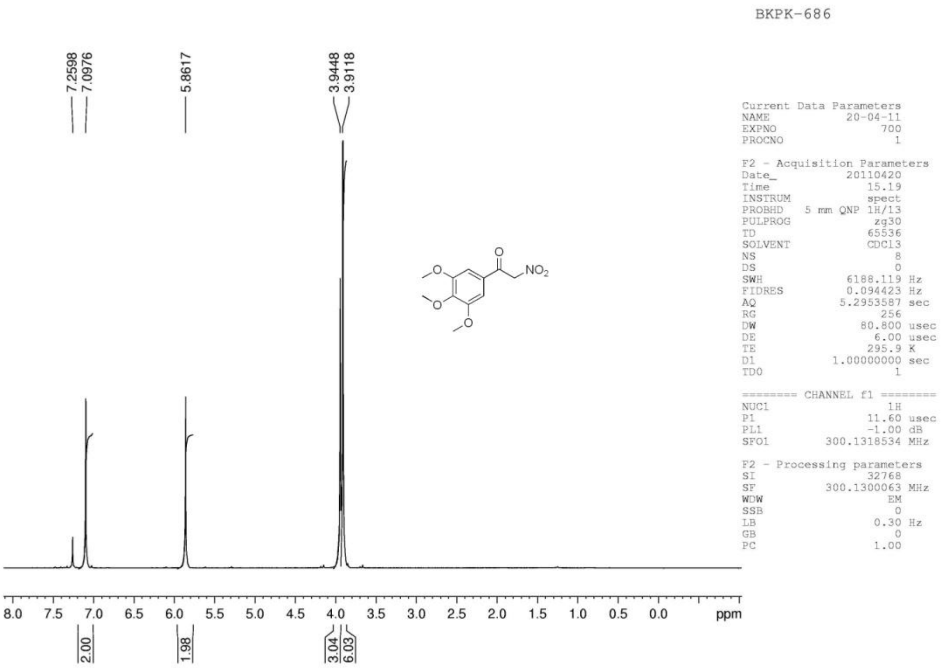

Figure 73: ^{1}H NMR of **15b**.

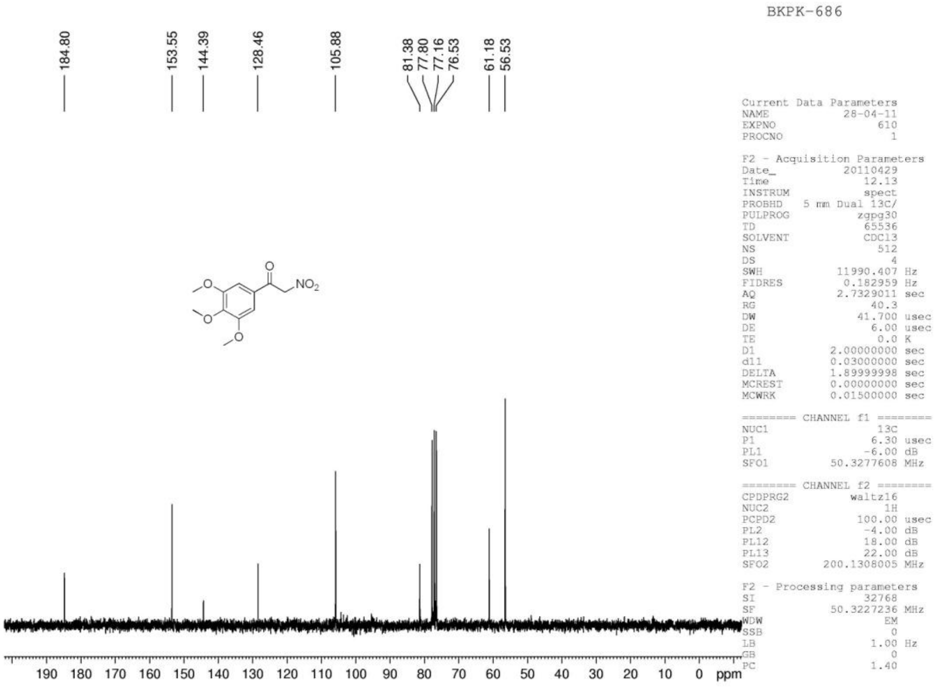

Figure 74: ^{13}C NMR of **15b**.

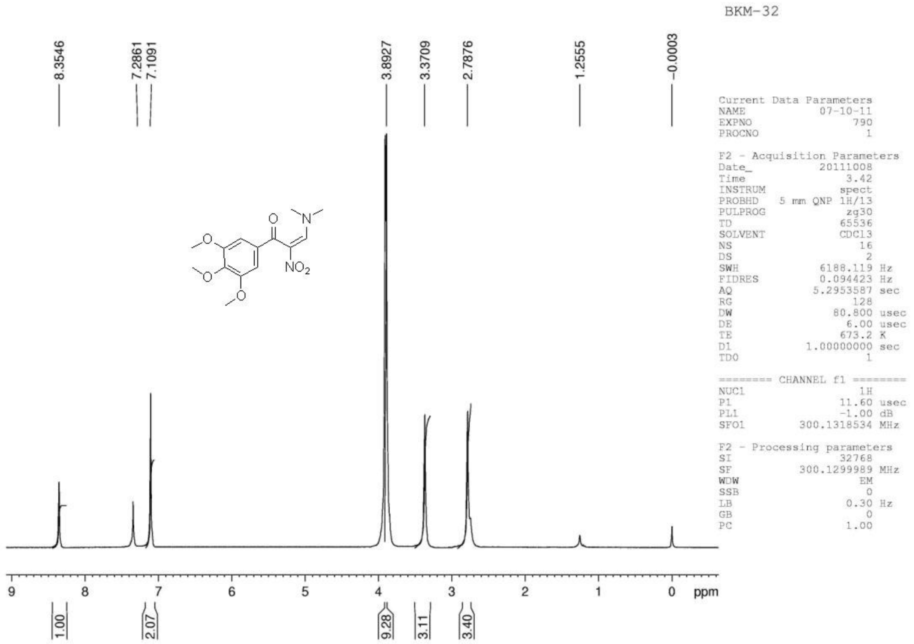

Figure 75: ^{1}H NMR of **16b**.

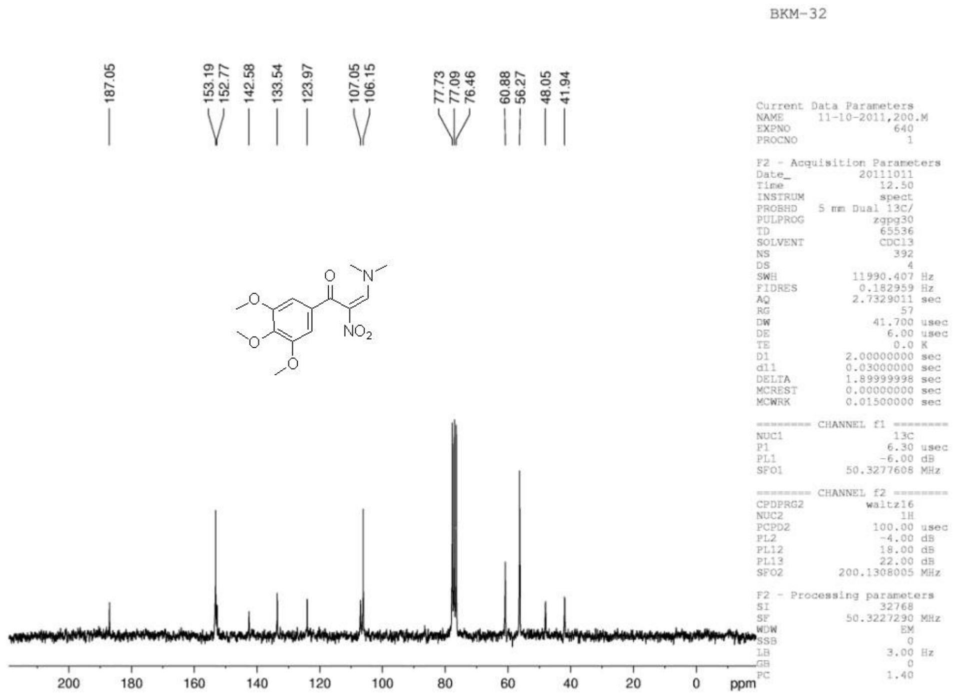

Figure 76: ^{13}C NMR of **16b**.

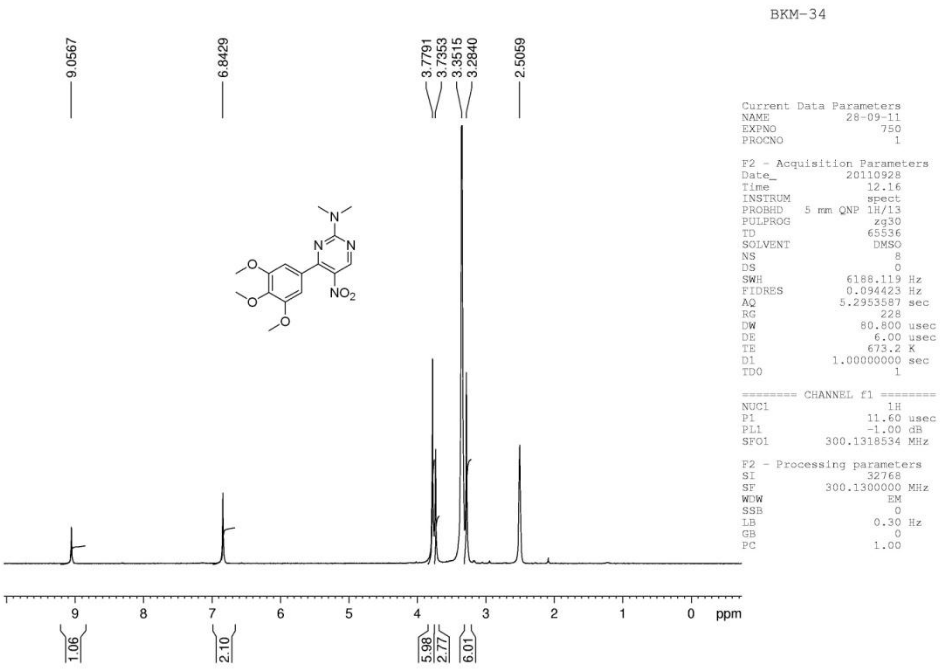

Figure 77: ^{1}H NMR of **17c**.

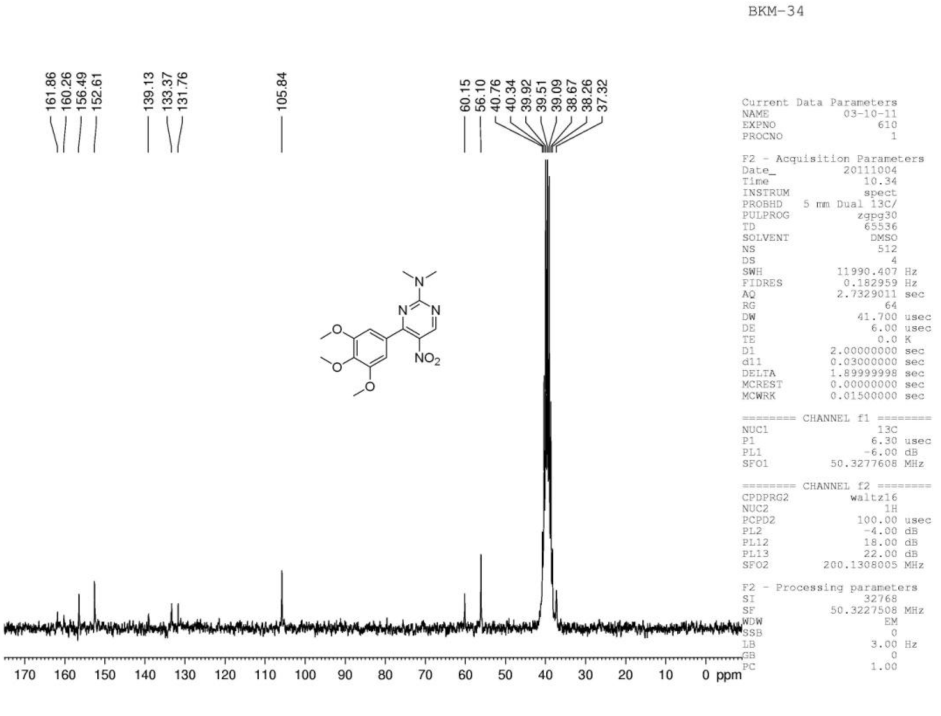

Figure 78: ^{13}C NMR of **17c**.

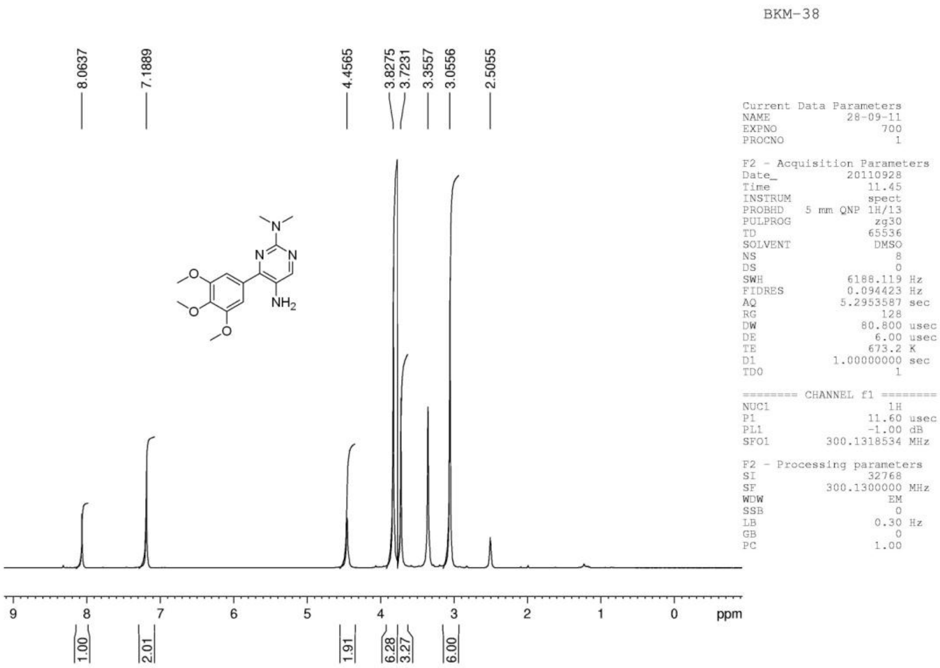

Figure 79: ^{1}H NMR of **18c**.

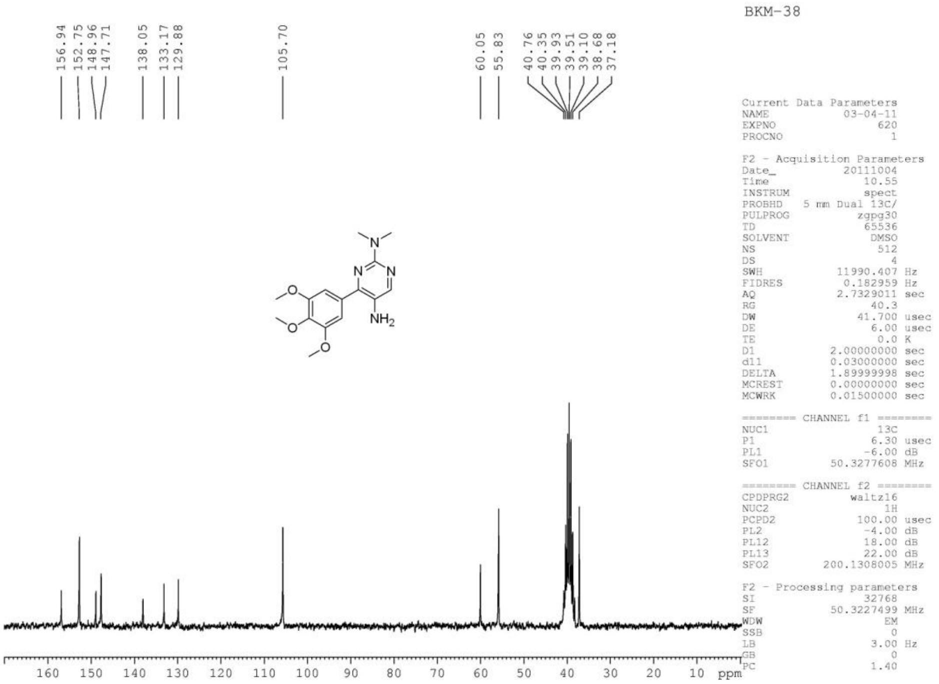

Figure 80: ^{13}C NMR of **18c**.

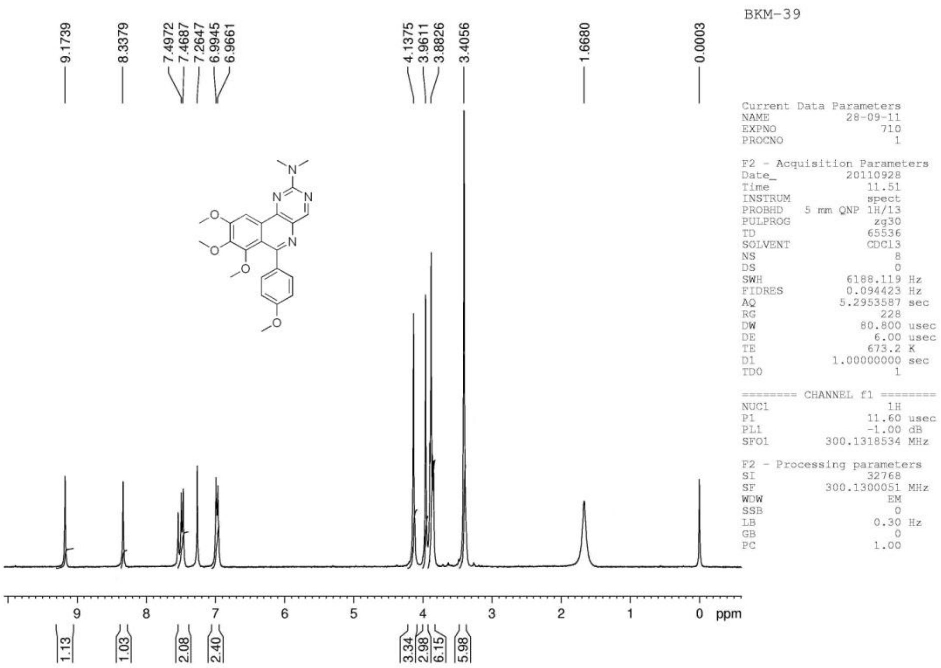

Figure 81: ^{1}H NMR of **20l**.

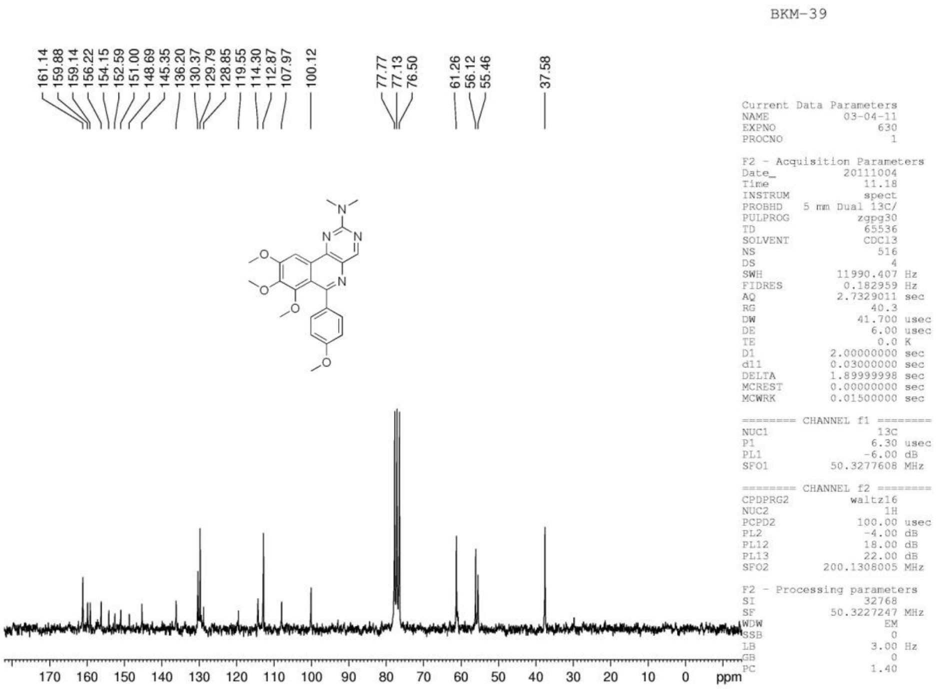

Figure 82: ^{13}C NMR of **20l**.

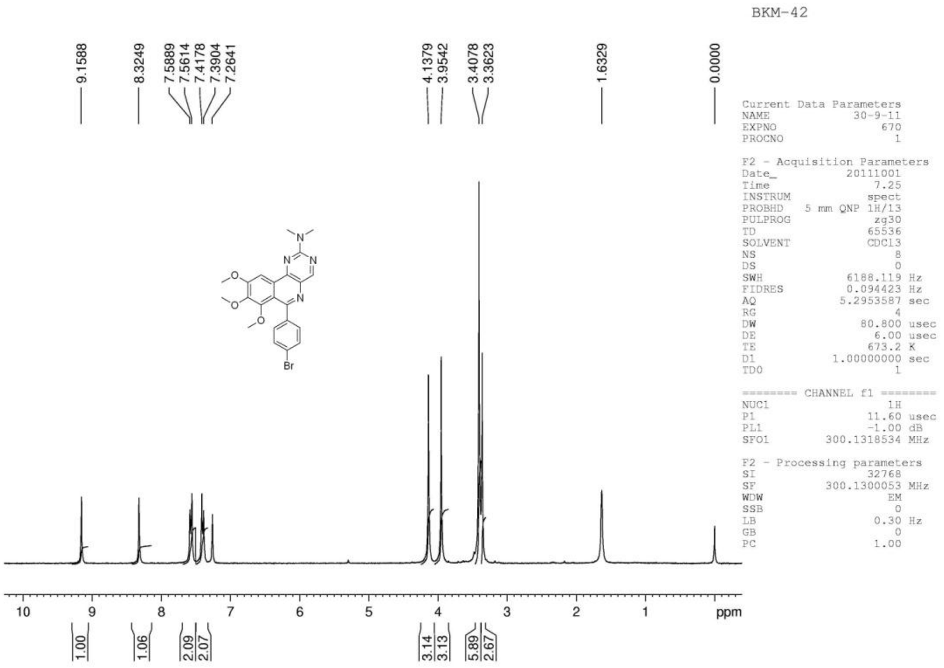

Figure 83: ^{1}H NMR of **20k**.

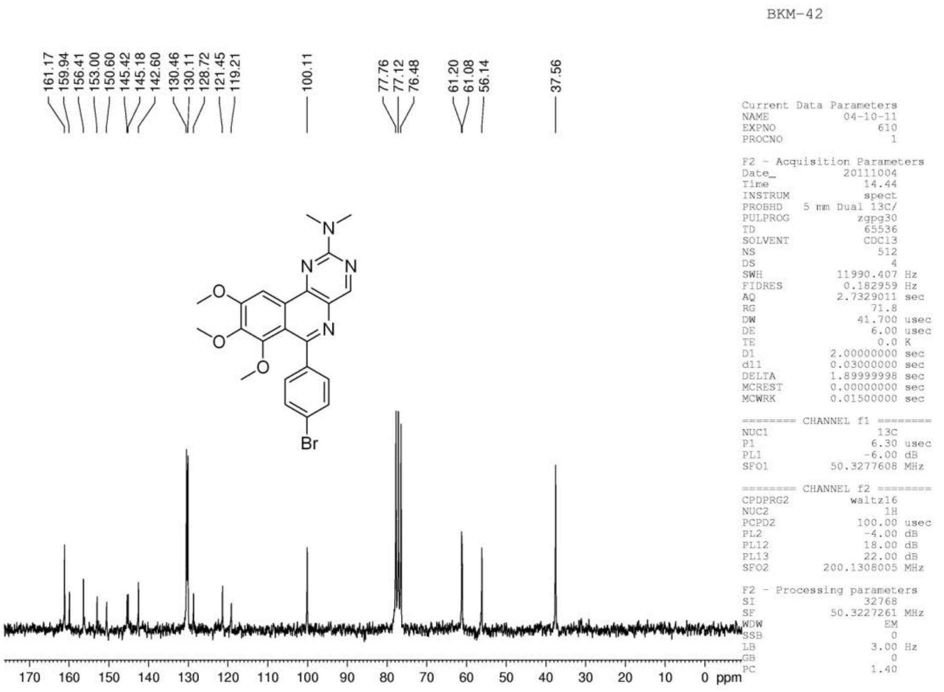

Figure 84: ^{13}C NMR of **20k**.

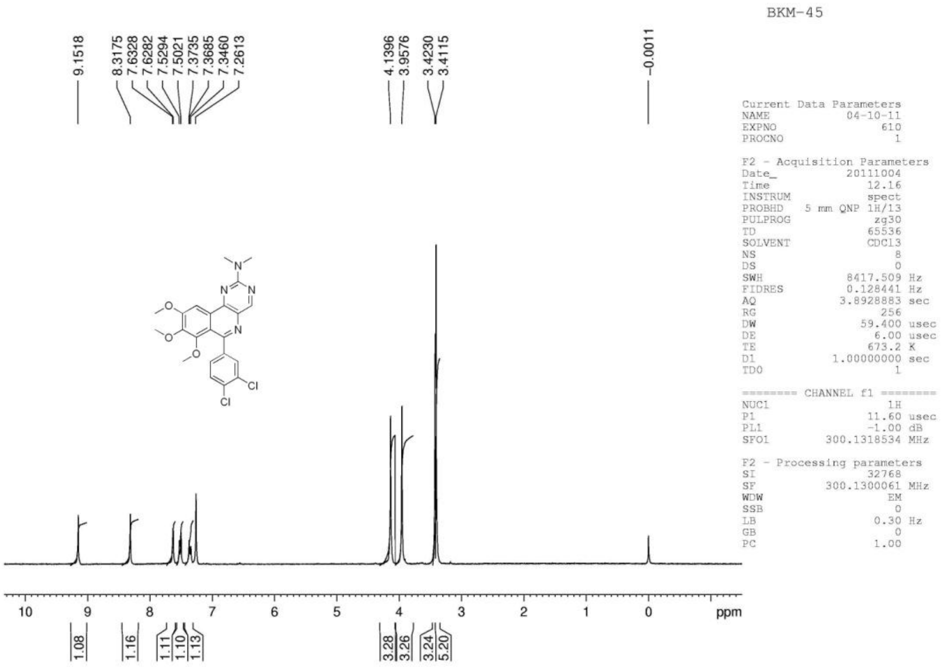

Figure 85: ^{1}H NMR of **20j**.

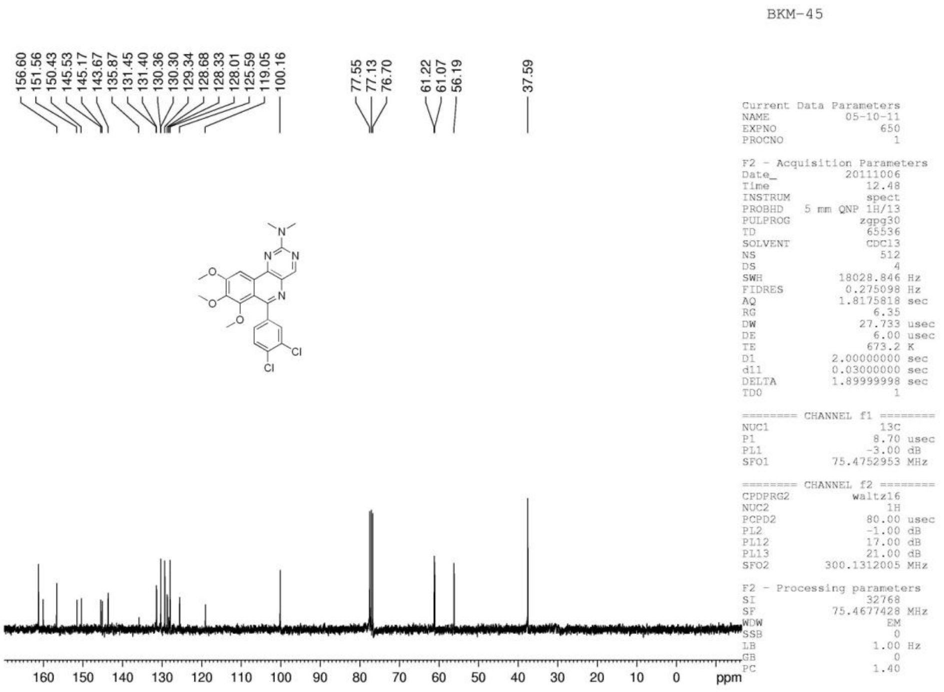

Figure 86: ^{13}C NMR of **20j**.

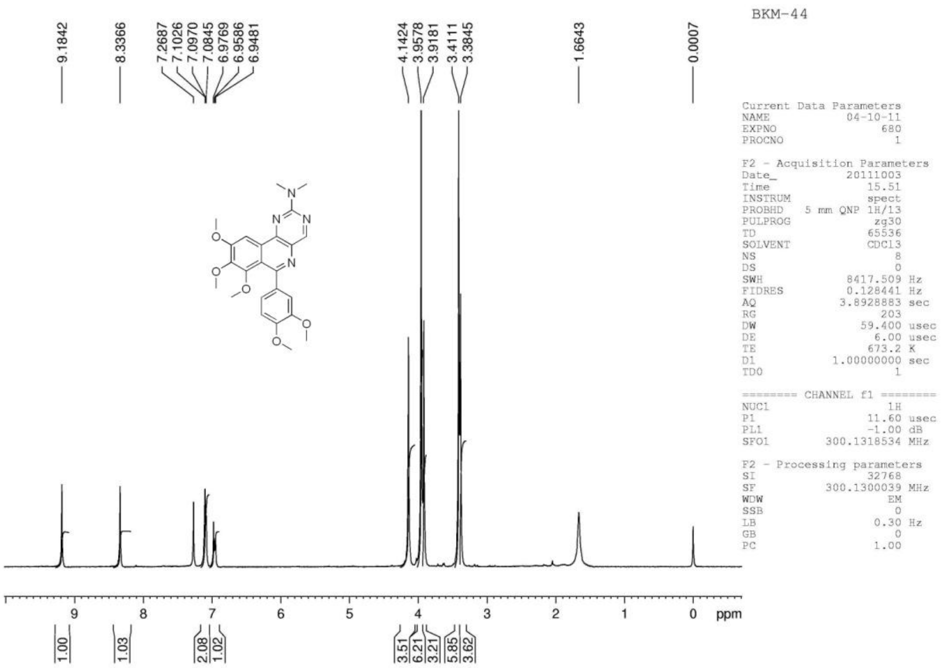

Figure 87: ^{1}H NMR of **20i**.

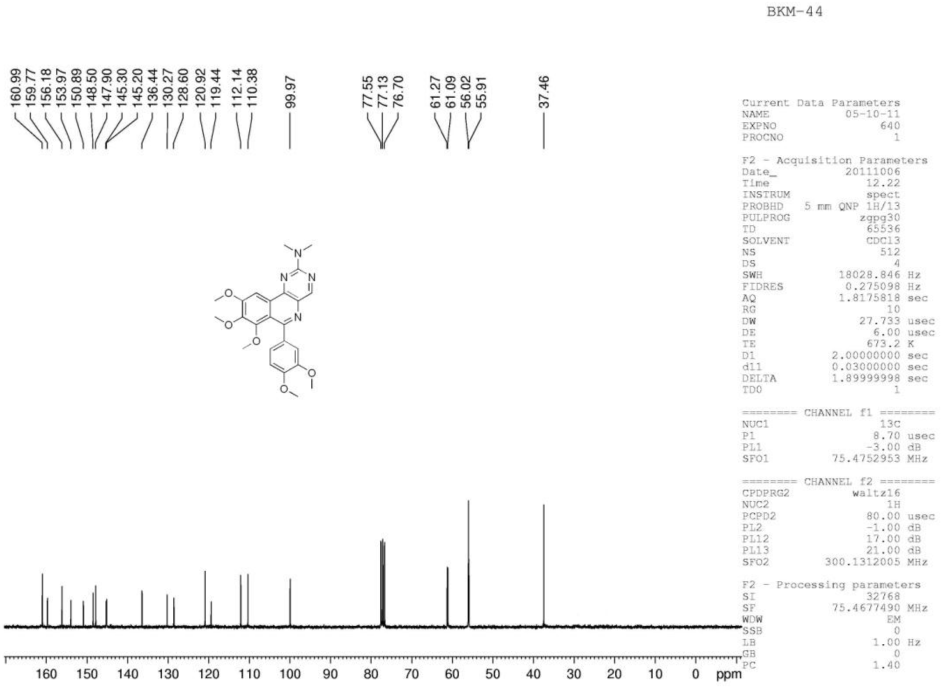

Figure 88: ^{13}C NMR of **20i**.

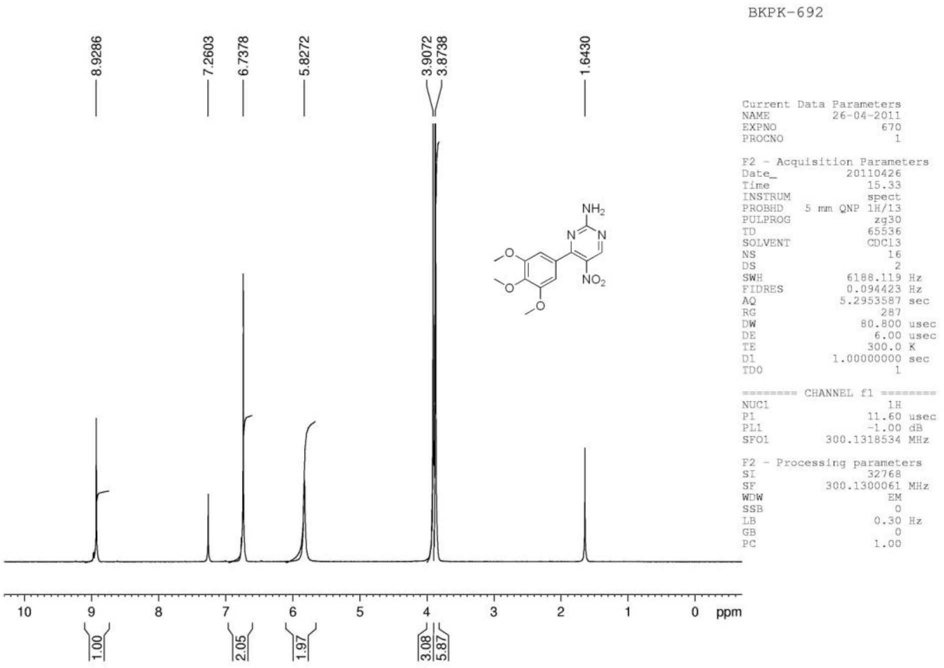

Figure 89: ^{1}H NMR of **17b**.

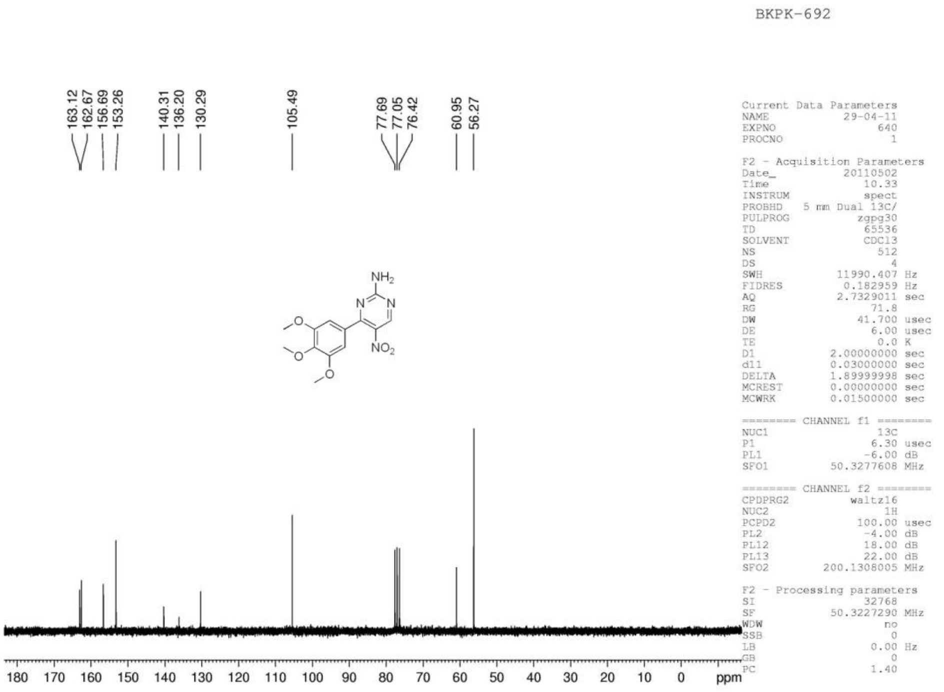

Figure 90: ^{13}C NMR of **17b**.

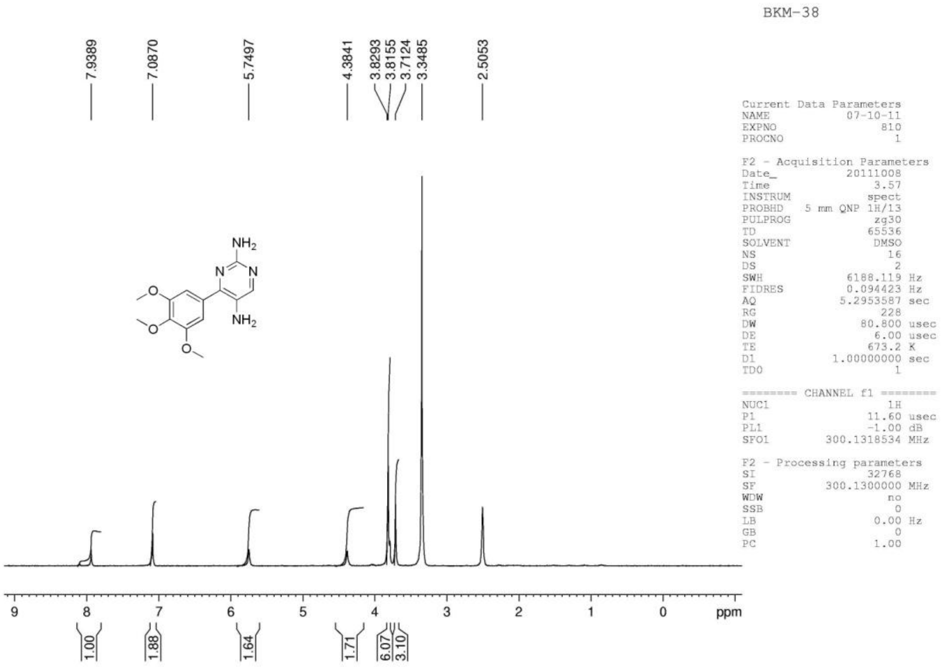

Figure 91: ^{1}H NMR of **18b**.

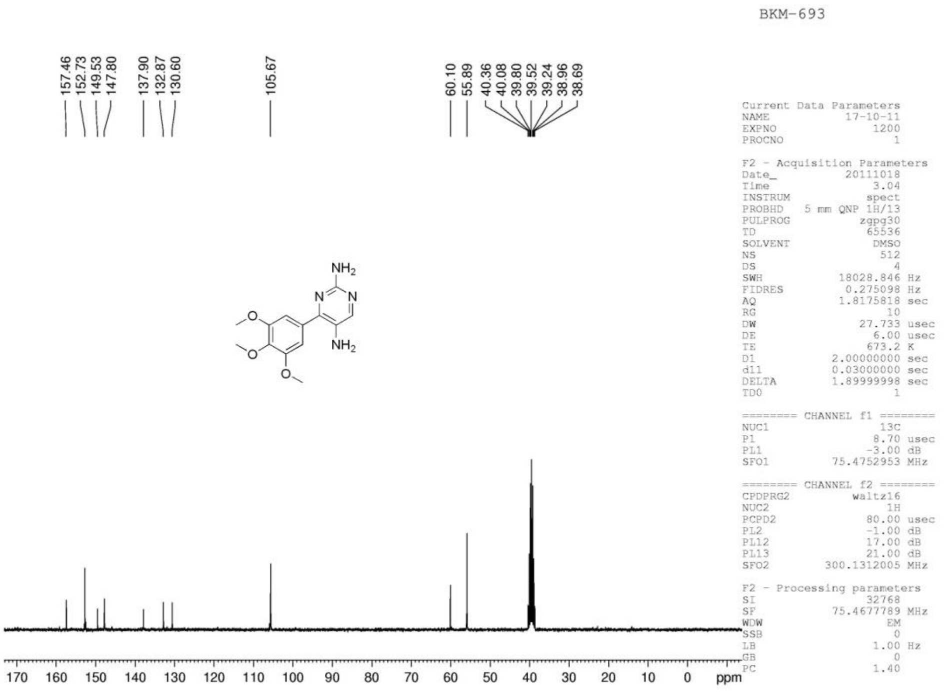

Figure 92: ^{13}C NMR of **18b**.

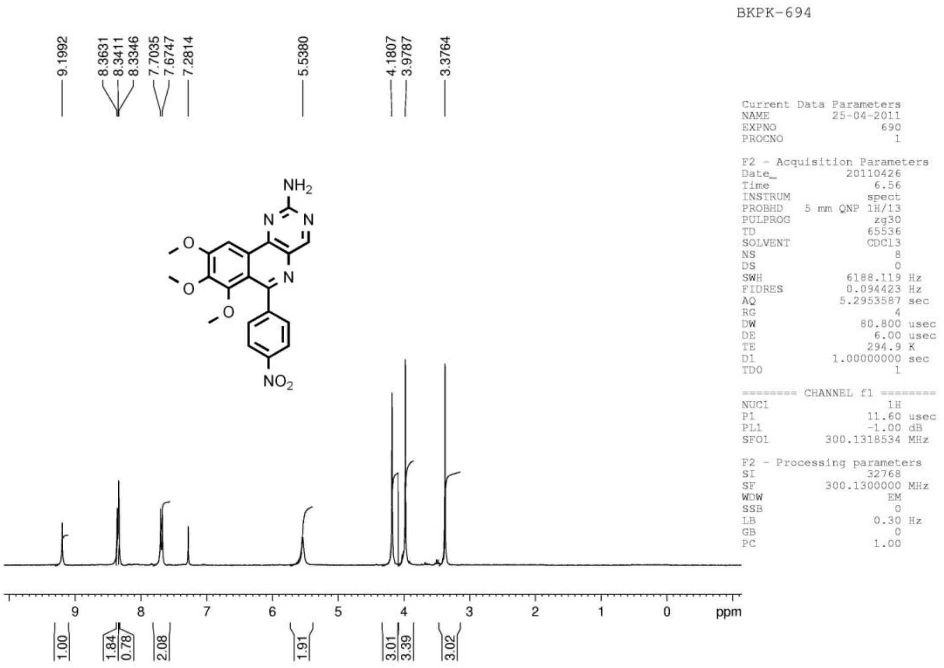

Figure 93: ^{1}H NMR of **20h**.

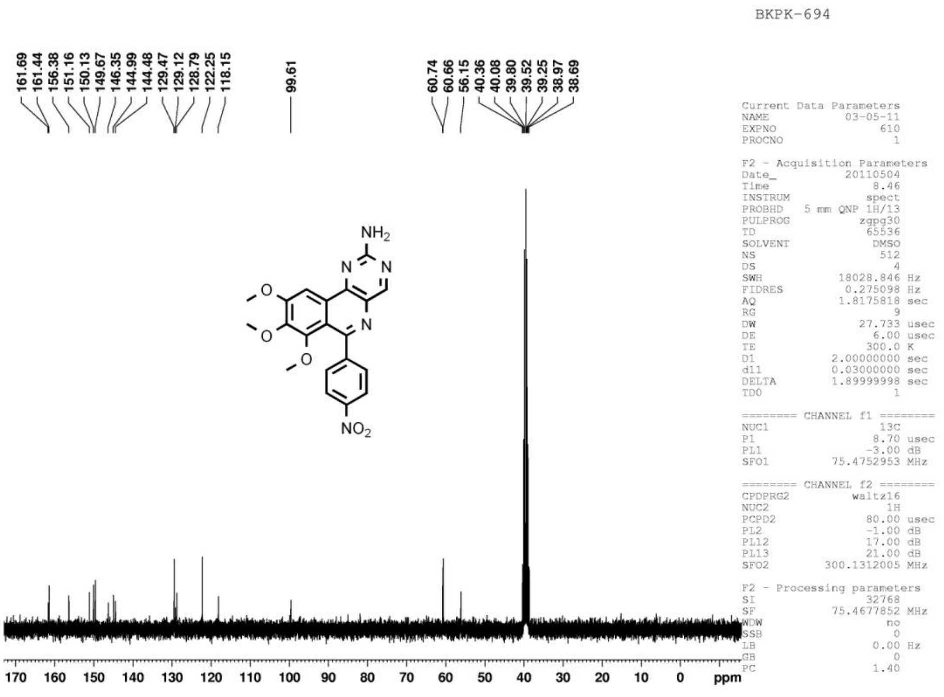

Figure 94: ^{13}C NMR of **20h**.

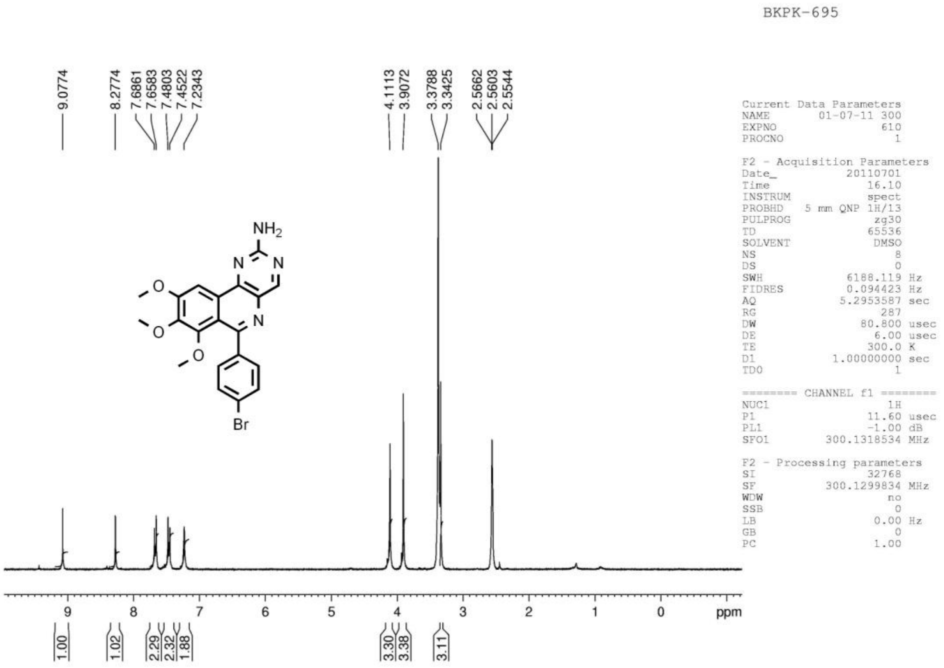

Figure 95: ^{1}H NMR of **20g**.

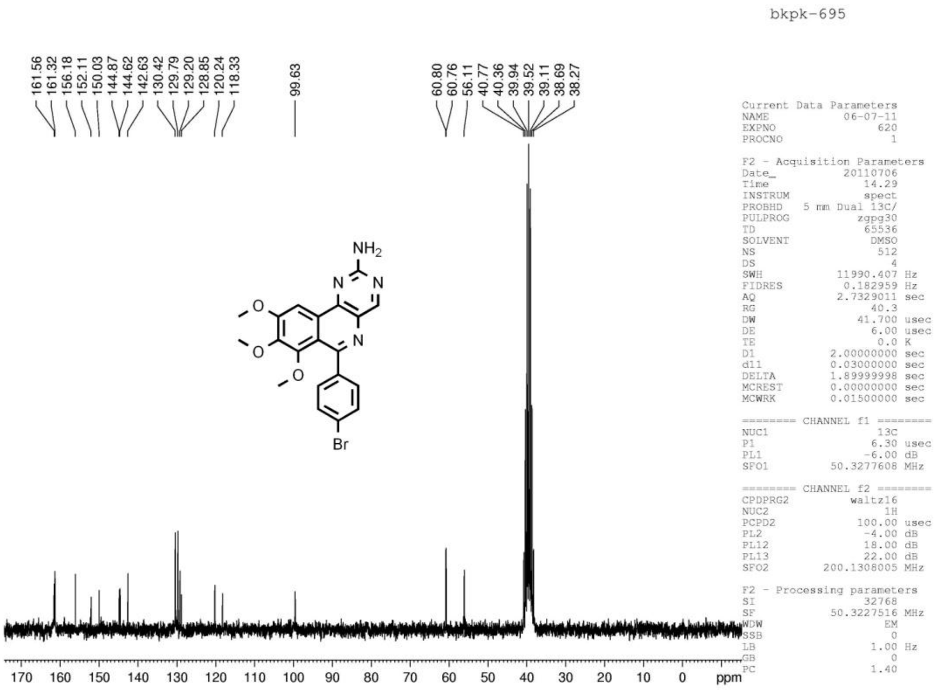

Figure 96: ^{13}C NMR of **20g**.

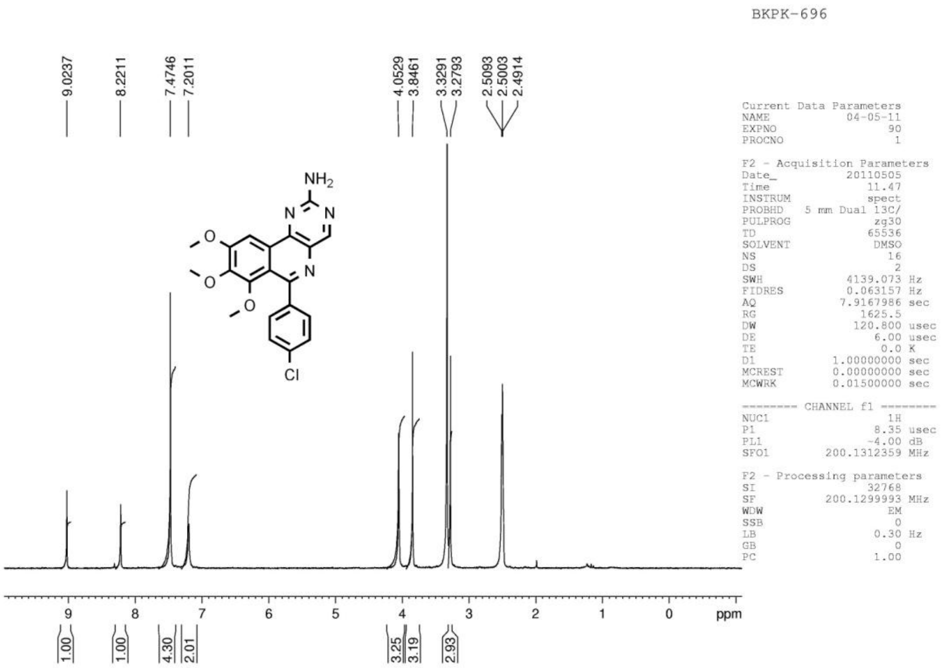

Figure 97: ^{1}H NMR of **20e**.

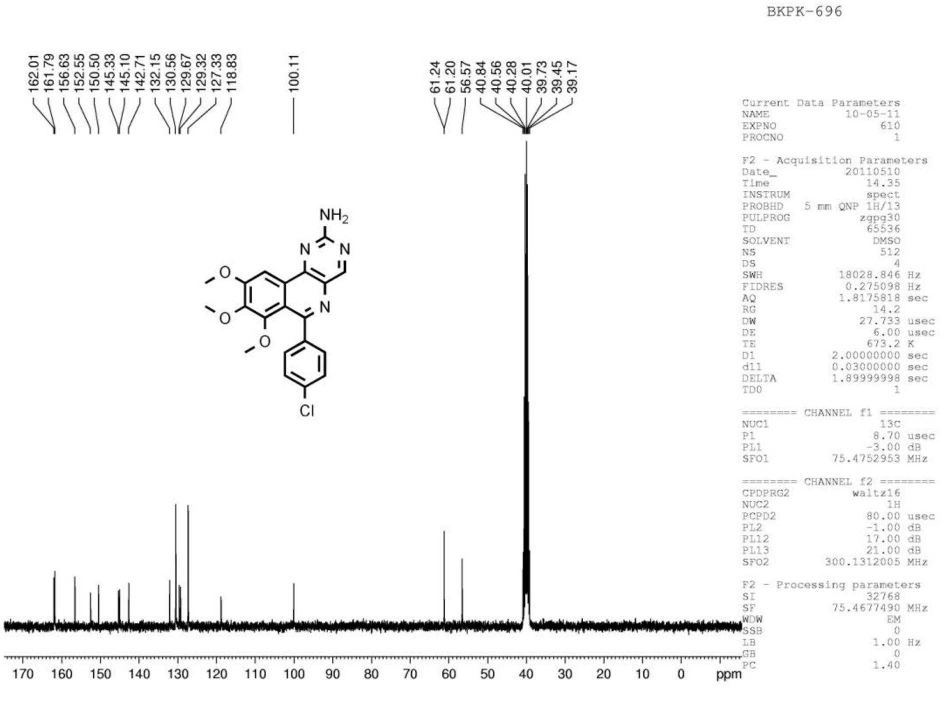

Figure 98: ^{13}C NMR of **20e**.

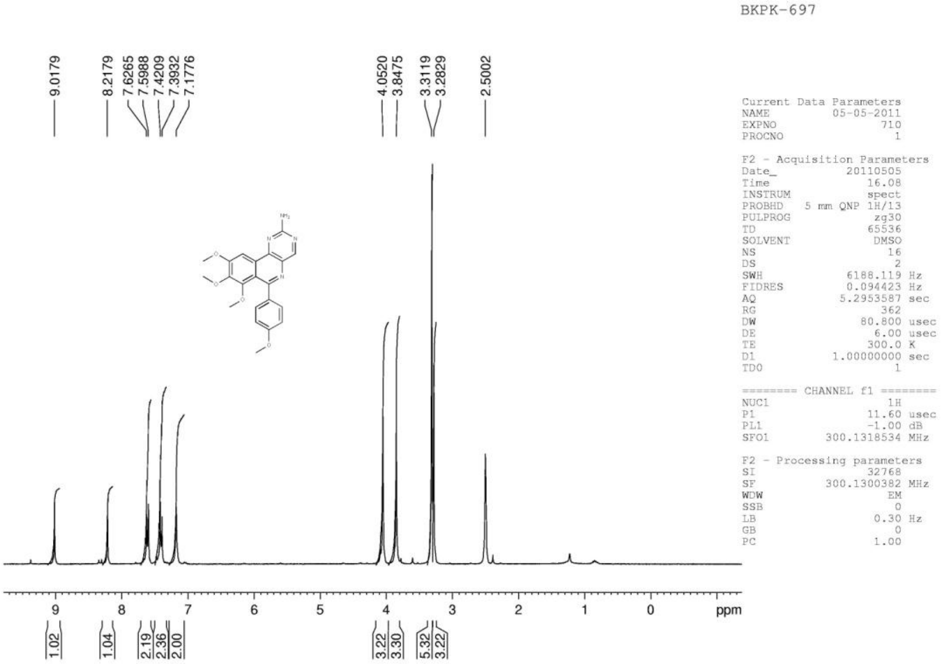

Figure 99: ^{1}H NMR of **20d**.

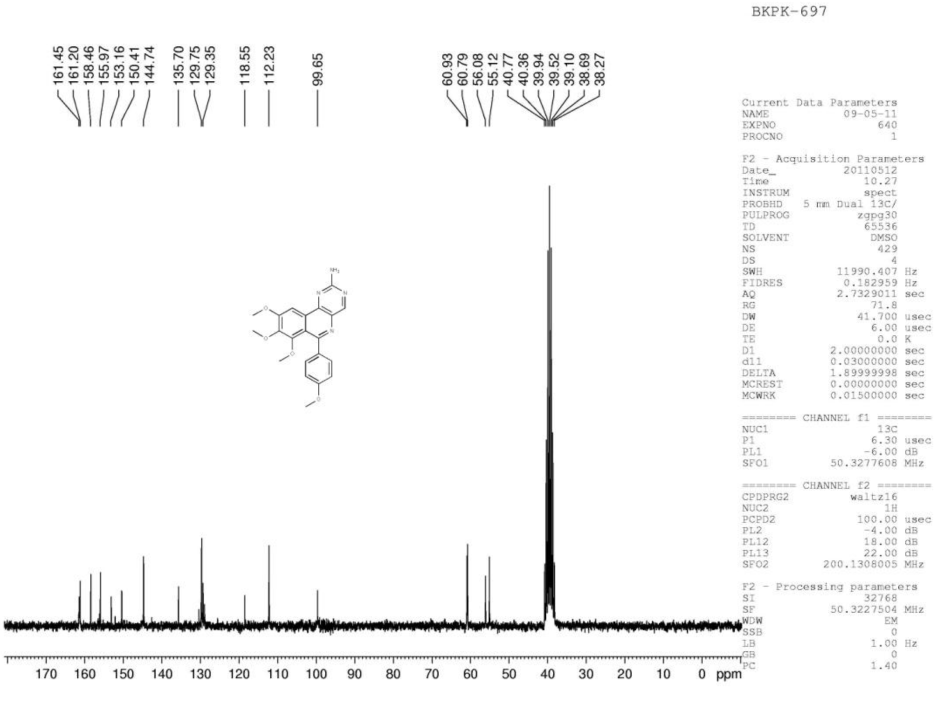

Figure 100: ^{13}C NMR of **20d**.

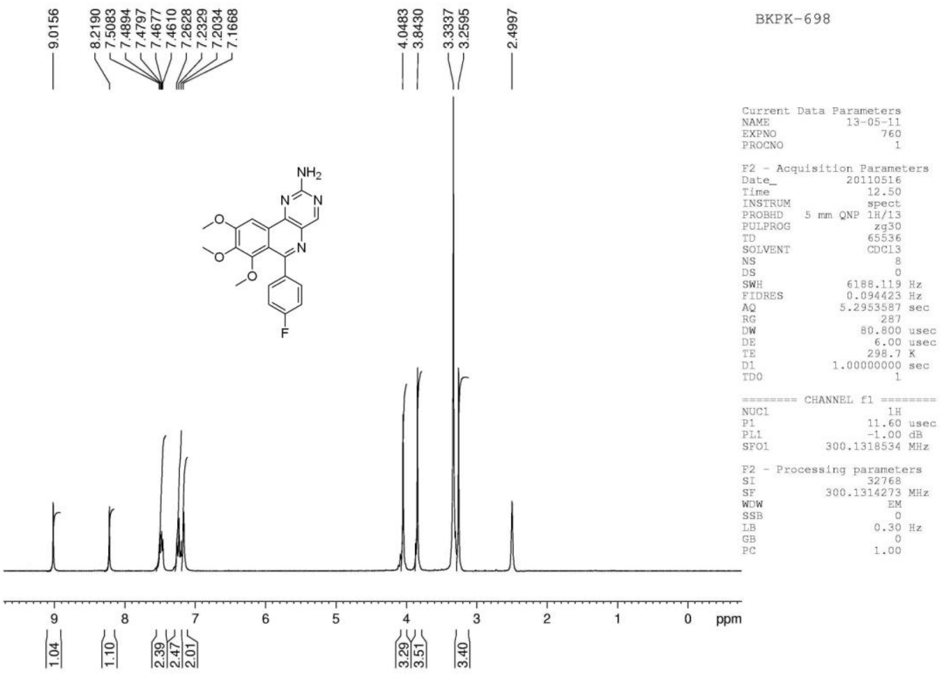

Figure 101: ^{1}H NMR of **20f**.

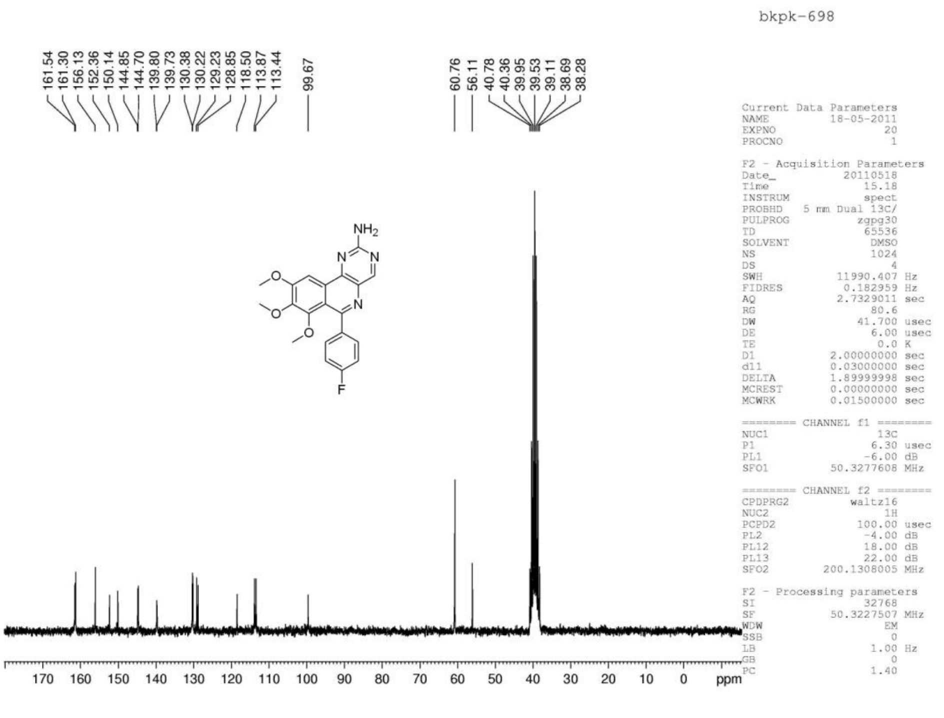

Figure 102: ^{13}C NMR of **20f**.
